# Reactions of Diethylazo‐Dicarboxylate with Frustrated Lewis Pairs

**DOI:** 10.1002/chem.202201701

**Published:** 2022-07-27

**Authors:** Dipendu Mandal, Ting Chen, Zheng‐Wang Qu, Stefan Grimme, Douglas W. Stephan

**Affiliations:** ^1^ Institute of Drug Discovery Technology Ningbo University 315211 Zhejiang P. R. China; ^2^ Department of Chemistry University of Toronto 80 St. George St M5S3H6 Toronto ON Canada; ^3^ Mulliken Center for Theoretical Chemistry Clausius Institut für Physikalische und Theoretische Chemie Rheinische Friedrich-Wilhelms-Universität Bonn Beringstrasse 4 53115 Bonn Germany

**Keywords:** borane, density functional calculations, diethyl azodicarboxylate, frustrated Lewis pairs, phosphines

## Abstract

Reactions of PAr_3_/B(C_6_F_5_)_3_ (Ar=*o*‐Tol, Mes, Ph) FLPs with diethyl azodicarboxylate (DEAD) afford the corresponding FLP addition products **1**–**3** in which P−N and B−O linkages are formed. In contrast, the reaction of BPh_3_, PPh_3_ and DEAD gave product **4** where P−N and N−B linkages were confirmed. In all cases, other binding modes were computed to be both higher in energy and readily distinguishable by ^31^P and ^11^B NMR parameters. These data illustrate the influence of steric demands and electronic structures on the nature of the products of FLP reactions with DEAD.

## Graphical abstract

**Reactions of phosphines** and B(C_6_F_5_)_3_ or BPh_3_ with diethyl azodicarboxylate (DEAD) afforded products with either P−N/B−O lor P−N/B−N linkages. In all cases, the isomers obtained were computed to be both higher in energy and were readily distinguishable by ^31^P and ^11^B NMR parameters. Steric demands and electronics are shown to impact the nature of these FLP addition products.

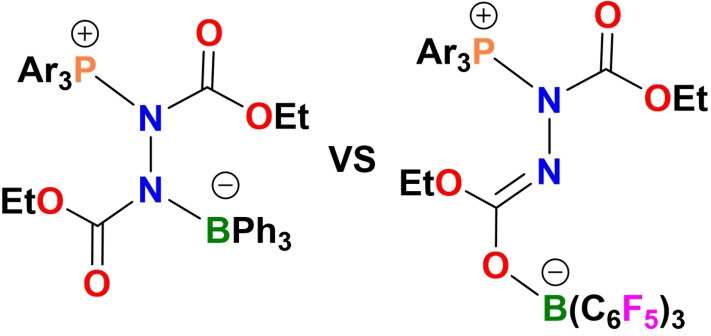

The discovery in 2006 that combinations of sterically congested Lewis acids and bases could reversibly activate H_2_ ^[1]^ dislodged the long‐held chemical dogma that transition metals alone could react with dihydrogen. This observation was subsequently generalized^[2]^ extending the reactivity of so‐called frustrated Lewis pairs (FLPs) to a broad range of main group combinations as well as transition metal reactivity. In addition, the reactivity of FLPs was also extended to a wide variety of small molecules well beyond H_2_. For example, FLPs have been shown to react with CO_2_, CO, SO_2_, N_2_O,^[3]^ olefins,^[2b]^ alkynes,^[4]^ and even C−H bonds.^[5]^ This concept has been extended a wide range of main group Lewis acid /base combinations,^[6]^ transition metal^[7]^ and rare earth systems^[8]^ and most recently to group 1 and 2 metal species^[9]^ clearly illustrating the breadth of this new paradigm.

Early applications of this new reactivity concept focused on the development of metal‐free hydrogenations^[10]^ and a number of applications in organic synthesis have followed.^[11]^ It is also interesting to see the use of FLPs in a range of disparate areas of interest. For example, the concept has been applied to understand the reactivity of enzymatic activation of H_2,_ ^[12]^ and heterogeneous catalysts.^[13]^ FLPs have also been incorporated into MOFs^[14]^ and exploited as catalysts for polymerization.^[15]^

Another creative application of the concept of FLPs has been reported by the Shaver group.^[16]^ These researchers prepared polymers functionalized with either Lewis acidic 4‐styryl‐diphenylborane or Lewis basic 4‐styryl‐diphenylphosphine phosphorus fragments. While the combination of these polymers affords FLPs, crosslinking of these polymers was achieved in the presence of diethyl azodicarboxylate (DEAD). The resulting gel materials exhibit the remarkable ability to “self‐heal”. In studying these systems, Shaver et al. showed that the crosslink is derived from FLP addition to DEAD suggesting new B−N and P−N bonds were formed (Scheme 1). This finding was consistent with a previous study by Bourissou and coworkers^[17]^ who reported the analogous reaction of the intramolecular FLP C_6_H_4_P*i*Pr_*2*_(BMes_2_) with DEAD affording the corresponding adduct C_6_H_4_P*i*Pr_*2*_(BMes_2_)(NCO_2_Et)_2_ (Scheme 1) that contains a six‐membered ring derived from the newly formed B−N and P−N bonds.

**Scheme 1:**
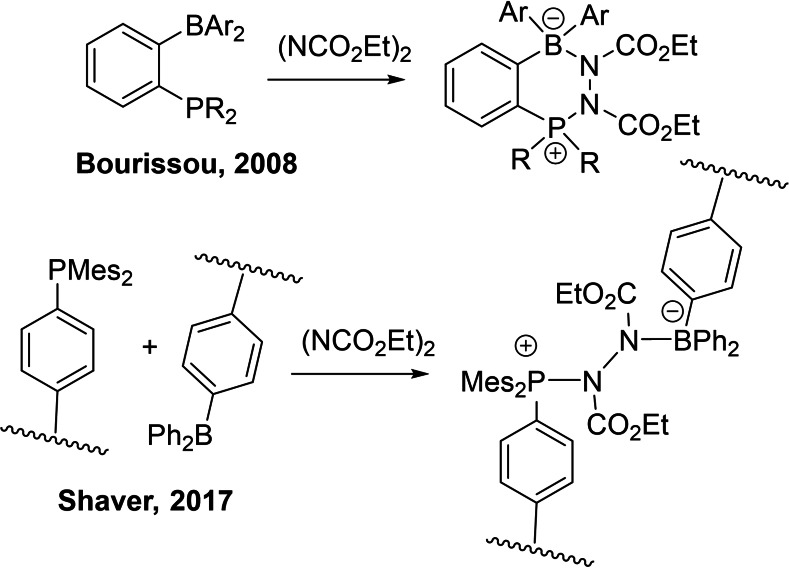
Reactions of DEAD with FLP systems.

Herein, we probe the reactions of DEAD with intermolecular FLPs derived from phosphines and boranes. Experimental and computational data show steric demands and electronic features of the FLP impact the nature of the FLP addition products. In addition, these findings are relevant to the binding mode in previously reported FLP polymers.^[16]^

To the stoichiometric combination of P(*o*‐Tol)_3_/B(C_6_F_5_)_3_ dissolved in CH_2_Cl_2_, DEAD was added and warmed to 40 °C for 24 h. After work‐up, crystallization afforded compound (*o*‐Tol)_3_PN(CO_2_Et)N=C(OEt)OB(C_6_F_5_)_3_, **1** in 88 % yield (Scheme 2). The ^31^P NMR spectrum showed a resonance at 49.0 ppm while ^19^F NMR spectrum gave resonances at −133.8, −161.6 and −166.5 ppm in a ratio of 2 : 1 : 2. The corresponding ^11^B resonance gave a signal at −3.4 ppm. These data are consistent with a phosphorous(V) species and a tetracoordinate boron species. Notably, two sets of ^13^C resonances were observed consistent with inequivalent ester groups. Both the ^1^H and ^13^C NMR spectra of the aryl rings of **1** are inequivalent, consistent with inhibited rotation about the P−C bonds, presumably a result of the steric demands of the aryl substituents.

**Scheme 2:**
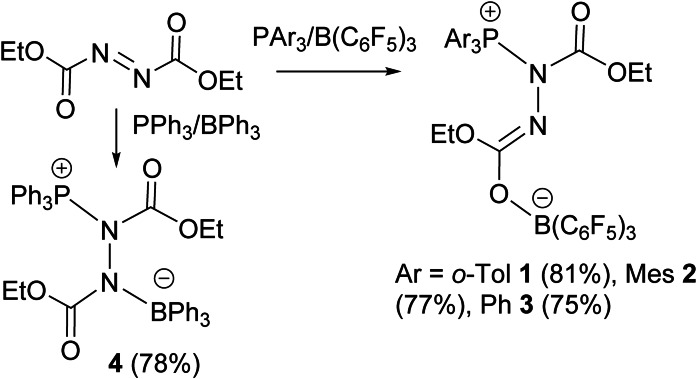
Synthesis of **1**–**4**.

While these data are consistent with the addition of the FLP to DEAD, the precise nature of **1** was confirmed by single crystal XRD (Figure 1). The structure revealed that the phosphine is added to one of the nitrogen atoms of DEAD, while the borane is bound to the ester carbonyl oxygen atom on the adjacent nitrogen atom. This structure gave rise to new P−N and B−O distances of 1.684(1) Å and 1.522(2) Å. The N−N distance was found to be 1.441 (2) Å while the N−C distances were 1.298(2) Å and 1.395(2) Å.

**Figure 1:**
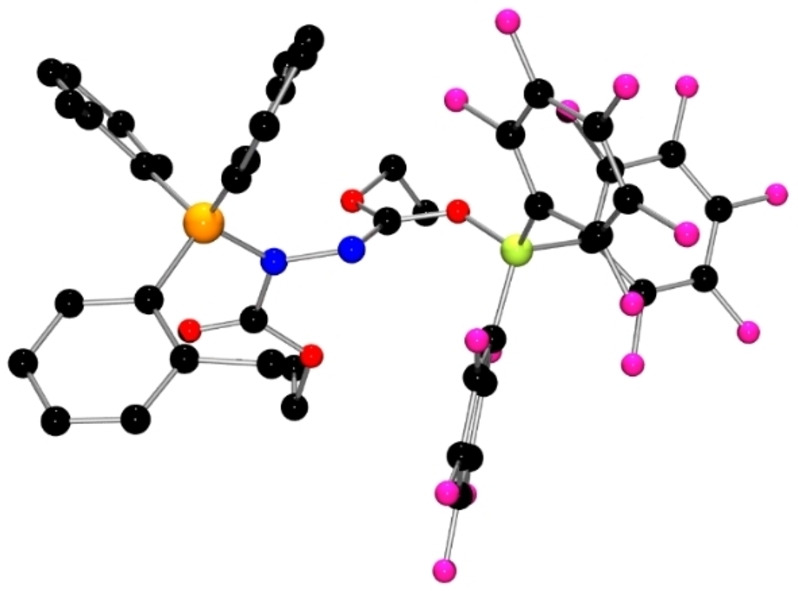
POV‐ray depiction of **1**. Hydrogen atoms are omitted for clarity. P: orange, N: blue, O: red, C: black, F: pink, B: yellow‐green.

The analogous use of Mes_3_P gave a similar reaction, affording a ^31^P signal at 46.1 ppm, a ^11^B signal at −3.5 ppm, and ^19^F resonances at −133.6, −162.1 and −168.6 ppm. The similarity of these data to those observed for **1** prompted the formulation of the product **2** as Mes_3_PN(CO_2_Et)N=C(OEt)OB(C_6_F_5_)_3_ (Scheme 2). This was confirmed crystallographically (Figure 2). Like **1**, the structure of **2** is directly analogous with P−N, N−N and O−B bond lengths of 1.721(1) Å, 1.445(2) Å, and 1.517(2) Å, respectively. Similarly, the reaction of Ph_3_P/B(C_6_F_5_)_3_ with DEAD afforded closely related spectral data and thus the formulation of the product **3** as Ph_3_PN(CO_2_Et)N=C(OEt)OB(C_6_F_5_)_3_ (Scheme 2). It was isolated in 75 % yield and exhibited similar spectroscopic parameters with ^31^P and ^11^B chemical shifts at 45.6 and −3.1 ppm, respectively.

**Figure 2:**
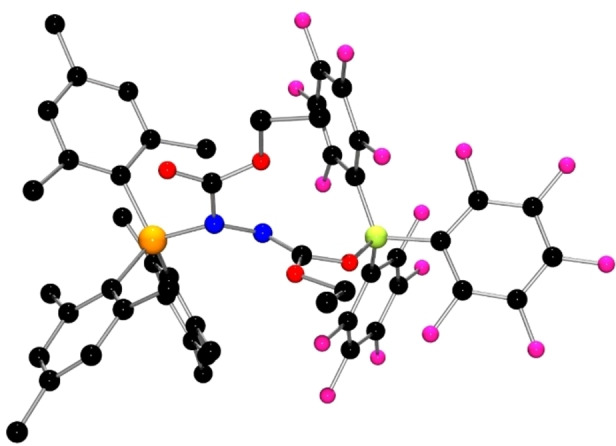
POV‐ray depiction of **2**. Hydrogen atoms are omitted for clarity. P: orange, N: blue, O: red, C: black, F: pink, B: yellow‐green.

It is noteworthy that the structure of **1**–**3** stands in contrast to the DEAD addition products with C_6_H_4_PR_2_(BMes_2_) and Mes_2_PAr/BPh_3_ previously reported by the groups of Bourissou and Shaver, respectively. In the former case the chelating nature of the intramolecular FLP affords a six‐membered ring via the new B−N/P−N bonds to the nitrogen atoms of DEAD, thus presumably enhancing stability of this mode of binding. Bourissou also described the analogous reaction of the intramolecular FLP with PhNCO, where both experimental structures and theoretical calculations supported the notion that steric congestion leads to the thermodynamic favoring of B−O binding.

In the case of the intermolecular polymeric FLP described by Shaver et al., the B−N and P−N binding was proposed based on the observation of broad ^31^P and ^11^B NMR resonances at 44.3 ppm and 6.4 ppm, respectively. The breadth of the signals was interpreted as evidence of a dynamic exchange process. To probe the possibility of dynamic behavior in the present compounds, variable temperature NMR spectra for compound **1** were recorded in CDCl_3_ over the temperatures from 25–65 °C. While both the ^31^P and ^11^B signals sharpened at elevated temperatures, no significant signal shift was observed. Interestingly, dissolution of **1** in d^8^‐toluene shows two ^31^P NMR signals at 49.0 and 51.8 ppm, while the ^11^B NMR signals are observed at −2.4 and −3.7 ppm. The impact of solvent was also examined for the initial mixture of P(*o‐*Tol)_3_, B(C_6_F_5_)_3_ and DEAD. In CDCl_3_ solution, the single ^31^P resonance attributable to **1** was observed. In contrast, in toluene the reaction mixture showed four ^31^P NMR signals at 49.0, 50.2, 51.1 and 53.3 ppm in an intensity ratio of 13 : 9 : 27 : 1 (Figure 3). Efforts to monitor this mixture over time were challenged by the precipitation of **1** from solution.

**Figure 3:**
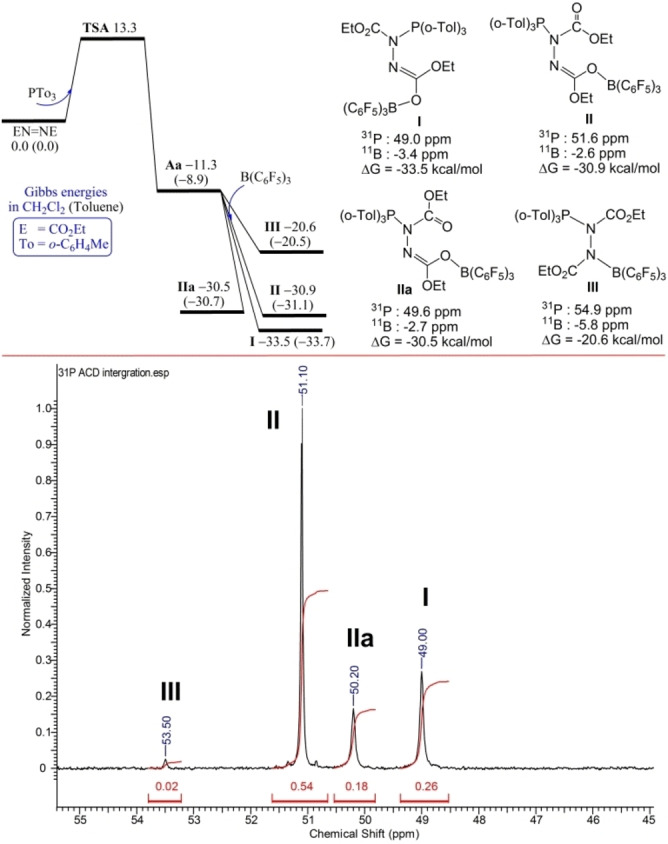
DFT‐computed Gibbs free energy path (at 298 K and 1 M concentration in CH_2_Cl_2_ (or toluene in parentheses), relative to initial FLP and DEAD reactants) and NMR shifts for potential isomers of **1** (top). Experimental ^31^P NMR spectrum for P(*o‐*Tol)_3_, B(C_6_F_5_)_3_ and DEAD in toluene at 40 °C, 24 h (bottom).

To address these observations, DFT computations for the potential isomers of **1** in CH_2_Cl_2_ solution were performed at the PW6B95‐D3/def2‐QZVP+COSMO‐RS//TPSS‐D3/def2‐TZVP+COSMO level of theory.^[18]^ Initial structures generated according to conceivable Lewis structures were checked with the CREST method using the xTB program for low‐lying conformers as input.^[19]^ In addition to DFT‐computed Gibbs energies (at 298 K and 1 M concentration) used in our discussion, the corresponding ^31^P, ^11^B and ^13^C NMR chemical shifts were computed at the GIAO TPSS‐D3/def2‐QZVP level using TPSS‐D3/def2‐TZVP+COSMO optimized geometries (Figure 3, top). The addition of P(*o*‐Tol)_3_ to DEAD is −11.3 kcal/mol exergonic over a low barrier of 13.3 kcal/mol (via **TSA**) to form the P−N adduct **Aa** (see Supporting Information). Further barrierless addition of B(C_6_F_5_)_3_ to **Aa** is −22.2, −19.6, −19.2, and −9.3 kcal/mol exergonic to form the isomers **I**, **II**, **IIa**, and **III** of **1** (Figure 3, top), with a new B−O for the former three and a new B−N bond for **III**. The lowest energy isomer **I** was consistent with the crystallographic data, with the new B−O bond being *cis* to the C=N double bond. Taking the experimentally observed ^31^P and ^11^B for this structure as reliable reference, the DFT‐computed NMR parameters provided useful structural probe for closely related isomers. It is noteworthy that both isomers **II**, **IIa** with the new B−O bond being *trans* to the C=N double bond show different *cis*/*trans* orientation about the amide‐like C−N bond of the free ester carbonyl group (without borane‐binding), which are almost degenerate in free energy but with distinguishable ^31^P NMR signals as observed in experiment; such isomers are about 3 kcal/mol less favored thermodynamically than isomer **I**, but still involve the same P−N/B−O linkages. The isomer **III** involving the B−N/P−N binding mode is significantly less stable than **I** by more than 10 kcal/mol and exhibits different ^31^P and ^11^B chemical shifts at 54.9 and −5.8 ppm, respectively. These data affirm that the P−N/B−O binding mode is thermodynamically favored and readily distinguishable spectroscopically from other isomers. It is interesting to note that the DFT‐computed spectral data fit well with the observations made in solution affording clear assignment of the four ^31^P resonances seen in the initial mixture in toluene to the corresponding isomers, further affirming the preference for the P−N/B−O binding mode.

To probe the impact of the electrophilicity of the borane, the corresponding reaction of the less Lewis acidic BPh_3_, PPh_3_ and DEAD was also monitored spectroscopically. The ^31^P NMR signal was seen at 52.1 ppm, while the ^11^B NMR resonance appeared at 2.0 ppm. This product **4** was isolated in 78 % yield (Scheme 2) and crystallization from toluene affording X‐ray quality crystals. The structure (Figure 4) revealed FLP capture of DEAD via the B−N/N−P binding mode with B−N and P−N bond distances of 1.653(5) Å and 1.693(3) Å.

**Figure 4:**
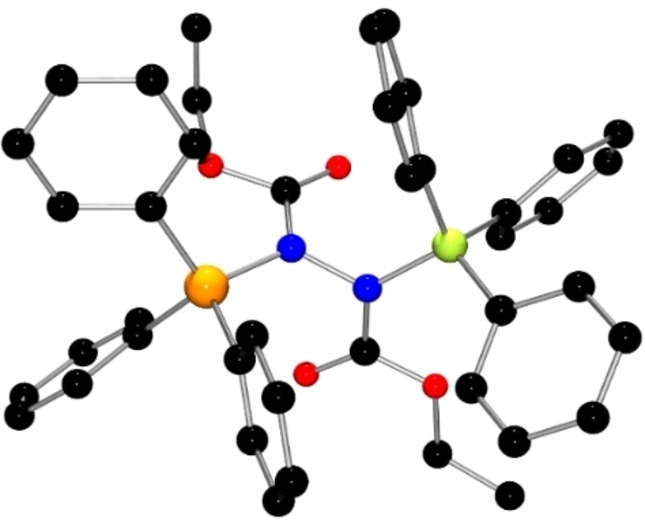
POV‐ray depiction of **4**. Hydrogen atoms are omitted for clarity. P: orange, N: blue, O: red, C: black, B: yellow‐green.

Our DFT calculations show that the addition of less bulky PPh_3_ to DEAD is −16.6 kcal/mol exergonic to form the more stable P−N adduct **Ac** (see Supporting Information). Interestingly, in contrast to the previous cases, further addition of less Lewis acidic BPh_3_ to **Ac** is now −0.3, −5.7 and −7.0 kcal/mol exergonic to form the similar isomers **I**, **II** and **III** of **4** but with a completely reversed energetic order. The observed ^31^P and ^11^B signals at 52.1 and 2.0 ppm for the lowest energy isomer **III** with the B−N/P−N binding mode agree very well with the respective computed values of 52.0 ppm and 0.2 ppm, and it is 1.3 kcal/mol more stable than the B−O/P−N bound isomer **II**.

Probing the previously reported reaction of BPh_2_(C_6_H_4_CH=CH_2_)/PMes_2_(C_6_H_4_CH=CH_2_) and DEAD^[16]^ demonstrates that steric effects alter the outcome of the reaction. In CH_2_Cl_2_ solution, addition of bulky phosphine PMes_2_Ph to DEAD is −5.0 kcal/mol exergonic to form the P−N adduct **Ad** (see Supporting Information); while further addition of BPh_2_(C_6_H_4_CH=CH_2_) to **Ad** is −5.3 and −4.7 kcal/mol exergonic affording the B−O/P−N isomers **I** and **II** but 9.4 kcal/mol endergonic to form the B−N/P−N bound isomer **III** of **5**. In less polar toluene solution, the formation of adduct **Ad** is still −1.1 kcal/mol exergonic while the addition of BPh_2_(C_6_H_4_CH=CH_2_) remains −8.8 and −9.0 kcal/mol exergonic yielding the isomers **I** and **II** but is 6.3 kcal/mol endergonic in the formation of the isomer **III**. The B−N/P−N isomer **III** of the product **5** is thus thermodynamically unfavorable. Furthermore, the experimental ^31^P and ^11^B NMR parameters (44.3, 6.4 ppm) agree very well with DFT‐computed values (48.5, 5.4 ppm) for the isomer **II**, but are quite distinct from those observed for **1** (49.0, −3.4 ppm) and **4** (52.1, 2.0 ppm). These findings suggest that the product of the reaction of BPh_3_/PMes_2_Ph and DEAD is almost certainly the type **II** B−O/P−N isomers.

In conclusion, we have examined the interactions of a series of intermolecular FLPs with DEAD. These data confirm the previously unknown binding mode in which P−N and O−B linkages are formed. DFT computations show that this is the lowest free energy isomer for reactions with B(C_6_F_5_)_3_. In contrast, for reactions with the less Lewis acidic BPh_3_ the steric demand of the phosphine influences the nature of the product. The use of PPh_3_ favors the B−N/P−N products, while bulky PMes_3_ affords the B−O/N−P product. These binding modes are readily distinguished by both experimental and computational ^31^P and ^11^B NMR parameters. Collectively these data clarify the nature of the binding mode of FLPs with DEAD and provide a rare example^[20]^ of the influence of steric demands and electronics on the nature of the products from the FLP capture of a substrate. We are continuing to examine the utility of FLP systems in the capture and reactivity of small molecules.

Supplementary data including synthetic and spectral data, DFT‐computed energies and optimized Cartesian coordinates are deposited.

Deposition Number(s) 2168932 (for **1**), 2168933 (for **2**), 2172267 (for **4**) contain(s) the supplementary crystallographic data for this paper. These data are provided free of charge by the joint Cambridge Crystallographic Data Centre and Fachinformationszentrum Karlsruhe Access Structures service.

## Conflict of interest

The authors declare no conflict of interest.

## Supporting information

As a service to our authors and readers, this journal provides supporting information supplied by the authors. Such materials are peer reviewed and may be re‐organized for online delivery, but are not copy‐edited or typeset. Technical support issues arising from supporting information (other than missing files) should be addressed to the authors.

Supporting Information

## Data Availability

The data that support the findings of this study are available in the supplementary material of this article.
