# Supplementary material for: Reactions of Diethylazo‐Dicarboxylate with Frustrated Lewis Pairs
Source: Chemistry. 2022 Jul 27;28(53):e202201701. doi: 10.1002/chem.202201701 (PMC9796924; doi:10.1002/chem.202201701)
Supplement: Supplementary file 1 — Supporting Information [file CHEM-28-0-s001.pdf]

Chemistry–A European Journal

Supporting Information

Reactions of Diethylazo-Dicarboxylate with Frustrated Lewis Pairs

Dipendu Mandal, Ting Chen, Zheng-Wang Qu,* Stefan Grimme, and Douglas W. Stephan*

Table of Contents

Experimental **3**

General information for synthesis 3

Synthetic procedures and characterization data 4

Synthesis 1 4

Synthesis 2 5

Synthesis 3 6

Synthesis 4 6

NMR spectra of all the compounds **7**

Compound 1 7

Compound 2 10

Compound 3 13

Compound 4 16

Figures for variable high temperature experiments **18**

Figure S20. ^{31}P NMR (203 MHz) spectra for high temperature analysis for P(*o*-Tol)$_3$/B(C_6F_5)$_3$ with diethyl azodicarboxylate in $CDCl_3$. 18

Figure S21. ^{11}B NMR (161 MHz) spectra for high temperature analysis for P(*o*-Tol)$_3$/B(C_6F_5)$_3$ with diethyl azodicarboxylate in d_8-Toluene. 19

Figure S22. ^{31}P NMR (203 MHz) spectra for variable high temperature study for compound 1 in d_8-toluene 20

Figure S23. ^{11}B NMR (161 MHz) spectrum at 100 ℃ (probe temperature) for compound 1 in d_8-toluene 20

Control reactions **21**

Figure S24.^{31}P NMR (203 MHz) spectrum of the crude mixture of diethyl azodicarboxylate and P(*o*-Tol)$_3$ in DCM after 24 h at RT.......... 21

Figure S25.^{31}P NMR (203 MHz) spectrum of the crude mixture of diethyl azodicarboxylate and P(Mes)$_3$ in DCM after 24 h at RT. 21

Figure S26.^{31}P NMR (203 MHz) spectrum of the crude mixture of diethyl azodicarboxylate and PPh$_3$ in DCM after 24 h at RT. 22

Figure S27.^{11}B NMR (161 MHz) spectrum of the crude mixture of diethyl azodicarboxylate and B(C$_6$F$_5$)$_3$ in DCM after 24 h at RT.......... 22

Figure S28.^{19}F NMR (471 MHz) spectrum of the crude mixture of diethyl azodicarboxylate and B(C$_6$F$_5$)$_3$ in DCM after 24 h at RT.......... 23

Experimental references **24**

DFT computational details **25**

Computational Methods 25

Table S1. DFT computed energies in CH$_2$Cl$_2$ solution 26

Table S2. DFT optimized Cartesian coordinates in CH$_2$Cl$_2$ solution.......... 28

Experimental

General information for synthesis

Experiments were carried under inert conditions using standard Schlenk techniques or a glove box as appropriate. Dichloromethane (DCM, CH_2Cl_2), Toluene ($PhCH_3$) and *n*-hexanes (C_6H_{14}) were dispensed from an MBRAUN Solvent Purification System, deoxygenated by bubbling Ar for 20 min, and stored over 3 Å molecular sieves prior to use. Chloroform-d ($CDCl_3$), dichloromethane-d_2 (CD_2Cl_2), and toluene-d_8 ($C_6D_5CD_3$) solvents were used as received without any purification and those were stored over 4 Å molecular sieves prior to use. Vials and stir bar for reactions were oven-dried overnight before experiments. ^{1}H (500 MHz), ^{19}F (471 MHz), $^{19}F\{^{1}H\}$ (471 MHz), $^{31}P\{^{1}H\}$ (202 MHz), and $^{13}C\{^{1}H\}$ (126 MHz) NMR spectra were run at 298 K on Bruker 500 spectrometers. The chemical shifts (δ, ppm) for ^{1}H and $^{13}C\{^{1}H\}$ NMR spectra are given relative to solvent signals whereas an external reference standards used for $^{31}P\{^{1}H\}$ (85% H_3PO_4), ^{19}F ($CFCl_3$) and $^{19}F\{^{1}H\}$ ($CFCl_3$) NMR spectra. These NMR data are written as: chemical shift, multiplicity (s = singlet, d = doublet, t = triplet, q = quartet, m = multiplet, br = broad), coupling constants (Hz) and integration. The single-crystal *X*-ray data were collected on a Bruker D8 QUEST diffractometer using Cu (60W, Diamond, $\mu K\alpha$ = 12.894 mm^{-1}) micro-focus *X*-ray sources at 150 K. The structure was solved and refined using Full-matrix least-squares based on F^2 with a suite of programs SHELXS and SHELXL[1] compiled in OLEX2.[2] The reagents $B(C_6F_5)_3$[3] and BPh_3[4] were prepared by following literature method or a slight variations thereof. All other reagents were purchased commercially and used as received.

Synthetic procedures and characterization data

Synthesis 1

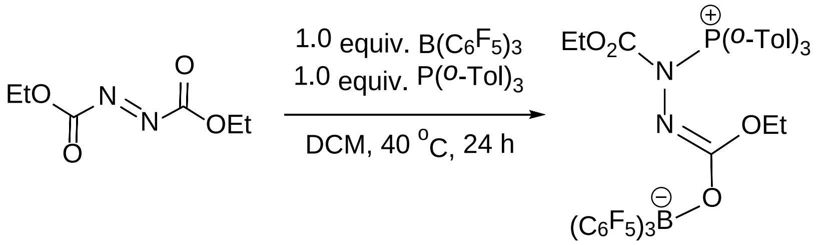

Into a 4 mL open top PTFE vial equipped with a stir bar, $B(C_6F_5)_3$ (51 mg, 0.10 mmol, 1.0 equiv.) and P(*o*-Tol)$_3$ (30 mg, 0.10 mmol, 1.0 equiv.) were dissolved in DCM (0.5 mL). After addition of a solution of diethyl azodicarboxylate (17.4 mg, 0.10 mmol, 1.0 equiv.) in DCM (0.5 mL), the reaction mixture was allowed to heat at 40 ℃ for 24 h. After removal of all volatiles, the residue was washed with *n*-hexane (3 x 1 mL). Further, the residue was crystallized with a mixture of solvent of DCM:*n*-hexane (1:5) and stored at -30 °C for three days. The crystals were collected and dried in affording compound **1** (80 mg, 81%). **1**: ^{1}H NMR (500 MHz, CD_2Cl_2): δ_H 8.41 - 8.22 (m, 1 H, Ar-*H*), 7.76 – 7.47 (m, 5 H, Ar-*H*), 7.40 – 7.19 (m, 6 H, Ar-*H*), 4.35 (br d, *J* = 5.9 Hz, 2 H, -OC*H*$_2$), 4.02 (br s, 2 H, -OC*H*$_2$), 2.38 (s, 3 H, -C*H*$_3$ of P(*o*-Tol)$_3$), 1.79 (s, 3 H, -C*H*$_3$ of P(*o*-Tol)$_3$), 1.52 (s, 3 H, -C*H*$_3$ *of* P(*o*-Tol)$_3$), 1.35 (br t, *J* = 6.5 Hz, 3 H, -OCH$_2$C*H*$_3$), 1.06 (br s, 3 H, -OCH$_2$C*H*$_3$); ^{31}P NMR (203 MHz, CD_2Cl_2): δ_P 49.0 (s, 1 P, -*P*(*o*-Tol)$_3$); ^{19}F NMR (471 MHz, CD_2Cl_2): δ_F -133.8 (m, 6 F, *o*-C$_6$*F*$_5$ of -B(C$_6$*F*$_5$)$_3$), -161.6 (m, 3 F, *p*-C$_6$*F*$_5$ of -B(C$_6$*F*$_5$)$_3$), -166.5 (m, 6 F, *m*-C$_6$*F*$_5$ of -B(C$_6$*F*$_5$)$_3$); ^{11}B NMR (161 MHz, CD_2Cl_2): δ_B -3.4 (br s, 1 B, -*B*(C$_6$F$_5$)$_3$); ^{13}C NMR (126 MHz, CD_2Cl_2): δ_C 163.9 (m, N=*C*(OEt)O-), 156.2 (br s, *C*O$_2$Et), 149.4 (br s, -*C*$_6$F$_5$), 147.5 (br s, -*C*$_6$F$_5$), 146.2 (m, C$_{Ar}$), 144.4 (br s, -*C*$_6$F$_5$), 142.7 (m, C$_{Ar}$), 140.0 (br s, -*C*$_6$F$_5$), 138.4 – 138.1 (m, *C*$_{Ar}$), 138.0 – 137.6 (m, *C*$_{Ar}$), 137.2 (d, $^4J_{C-P}$ = 8.0 Hz, C$_{Ar}$), 136.0 (d, $^3J_{C-P}$ = 13.0 Hz, C$_{Ar}$), 136.1 – 135.8 (m, C$_{Ar}$), 135.7 (m, C$_{Ar}$), 135.5 (d, $^4J_{C-P}$ = 7.0 Hz, C$_{Ar}$), 135.1 (d, $^2J_{C-P}$ = 30.0 Hz), 133.9 (d, $^3J_{C-P}$ = 12.4 Hz, *C*$_{Ar}$), 133.7 (d, $^3J_{C-P}$ = 13.3 Hz, *C*$_{Ar}$), 133.2 (d, $^3J_{C-P}$ = 12.4 Hz, *C*$_{Ar}$), 127.4 (d, $^3J_{C-P}$ = 13.5 Hz, *C*$_{Ar}$), 127.2 (d, $^3J_{C-P}$ = 16.0 Hz, *C*$_{Ar}$), 126.7 (d, $^3J_{C-P}$ = 14.0 Hz, *C*$_{Ar}$), 120.5 (d, $^1J_{C-P}$ = 100.6 Hz, *C*$_{Ar}$), 119.2 (d, $^1J_{C-P}$ = 95.9 Hz, *C*$_{Ar}$), 117.4 (d, $^1J_{C-P}$ = 104.7 Hz, *C*$_{Ar}$), 64.6 (s, *C*H$_2$ of OEt),

64.5 (s, CH_2 of OEt), 22.6 (s, CH_3 of P(*o*-Tol)$_3$), 22.2 (s, CH_3 of P(*o*-Tol)$_3$), 22.0 (s, CH_3 of P(*o*-Tol)$_3$), 15.1 (s, CH_3 of OEt), 14.4 (s, CH_3 of OEt).

Synthesis 2

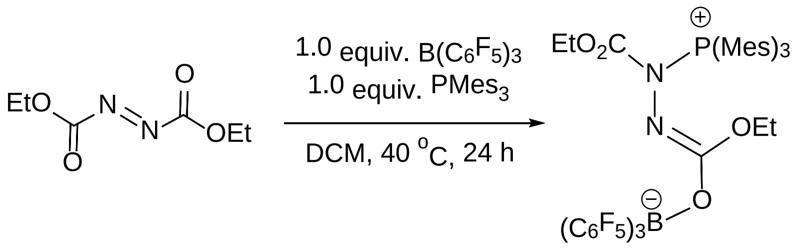

Compound **2** was prepared by following a similar protocol as for compound **1**. **2** (88 mg, 82%): ^{1}H NMR (500 MHz, CD_2Cl_2): δ_H 7.01 (d, *J* = 3.0 Hz, 1 H, Ar-*H*), 6.95 (d, *J* = 3.0 Hz, 1 H, Ar-*H*), 6.84 (d, *J* = 5.3 Hz, 1 H, Ar-*H*), 6.81 – 6.71 (m, 3 H, Ar-*H*), 4.35 (dq, *J* = 10.8, 7.1 Hz, 1 H, -OC*H*$_2$), 4.23 (dq, *J* = 10.8, 7.1 Hz, 1 H, -OC*H*$_2$), 4.01 (dq, *J* = 10.6, 6.8 Hz, 1 H, -OC*H*$_2$), 3.94 (dq, *J* = 10.6, 6.8 Hz, 1 H, -OC*H*$_2$), 2.98 (s, 3 H, -C*H*$_3$ of PMes$_3$), 2.29 (s, 6 H, -C*H*$_3$ of PMes$_3$), 2.15 (s, 3 H, -C*H*$_3$ of PMes$_3$), 2.02 (s, 6 H, -C*H*$_3$ of PMes$_3$), 1.83 (s, 6 H, -C*H*$_3$ of PMes$_3$), 1.77 (s, 3 H, -C*H*$_3$ of PMes$_3$), 1.28 (t, *J* = 7.2 Hz, 3 H, -OCH$_2$C*H*$_3$), 1.02 (t, *J* = 6.7 Hz, 3 H, -OCH$_2$C*H*$_3$); ^{31}P NMR (203 MHz, CD_2Cl_2): δ_P 46.7 (s, 1 P, -*P*Mes$_3$); ^{19}F NMR (471 MHz, CD_2Cl_2): δ_F -133.7 (m, 6 F, *o*-C$_6$*F*$_5$ of -B(C$_6$*F*$_5$)$_3$), -162.4 (m, 3 F, *p*-C$_6$*F*$_5$ of -B(C$_6$*F*$_5$)$_3$), -166.9 (m, 6 F, *m*-C$_6$*F*$_5$ of -B(C$_6$*F*$_5$)$_3$); ^{11}B NMR (161 MHz, CD_2Cl_2): δ_B -3.5 (br s, 1 B, -*B*(C$_6$F$_5$)$_3$); ^{13}C NMR (126 MHz, CD_2Cl_2): δ_C 161.4 (d, $^3J_{C\text{-}P}$ = 9.2 Hz, N=*C*(OEt)O-), 156.5 (d, $^2J_{C\text{-}P}$ = 14.2 Hz, *C*O$_2$Et), 149.4 (br s, -*C*$_6$F$_5$), 147.6 (br s, -*C*$_6$F$_5$), 146.9 (d, $^3J_{C\text{-}P}$ = 8.2 Hz, C_{Ar}), 146.7 (d, $^2J_{C\text{-}P}$ = 13.8 Hz, C_{Ar}), 146.4 (d, $^3J_{C\text{-}P}$ = 7.2 Hz, C_{Ar}), 146.2 (d, $^3J_{C\text{-}P}$ = 11.2 Hz, C_{Ar}), 145.0 (d, $^4J_{C\text{-}P}$ = 3.2 Hz, C_{Ar}), 144.4 (d, $^4J_{C\text{-}P}$ = 3.5 Hz, C_{Ar}), 144.3 (d, $^4J_{C\text{-}P}$ = 2.2 Hz, C_{Ar}), 144.1 (d, $^3J_{C\text{-}P}$ = 12.6 Hz, C_{Ar}), 142.8 (d, $^2J_{C\text{-}P}$ = 17.6 Hz, C_{Ar}), 138.0 (br s, -*C*$_6$F$_5$), 136.0 (br s, -*C*$_6$F$_5$), 133.2 (d, $^2J_{C\text{-}P}$ = 12.7 Hz, C_{Ar}), 133.2 (d, $^2J_{C\text{-}P}$ = 14.0 Hz, C_{Ar}), 132.7 (d, $^2J_{C\text{-}P}$ = 14.4 Hz), 132.5 (d, $^2J_{C\text{-}P}$ = 12.3 Hz), 131.9 (d, $^2J_{C\text{-}P}$ = 12.8 Hz), 126.4 (d, $^1J_{C\text{-}P}$ = 92.5 Hz, C_{Ar}), 120.6 (d, $^1J_{C\text{-}P}$ = 89.8 Hz, C_{Ar}), 117.9 (d, $^1J_{C\text{-}P}$ = 99.5 Hz, C_{Ar}), 64.5 (s, CH_2 of OEt), 64.3 (s, CH_2 of OEt), 24.6 (d, $^3J_{C\text{-}P}$ = 7.5 Hz, *o*-CH_3 of PMes$_3$), 24.3 (d, $^3J_{C\text{-}P}$ = 4.8 Hz, *o*-CH_3 of PMes$_3$), 24.1 (d, $^3J_{C\text{-}P}$ = 7.5 Hz, *o*-CH_3 of PMes$_3$), 24.0 (d, $^3J_{C\text{-}P}$ = 4.8 Hz, *o*-CH_3 of PMes$_3$),

23.3 (d, $^3J_{C\text{-}P}$ = 4.3 Hz, *o*-CH_3 of $PMes_3$), 21.9 (d, $^3J_{C\text{-}P}$ = 3.2 Hz, *o*-CH_3 of $PMes_3$), 21.3 (s, *p*-CH_3 of $PMes_3$), 21.1 (s, *p*-CH_3 of $PMes_3$), 20.9 (s, *p*-CH_3 of $PMes_3$), 15.1 (s, CH_3 of OEt), 14.5 (s, CH_3 of OEt).

Synthesis 3

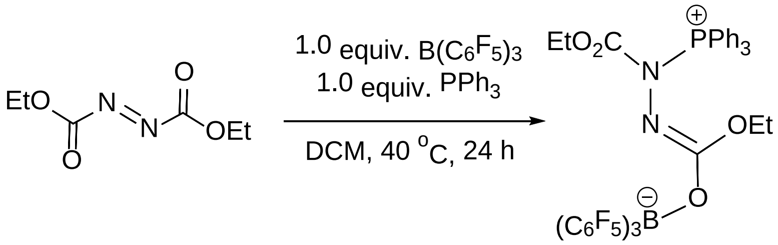

Compound **3** was prepared by following a similar protocol as for compound **1**. After crystallization, an oil appeared followed by drying afforded compound **3** (71 mg, 75%): ^{1}H NMR (500 MHz, $CDCl_3$): δ_H 7.76 – 7.66 (m, 9 H, Ar-*H*), 7.51 – 7.42 (m, 6 H, Ar-*H*), 4.34 (br s, 1 H, -OCH_2), 4.24 (br s, 1 H, -OCH_2), 3.92 (br s, 1 H, -OCH_2), 3.53 (br s, 1 H, -OCH_2), 1.30 (t, *J* = 7.3 Hz, 3 H, -OCH$_2$CH_3), 0.79 (t, *J* = 6.9 Hz, 3 H, -OCH$_2$CH_3); ^{31}P NMR (203 MHz, CD_2Cl_2): δ_P 45.6 (s, 1 P, -*P*Ph_3); ^{19}F NMR (471 MHz, $CDCl_3$): δ_F -133.3 (m, 6 F, *o*-C_6F_5 of -B(C_6F_5)$_3$), -160.9 (m, 3 F, *p*-C_6F_5 of -B(C_6F_5)$_3$), -165.7 (m, 6 F, *m*-C_6F_5 of -B(C_6F_5)$_3$); ^{11}B NMR (161 MHz, $CDCl_3$): δ_B -3.1 (br s, 1 B, -*B*(C_6F_5)$_3$); ^{13}C NMR (126 MHz, $CDCl_3$): δ_C 165.5 (d, $^3J_{C\text{-}P}$ = 3.7 Hz, N=*C*(OEt)O-), 156.2 (d, $^2J_{C\text{-}P}$ = 16.1 Hz, *C*O_2Et), 148.7 (br s, -C_6F_5), 146.8 (br s, -C_6F_5), 139.8 (br s, -C_6F_5), 137.9 (br s, -C_6F_5), 134.8 (d, $^4J_{C\text{-}P}$ = 3.0 Hz, C_{Ar}), 134.2 (d, $^3J_{C\text{-}P}$ = 11.1 Hz, C_{Ar}), 129.2 (d, $^2J_{C\text{-}P}$ = 13.8 Hz, C_{Ar}), 119.8 (d, $^1J_{C\text{-}P}$ = 104.9 Hz, C_{Ar}), 64.9 (s, CH_2 of OEt), 64.2 (s, CH_2 of OEt), 13.6 (s, CH_3 of OEt), 13.4 (s, CH_3 of OEt).

Synthesis 4

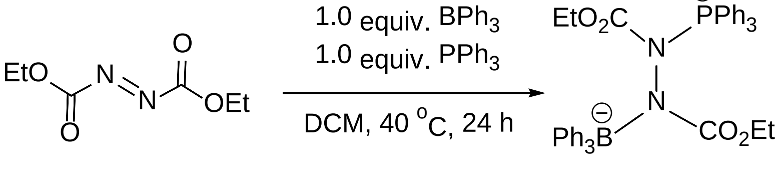

Compound **4** was prepared by following a similar protocol as for compound **1**. After crystallization, an oil appeared followed by drying afforded compound **4** (53 mg, 78%): ^{1}H

NMR (500 MHz, $CDCl_3$): δ_H 8.04 – 7.63 (br m, 6 H, Ar-*H*), 7.60 – 7.34 (br m, 9 H, Ar-*H*), 7.26 – 6.84 (br s, 15 H, Ar-*H*), 4.04 (br m, 1 H, -OC*H*$_2$), 3.77 (br m, 1 H, -OC*H*$_2$), 3.73 (br m, 1 H, -OC*H*$_2$), 3.64 (br m, 1 H, -OC*H*$_2$), 1.05 (br s, 3 H, -OCH$_2$C*H*$_3$), 0.54 (br s, 3 H, -OCH$_2$C*H*$_3$); ^{31}P NMR (203 MHz, $CDCl_3$): δ_P 52.1 (s, 1 P, -*P*Ph$_3$); ^{11}B NMR (161 MHz, $CDCl_3$): δ_B 2.0 (br s, 1 B, -*B*Ph$_3$); ^{13}C NMR (126 MHz, $CDCl_3$): δ_C 163.7 (m, *C*O$_2$Et), 157.7 (br m, *C*O$_2$Et), 156.4 (br, C_{BPh3}), 137.2 (br, C_{BPh3}), 135.0 (br, C_{PPh3}), 134.6 (br, C_{BPh3}), 133.8 (br, C_{BPh3}), 129.0 (d, $^2J_{C\text{-}P}$ = 12.3 Hz, C_{PPh3}), 125.5 (br, C_{PPh3}), 122.7 (br, C_{PPh3}), 64.5 (s, *C*H$_2$ of OEt), 60.7 (s, *C*H$_2$ of OEt), 13.7 (s, *C*H$_3$ of OEt), 13.7 (s, *C*H$_3$ of OEt).

Note: *X*-ray quality crystals for compound **4** were obtained from a crude reaction in toluene instead DCM and that reaction mixture was left undisturbed at RT for 24 h.

NMR spectra of all the compounds

Compound 1

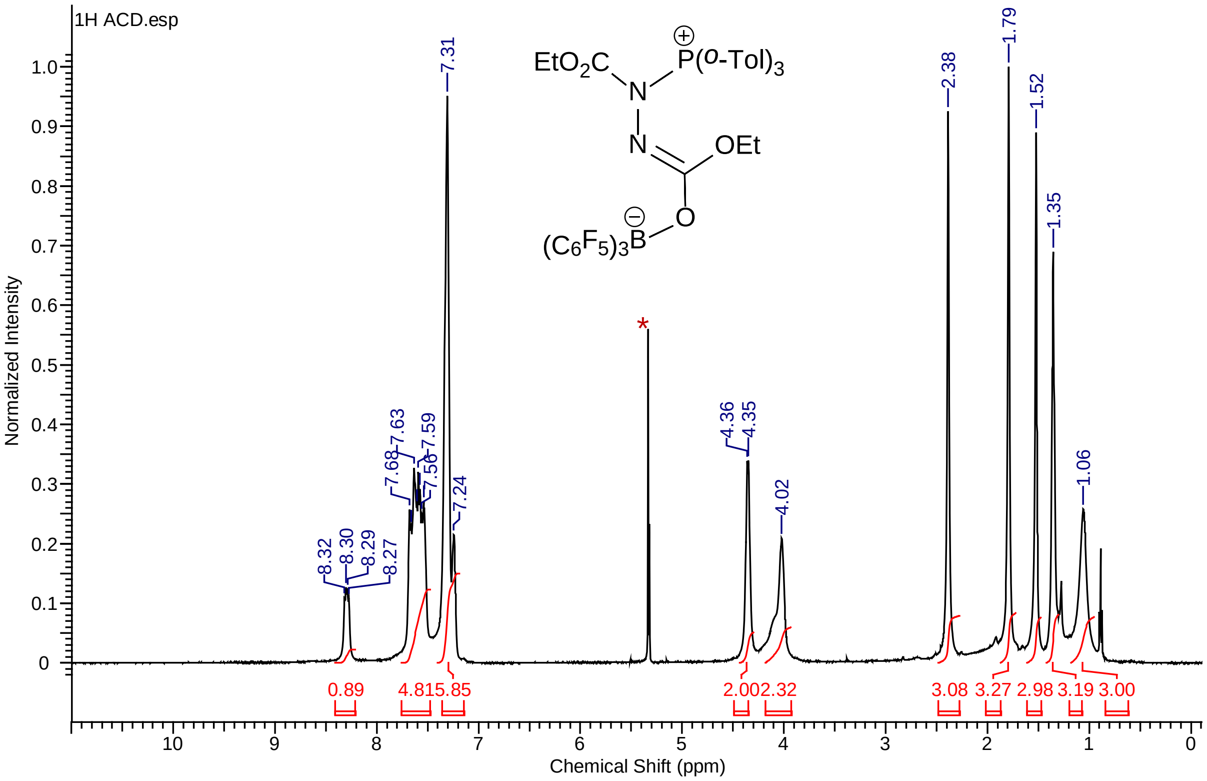

Figure S1. ^{1}H NMR (500 MHz) spectrum of the compound **1** in CD_2Cl_2 (*= CD_2Cl_2).

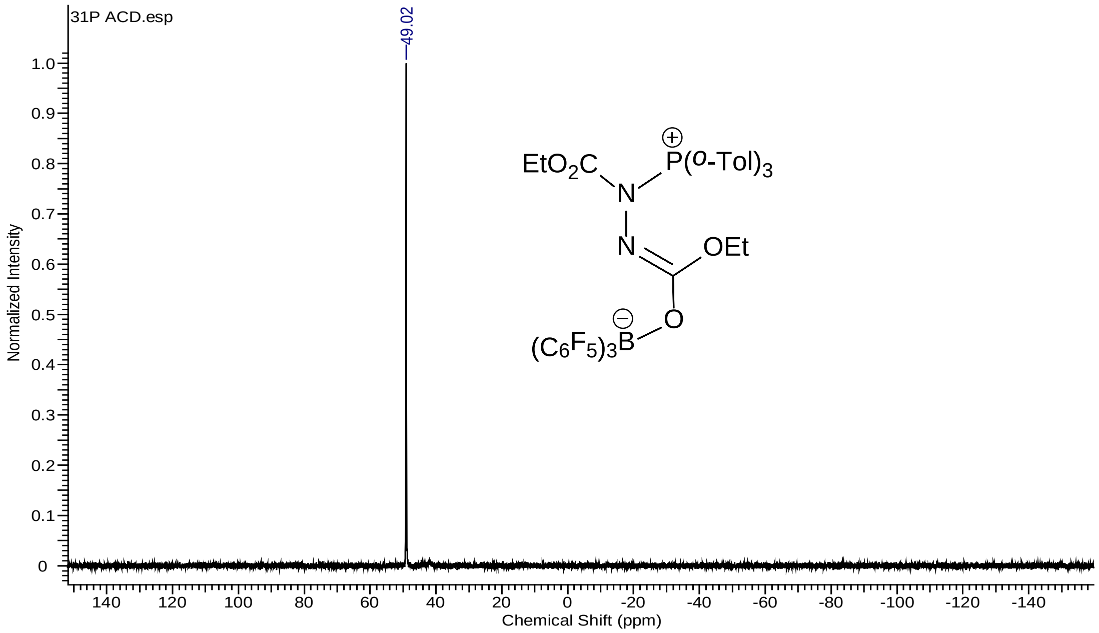

Figure S2. ^{31}P NMR (203 MHz) spectrum of the compound **1** in CD_2Cl_2.

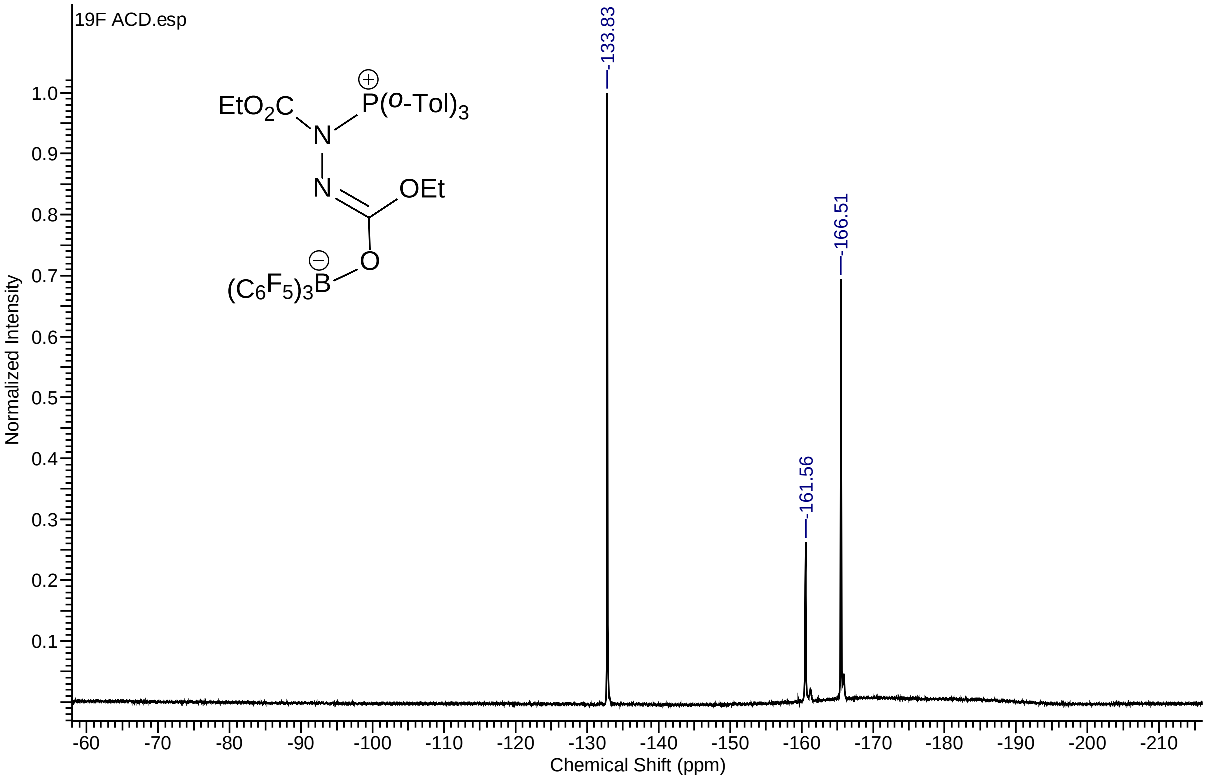

Figure S3. ^{19}F NMR (471 MHz) spectrum of the compound **1** in CD_2Cl_2.

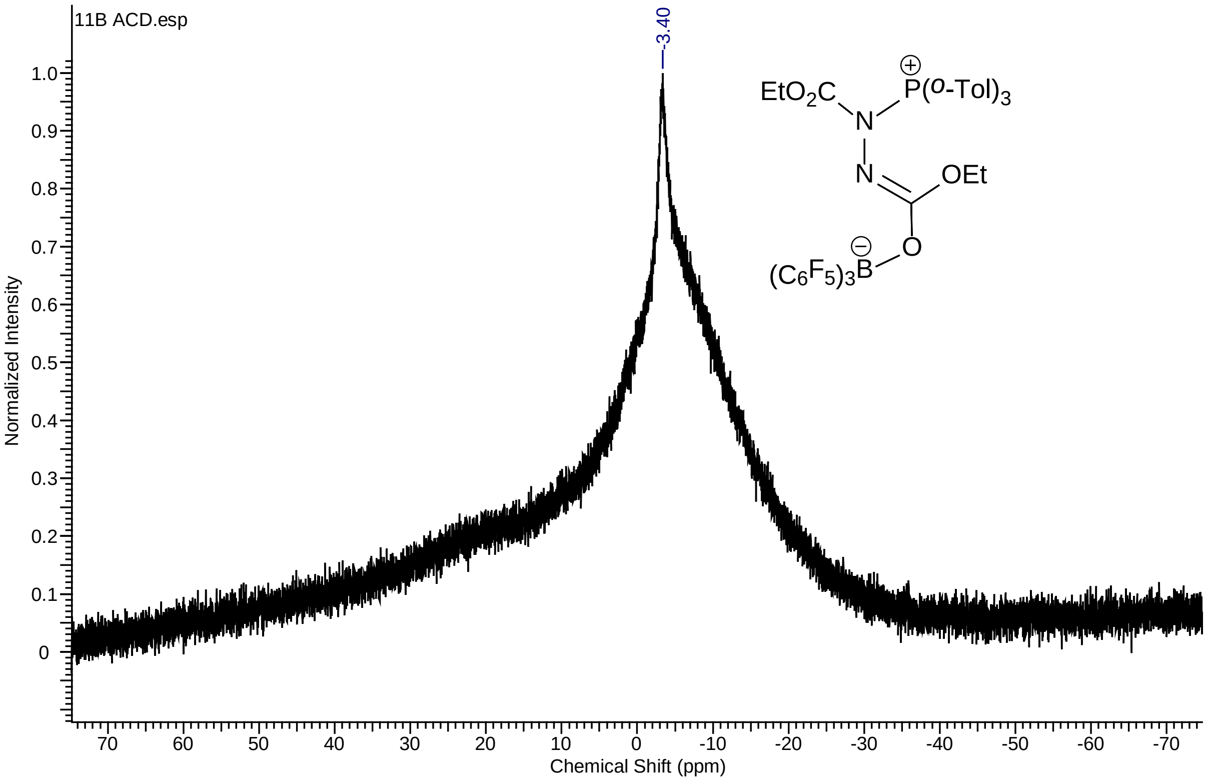

Figure S4. ^{11}B NMR (161 MHz) spectrum of the compound **1** in CD_2Cl_2.

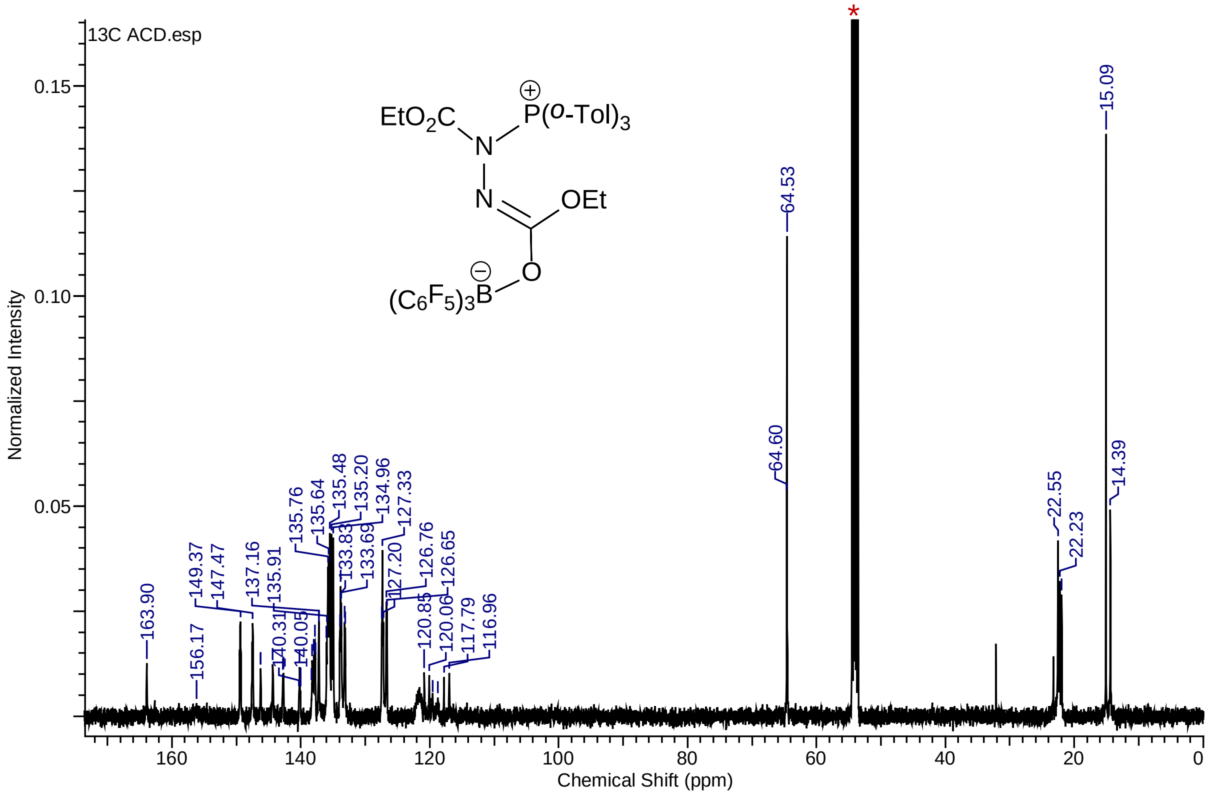

Figure S5. ^{13}C NMR (126 MHz) spectrum of the compound **1** in CD_2Cl_2 (*= CD_2Cl_2).

Compound 2

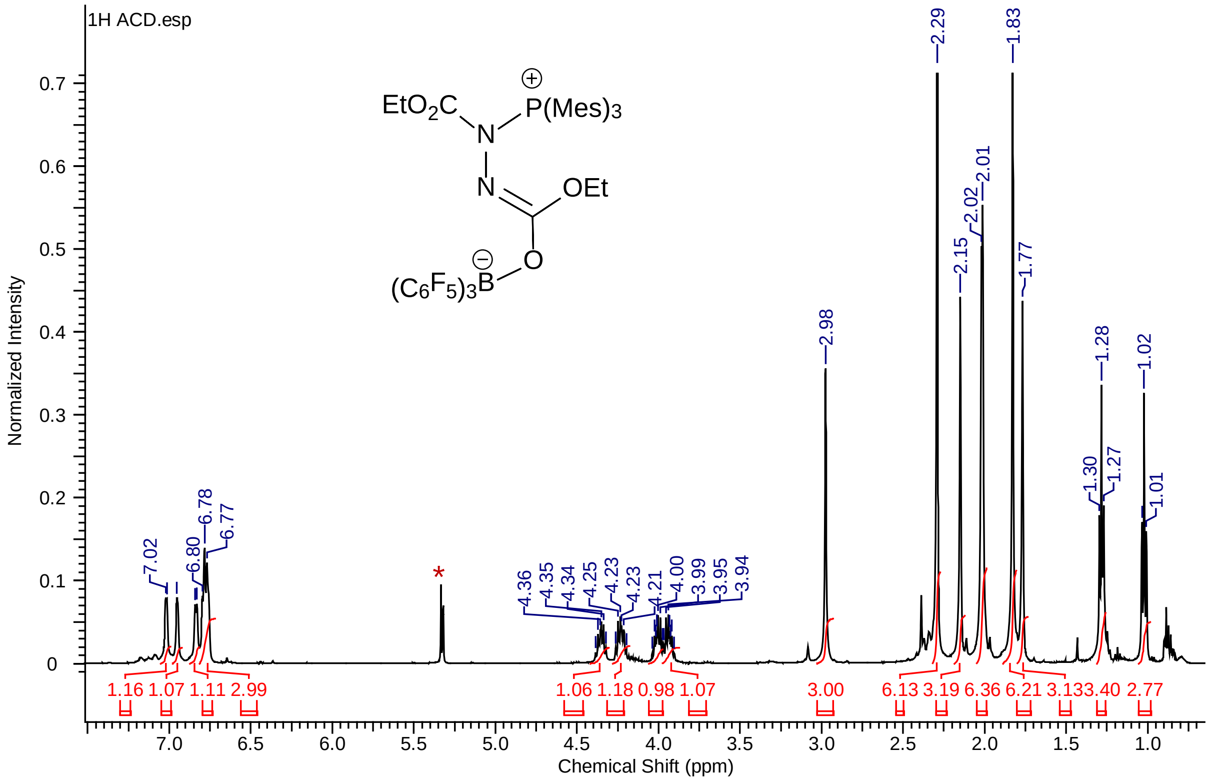

Figure S6. ^{1}H NMR (500 MHz) spectrum of the compound **2** in CD_2Cl_2 (*= CD_2Cl_2).

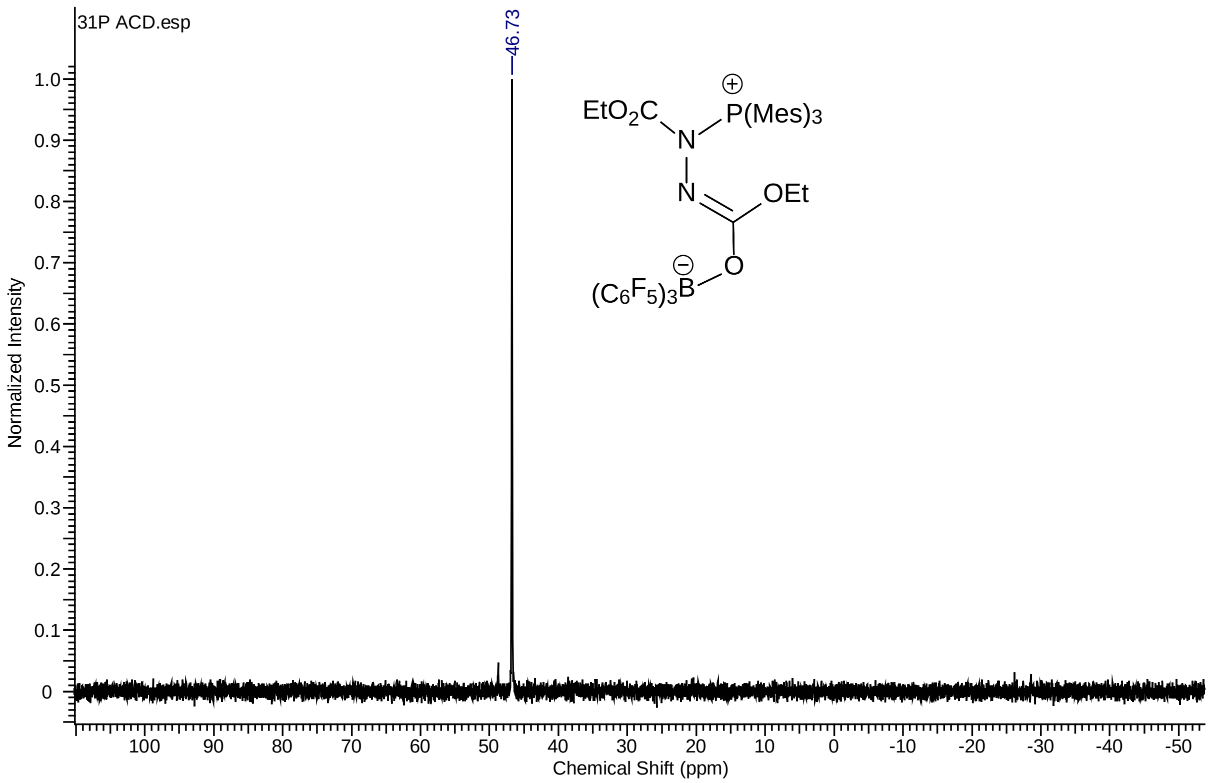

Figure S7. ^{31}P NMR (203 MHz) spectrum of the compound **2** in CD_2Cl_2.

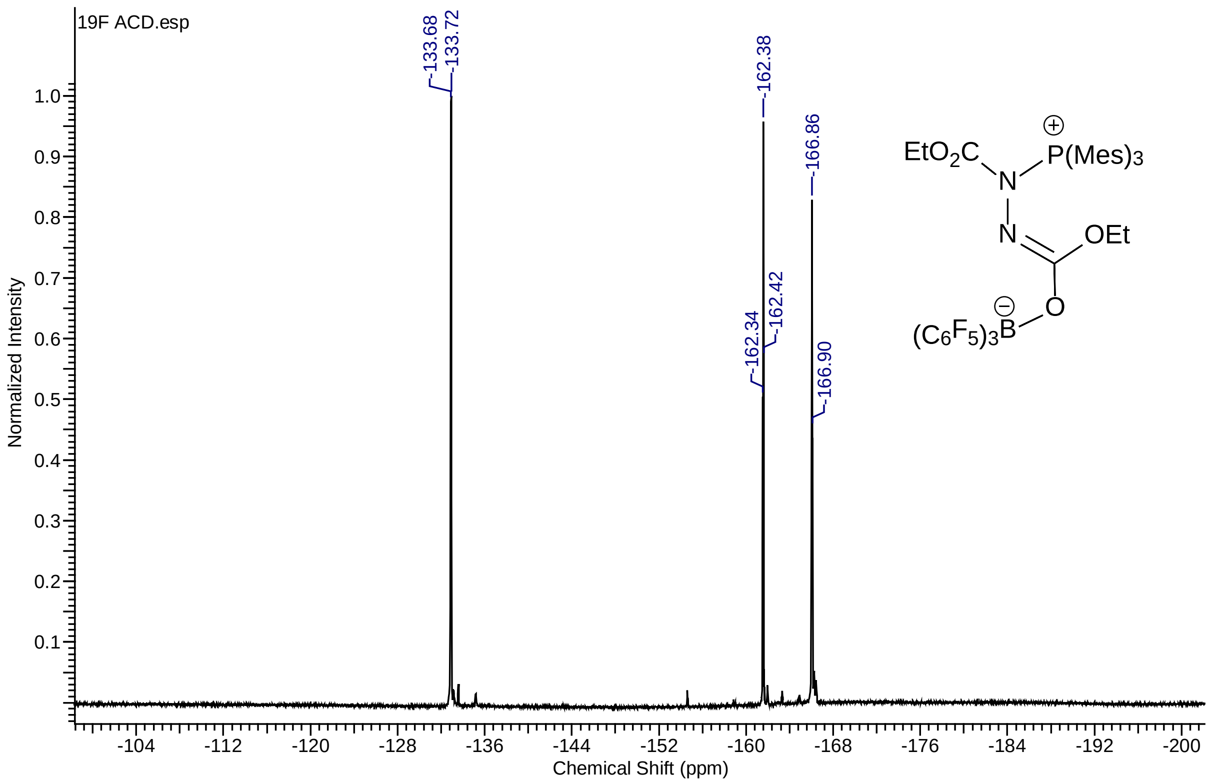

Figure S8. ^{19}F NMR (471 MHz) spectrum of the compound **2** in CD_2Cl_2.

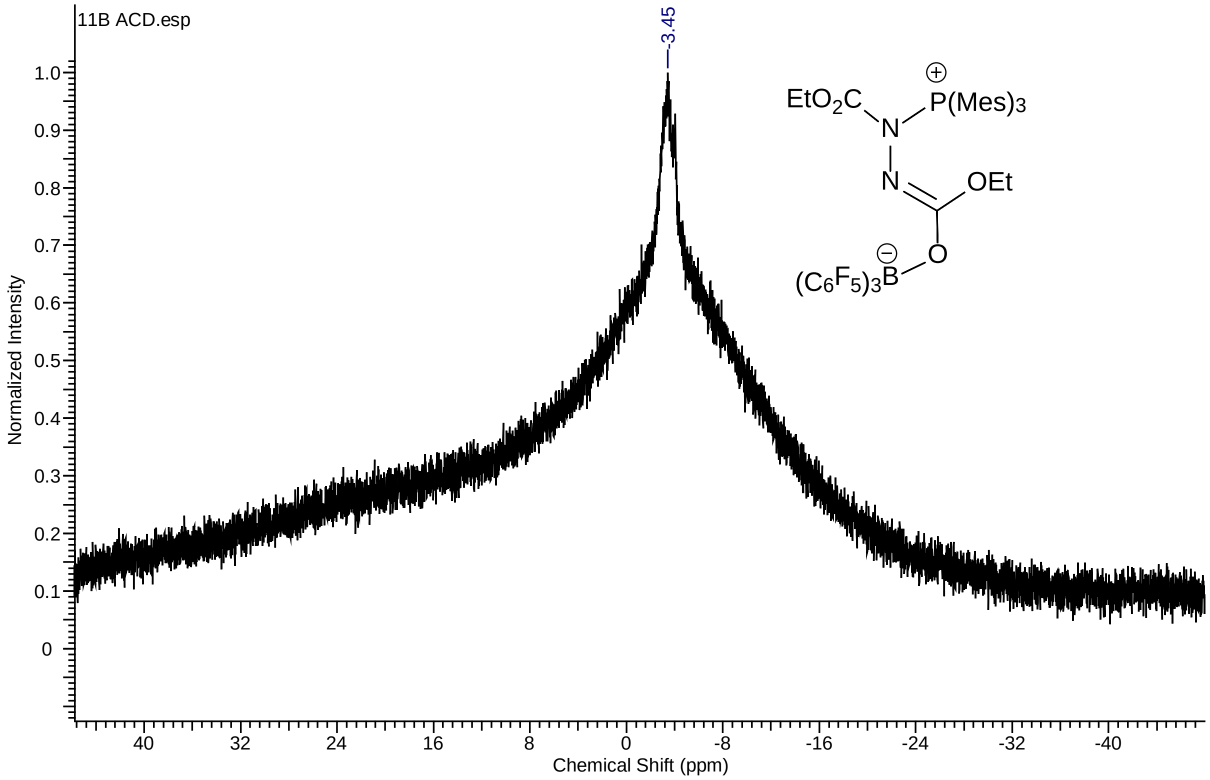

Figure S9. ^{11}B NMR (161 MHz) spectrum of the compound **2** in CD_2Cl_2.

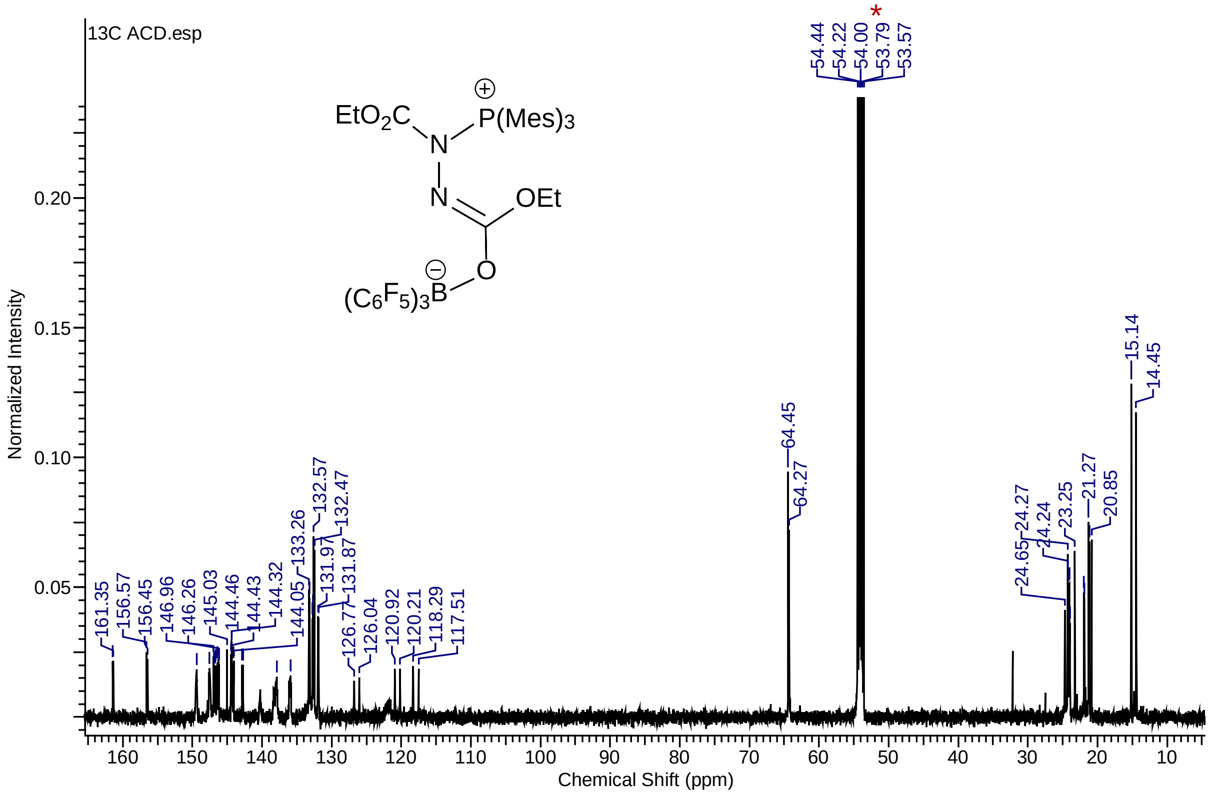

Figure S10. ^{13}C NMR (126 MHz) spectrum of the compound **2** in CD_2Cl_2 (*= CD_2Cl_2).

Compound 3

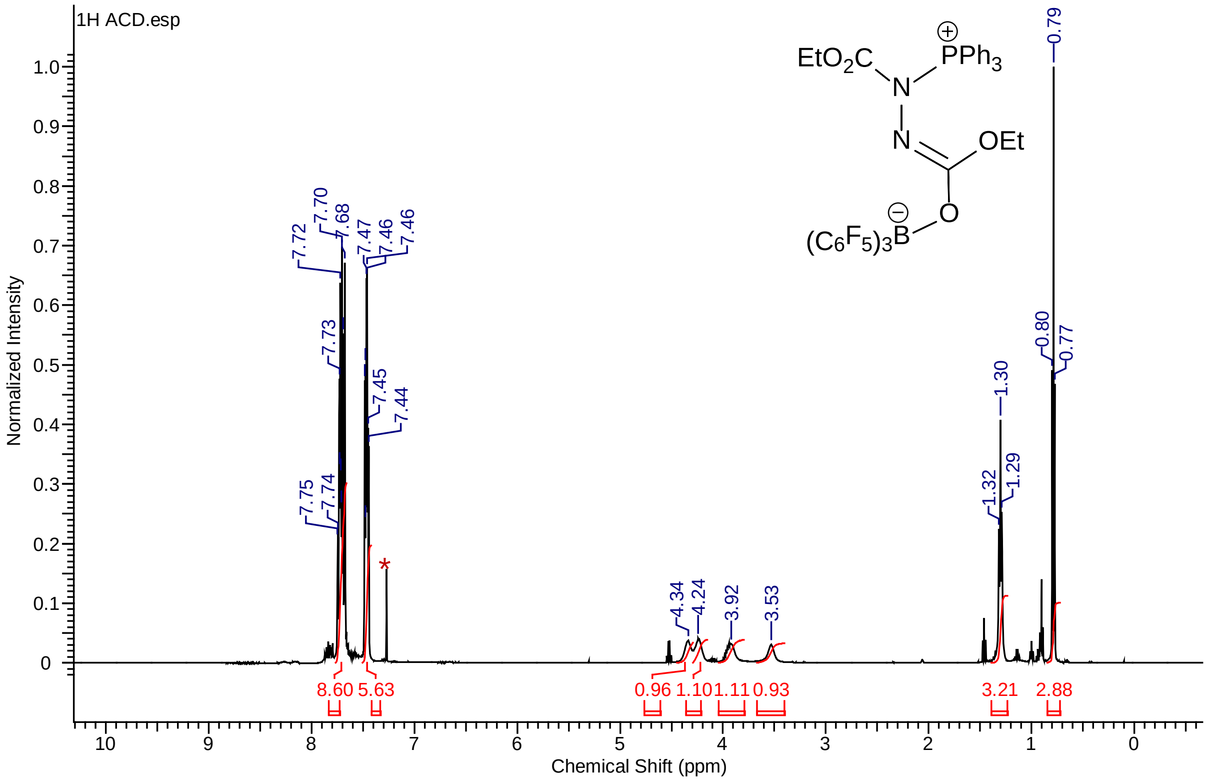

Figure S11. ^{1}H NMR (500 MHz) spectrum of the compound **3** in $CDCl_3$ (*= $CDCl_3$).

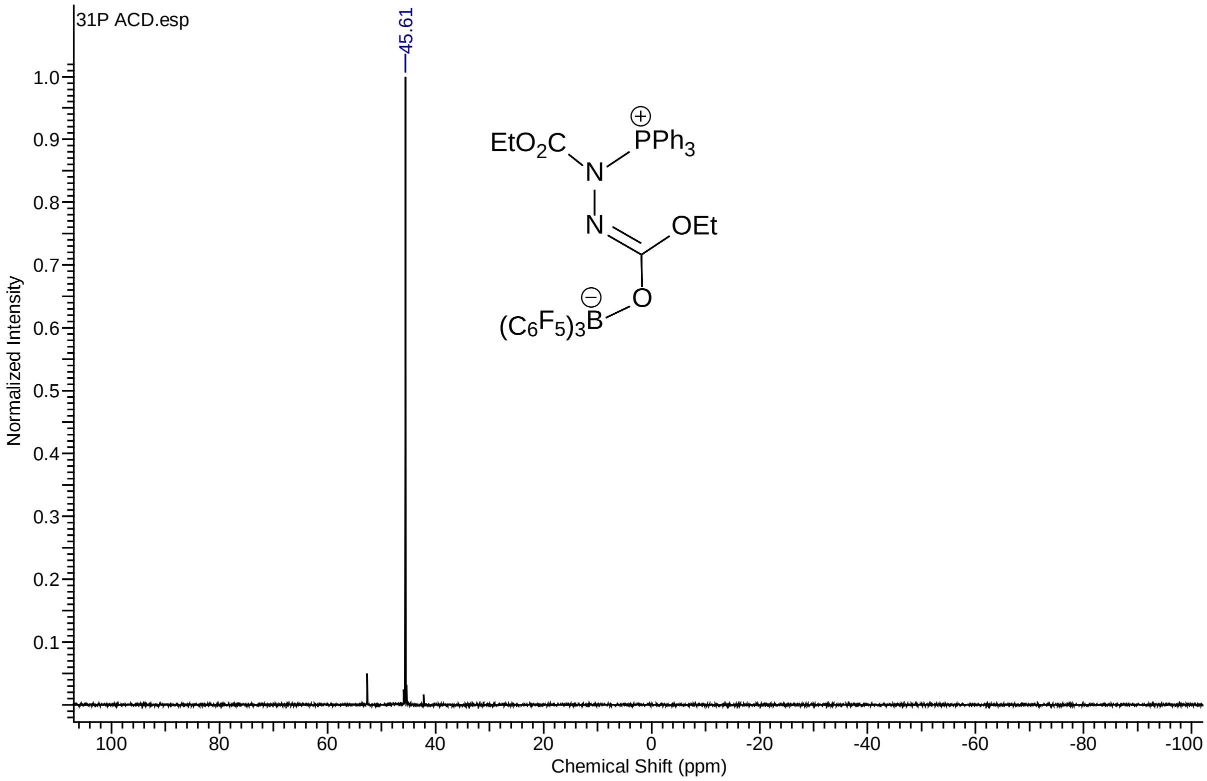

Figure S12. ^{31}P NMR (203 MHz) spectrum of the compound **3** in $CDCl_3$.

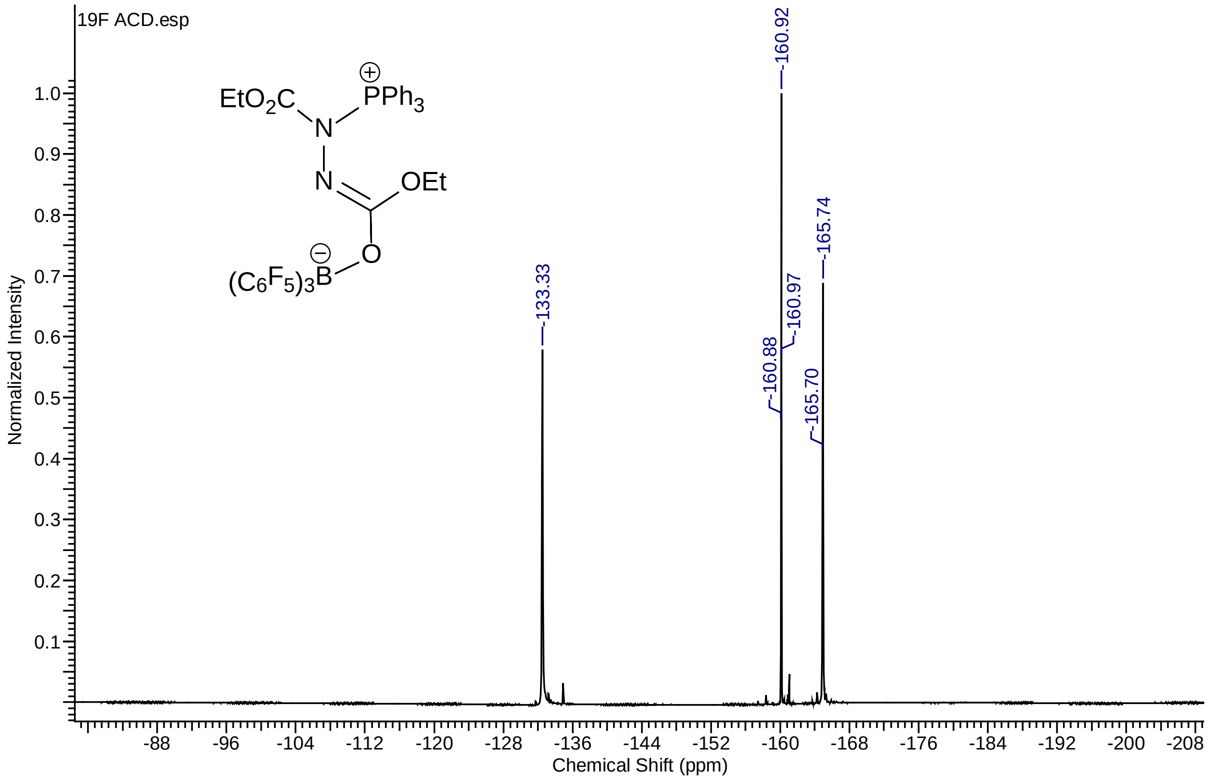

Figure S13. ^{19}F NMR (471 MHz) spectrum of the compound **3** in $CDCl_3$.

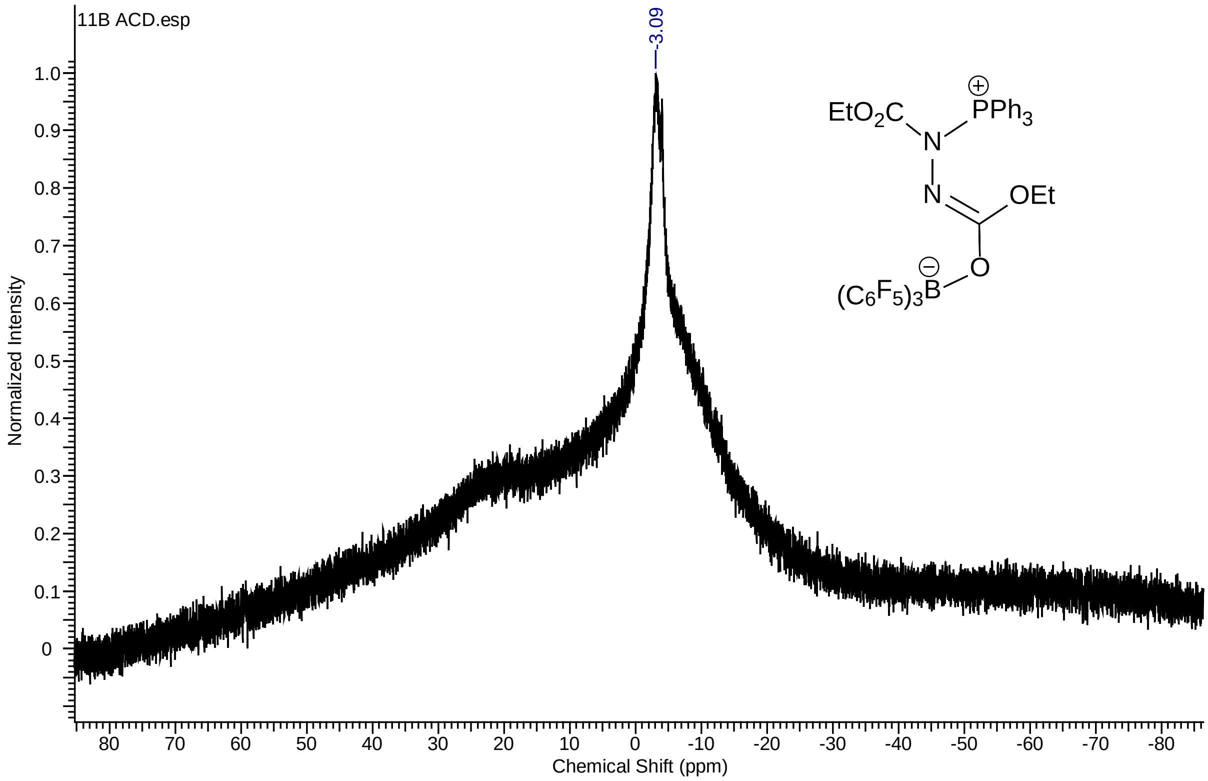

Figure S14. ^{11}B NMR (161 MHz) spectrum of the compound **3** in $CDCl_3$.

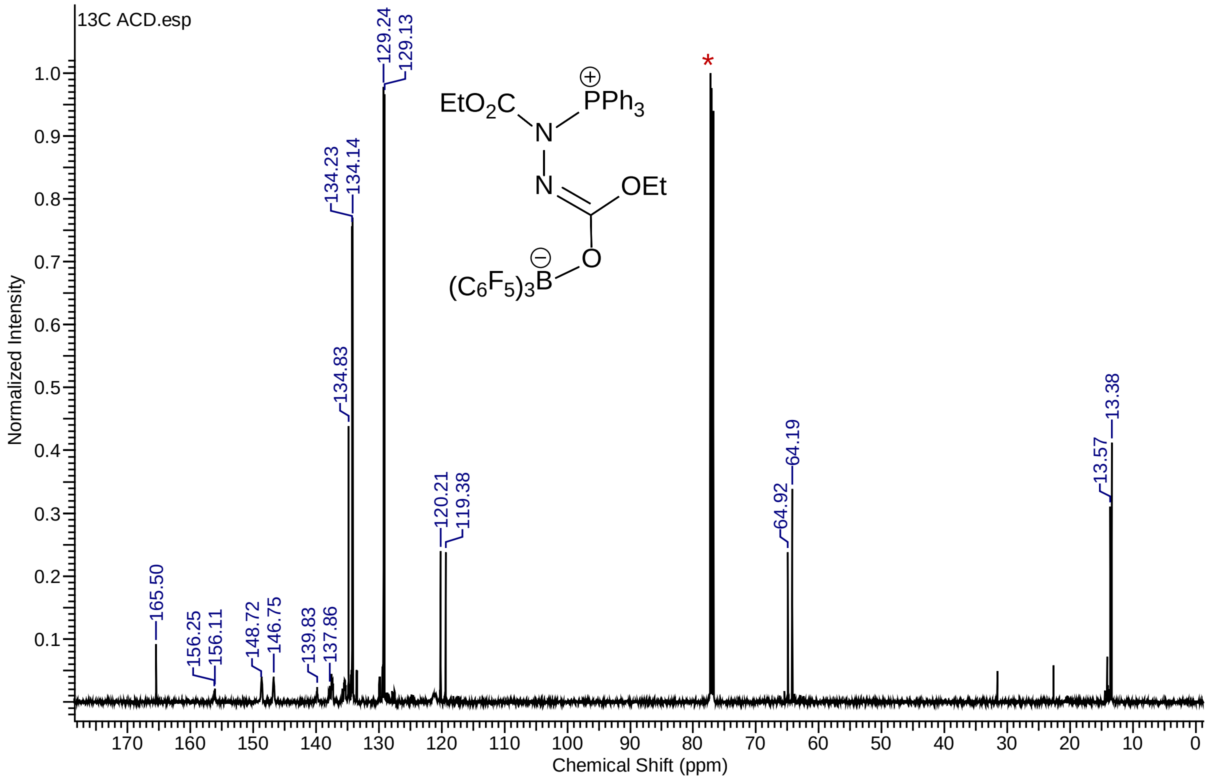

Figure S15. ^{13}C NMR (126 MHz) spectrum of the compound **3** in $CDCl_3$ (*= $CDCl_3$).

Compound 4

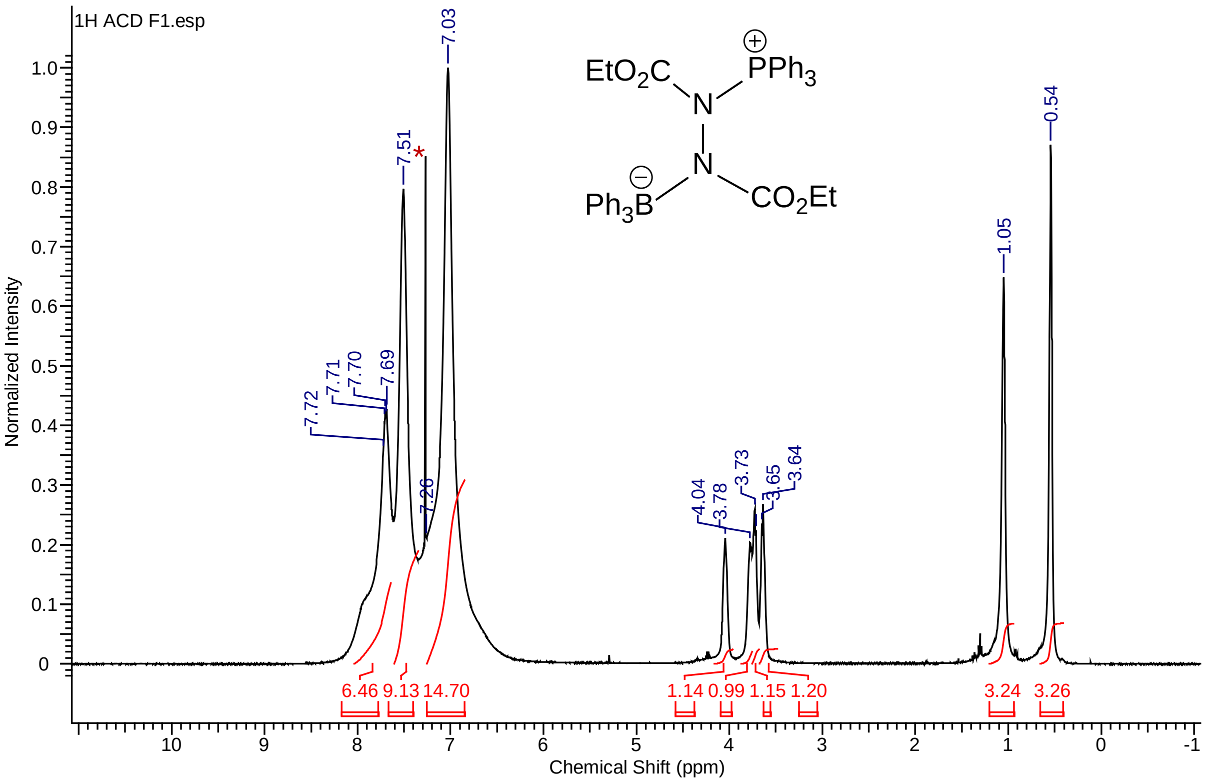

Figure S16. ^{1}H NMR (500 MHz) spectrum of the compound **4** in $CDCl_3$ (*= $CDCl_3$).

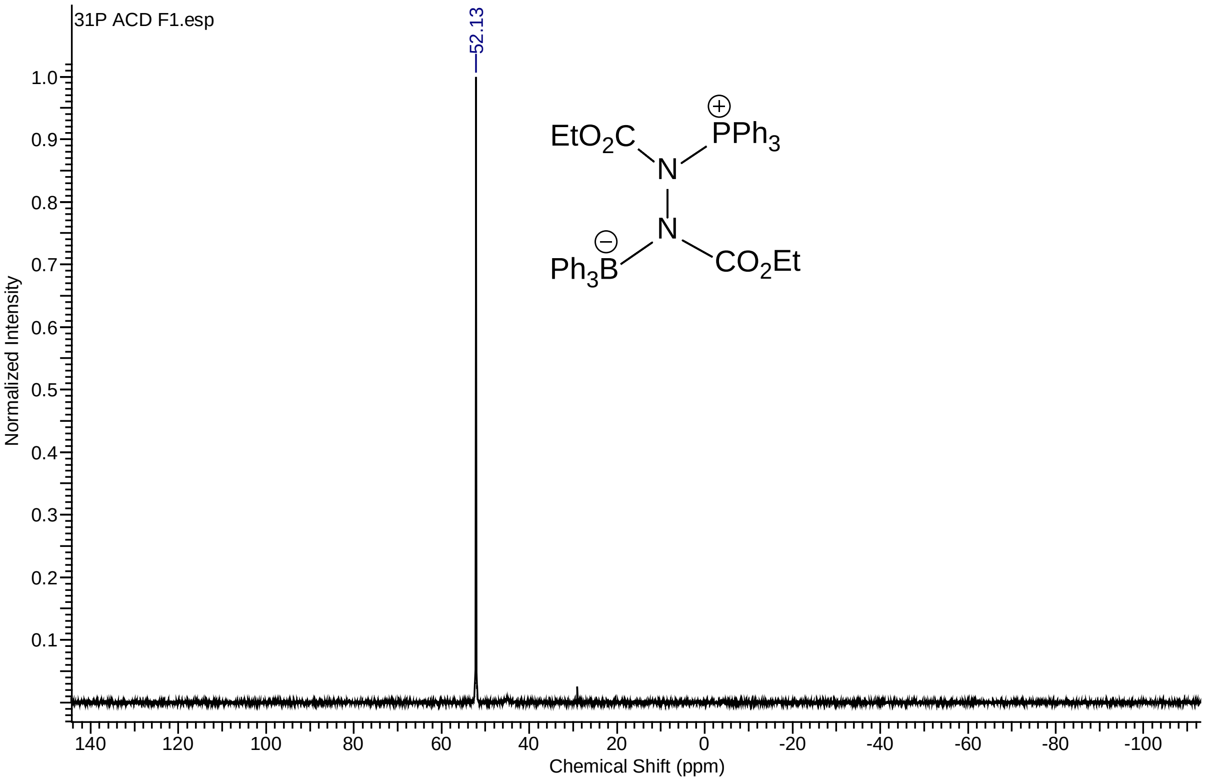

Figure S17. ^{31}P NMR (203 MHz) spectrum of the compound **4** in $CDCl_3$.

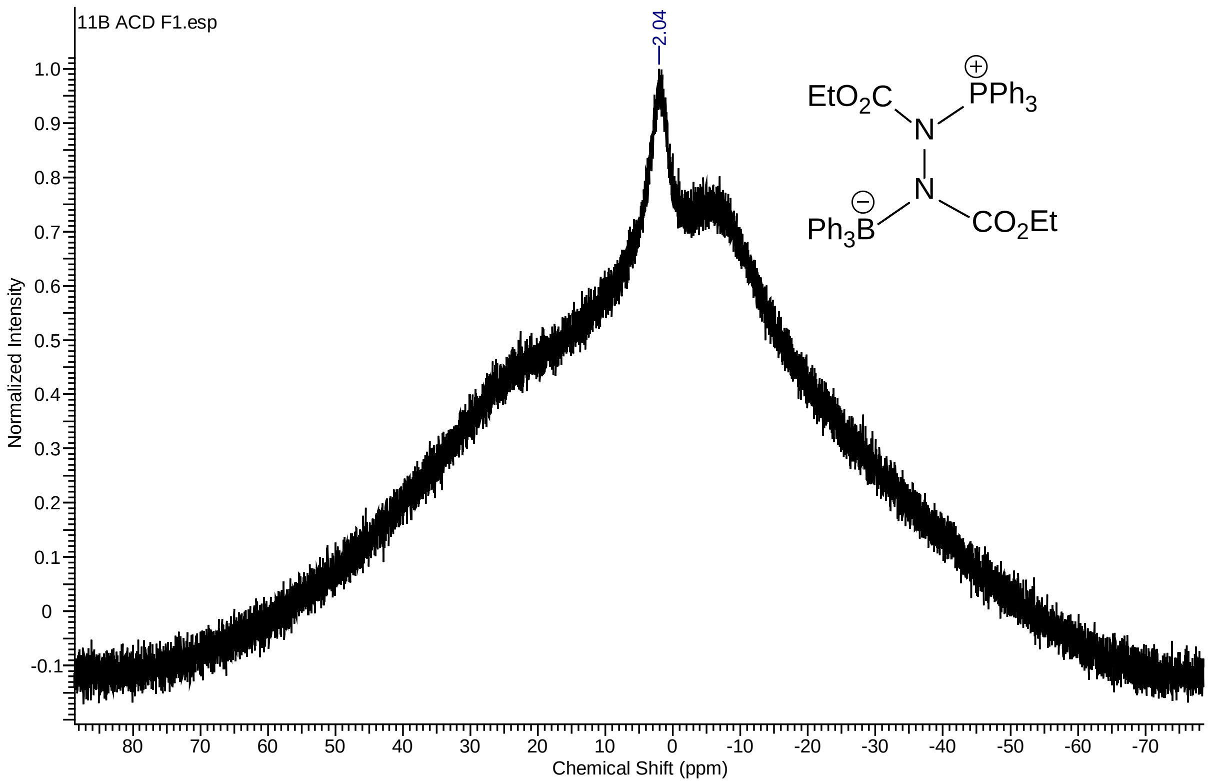

Figure S18. ^{11}B NMR (161 MHz) spectrum of the compound **4** in $CDCl_3$.

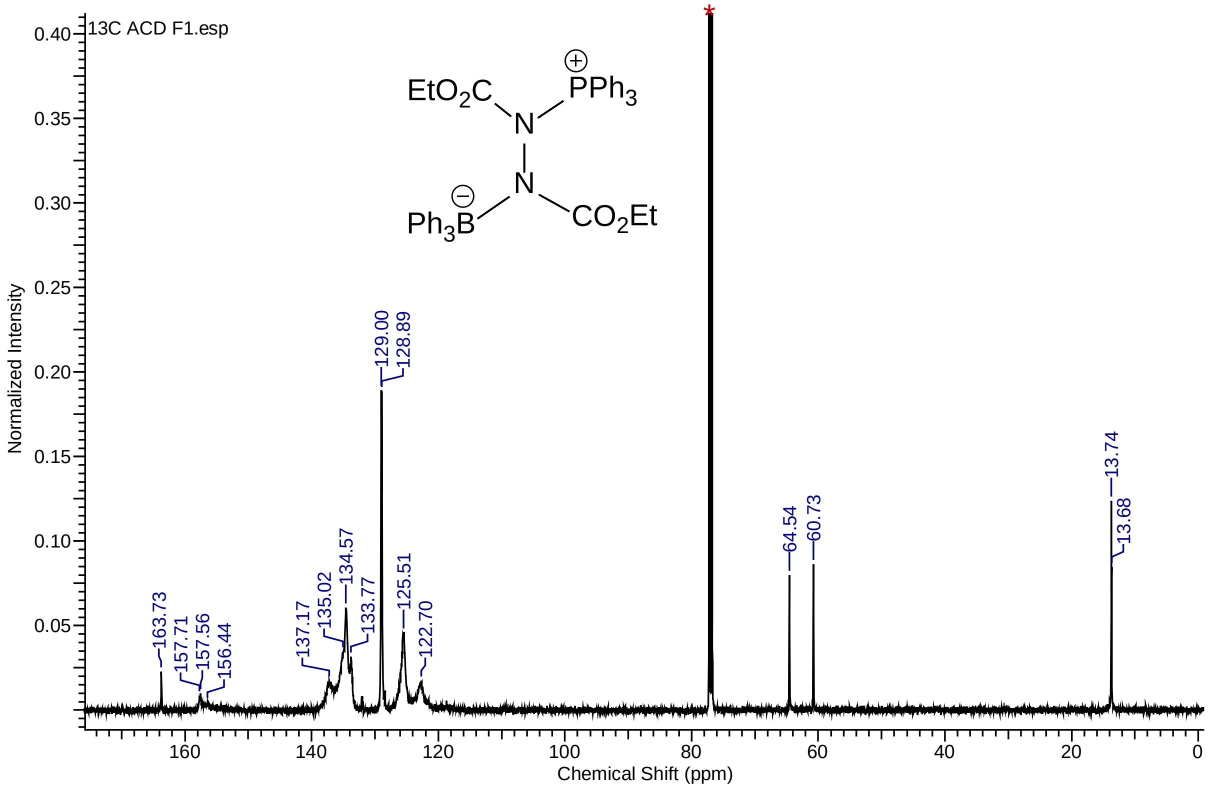

Figure S19. ^{13}C NMR (126 MHz) spectrum of the compound **4** in $CDCl_3$ (*= $CDCl_3$).

Figures for variable high temperature experiments

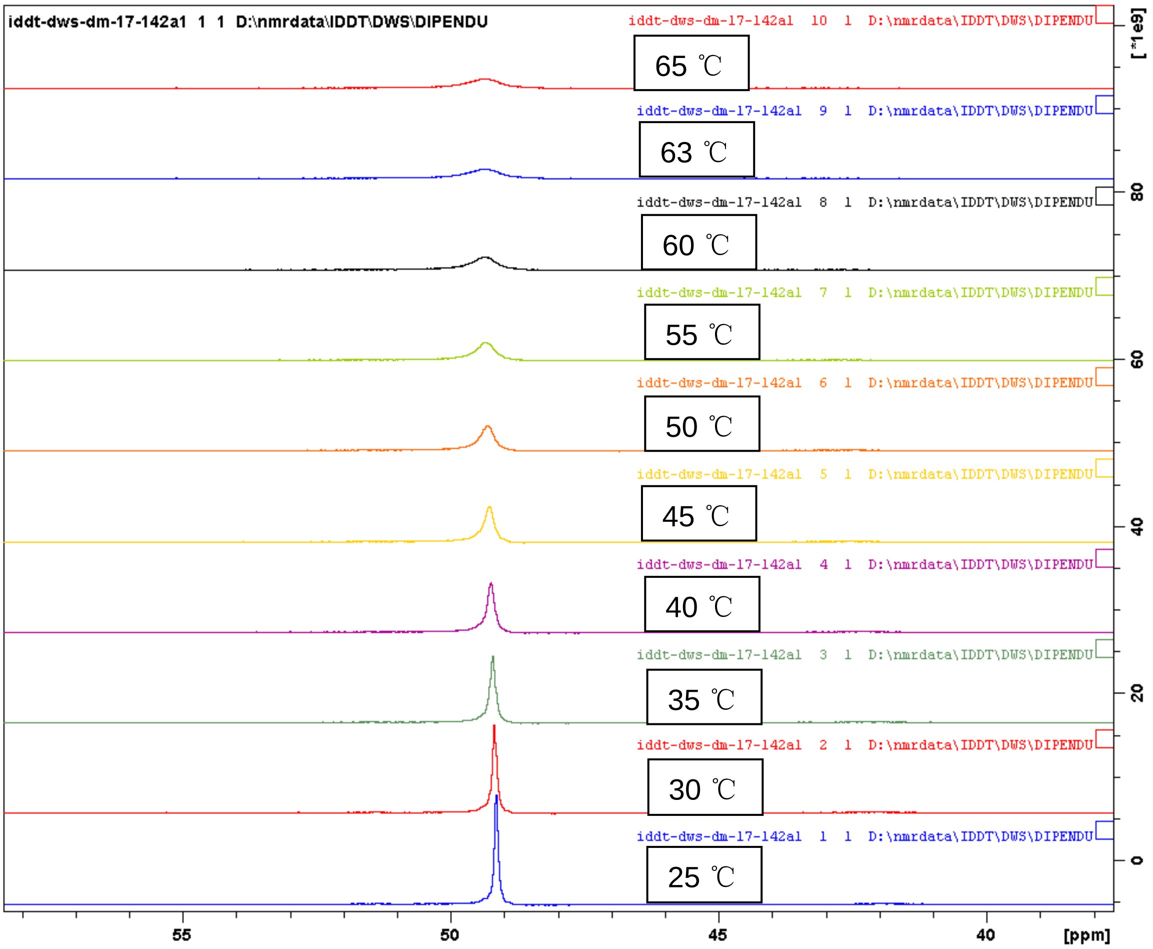

Figure S20.^{31}P NMR (203 MHz) spectra for high temperature analysis for P(*o*-Tol)$_3$/B(C_6F_5)$_3$ with diethyl azodicarboxylate in $CDCl_3$.

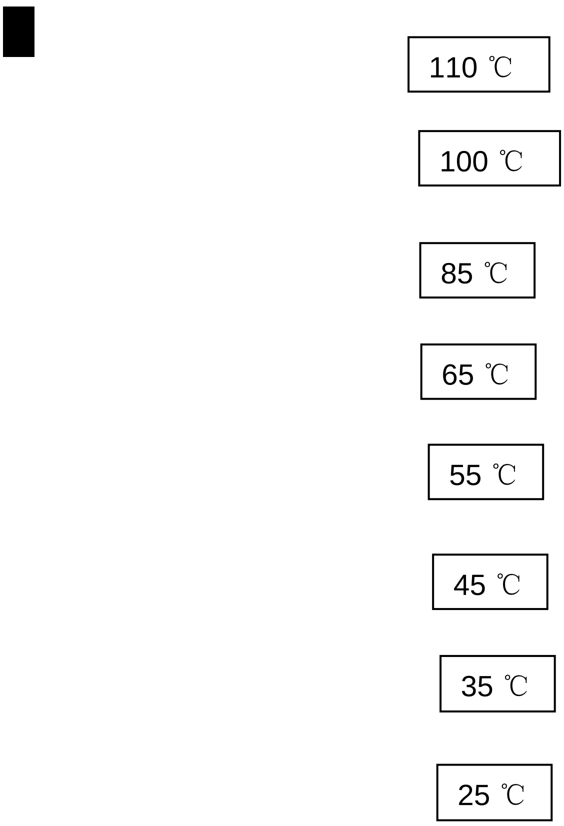

Figure S21.^{11}B NMR (161 MHz) spectra for high temperature analysis for P(*o*-Tol)$_3$/B(C_6F_5)$_3$ with diethyl azodicarboxylate in d_8-Toluene.

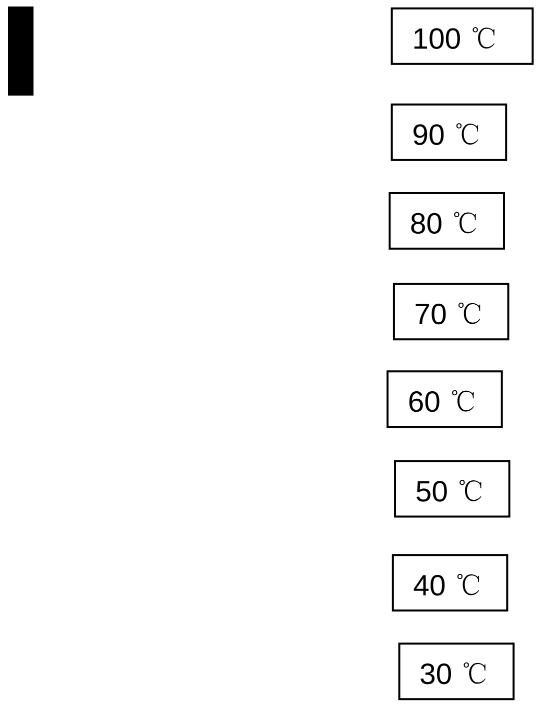

Figure S22. ^{31}P NMR (203 MHz) spectra for variable high temperature study for compound **1** in d_8-toluene.

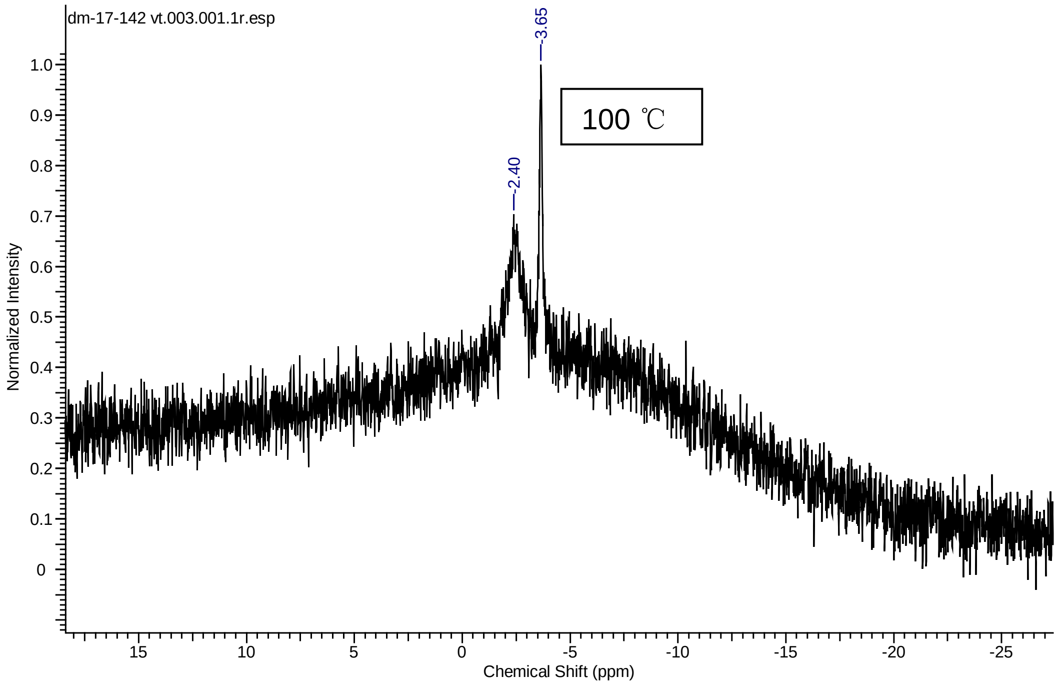

Figure S23. ^{11}B NMR (161 MHz) spectrum at 100 ℃ (probe temperature) for compound **1** in d_8-toluene.

Control reactions

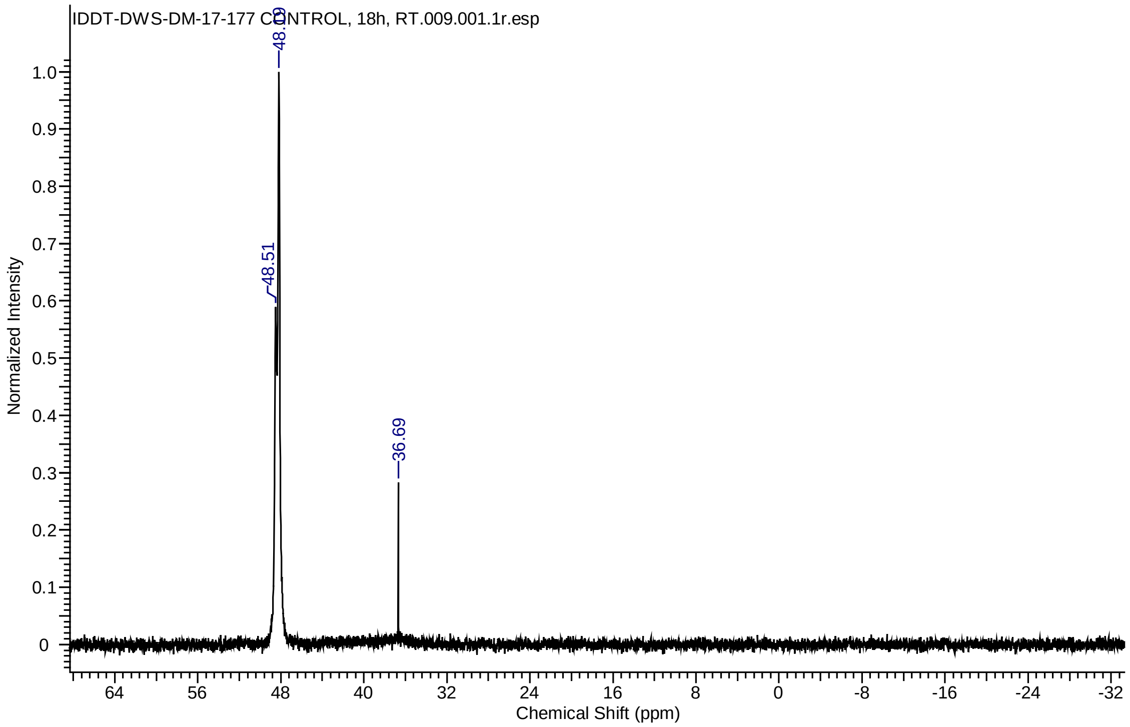

Figure S24.^{31}P NMR (203 MHz) spectrum of the crude mixture of diethyl azodicarboxylate and P(*o*-Tol)$_3$ in DCM after 24 h at RT.

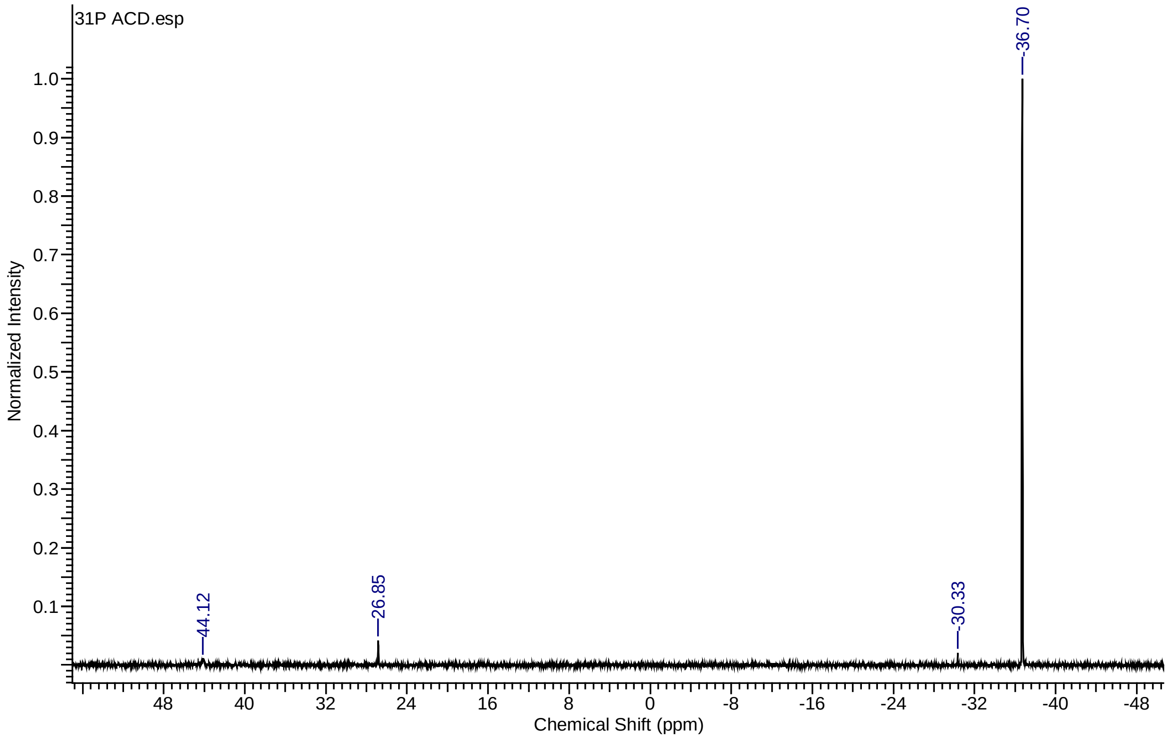

Figure S25.^{31}P NMR (203 MHz) spectrum of the crude mixture of diethyl azodicarboxylate and P(Mes)$_3$ in DCM after 24 h at RT.

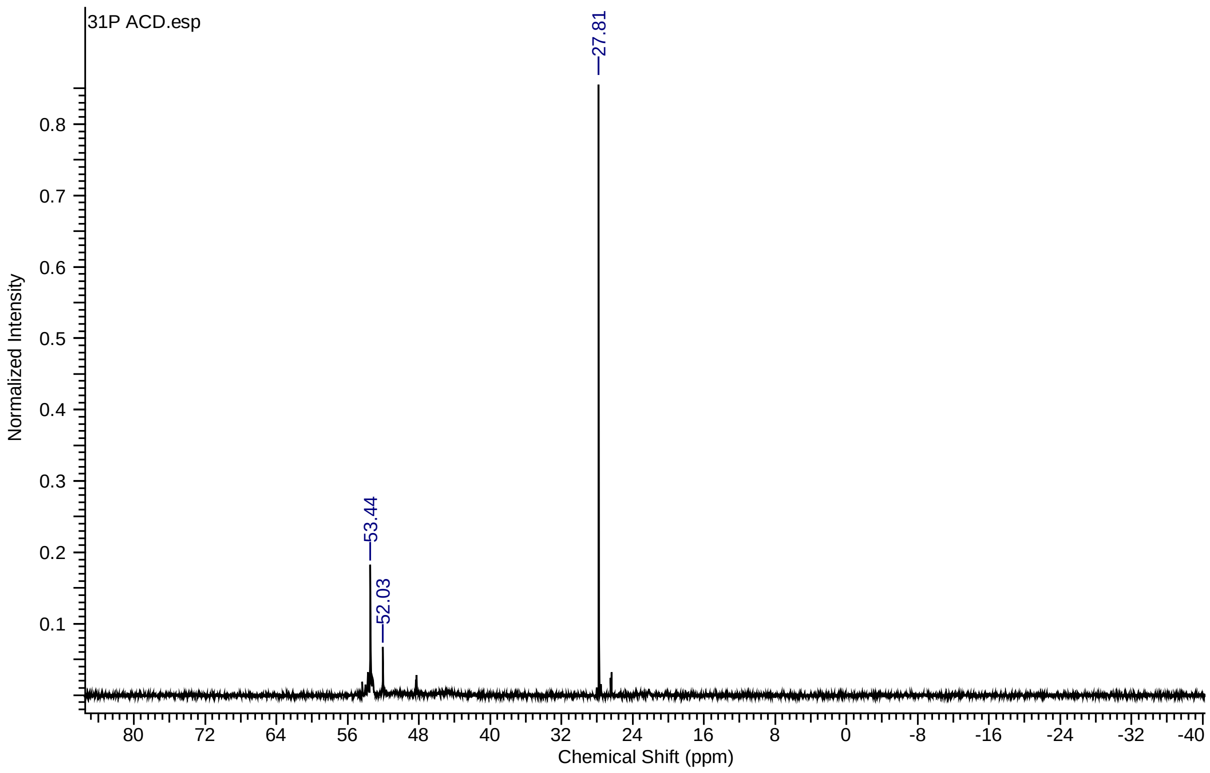

Figure S26.^{31}P NMR (203 MHz) spectrum of the crude mixture of diethyl azodicarboxylate and PPh_3 in DCM after 24 h at RT.

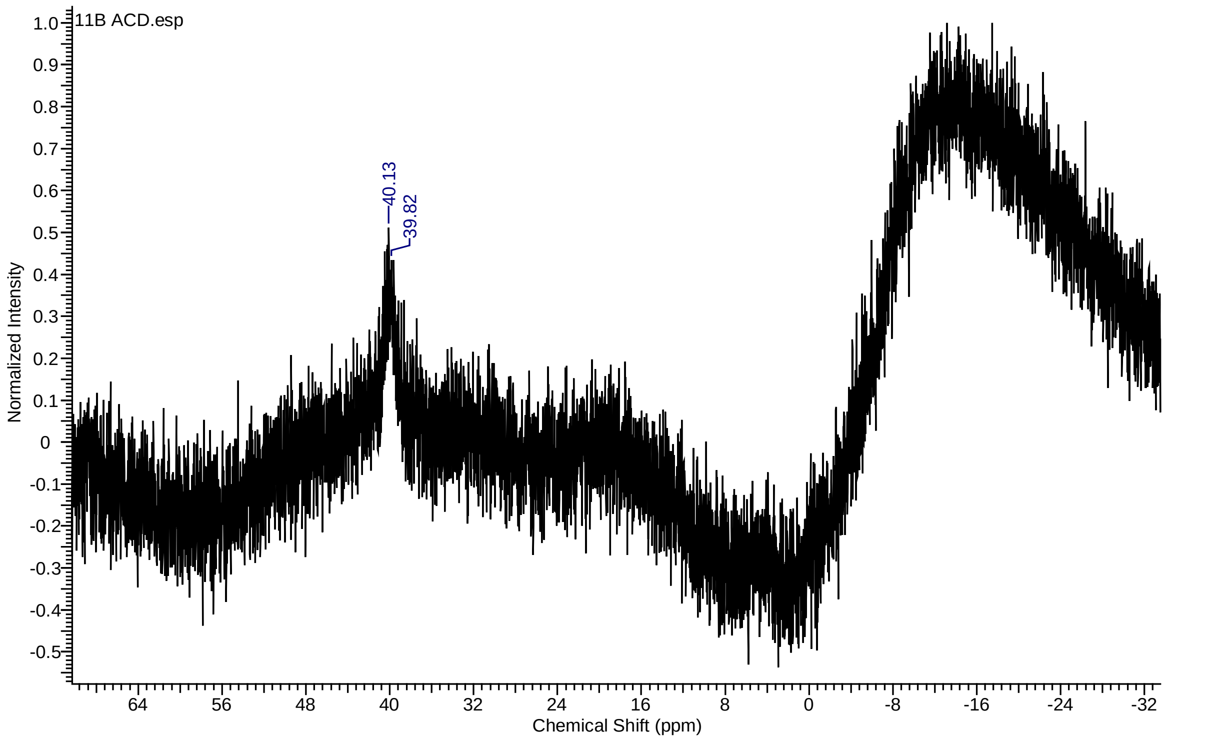

Figure S27.^{11}B NMR (161 MHz) spectrum of the crude mixture of diethyl azodicarboxylate and $B(C_6F_5)_3$ in DCM after 24 h at RT.

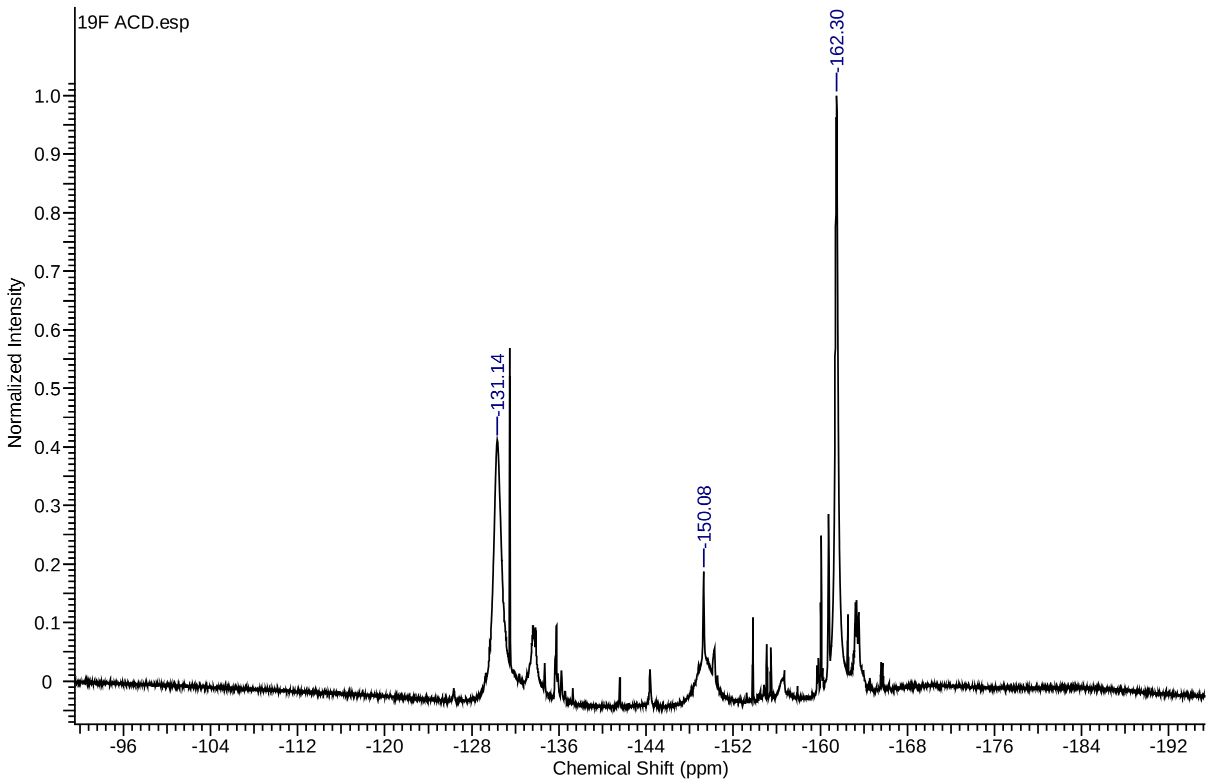

Figure S28.^{19}F NMR (471 MHz) spectrum of the crude mixture of diethyl azodicarboxylate and $B(C_6F_5)_3$ in DCM after 24 h at RT.

Experimental References

1. Sheldrick, G. M. *Acta Cryst*. *Sec*. *A* **2008**, 64, 112-122.

2. Dolomanov, O. V.; Bourhis, L. J.; Gildea, R. J.; Howard, J. A. K.; Puschmann, H. *J*. *Appl*. *Crystallogr*. **2009**, *42*, 339-341.

3. Gazis, T. A.; Dasgupta, A.; Hill, M. S.; Rawson, J. M.; Wirth, T.; Melen, R. L., *Dalton Trans*., **2019**, 48, 12391-12395.

4. Liu, Q.; Yang, L.; Yao, C.; Geng, J.; Wu, Y.; Hu, X. *Org*. *Lett*., **2021**, 23, 9, 3685–3690.

Computational Details

The quantum chemical DFT calculations have been performed with the TURBOMOLE 7.4 suite of programs[1] Initial structures generated according to conceivable Lewis structures are checked with the CREST method using the xTB program for low-lying conformers as input.[2] The structures are fully optimized at the TPSS-D3/def2-TZVP + COSMO level of theory, which combines the TPSS meta-GGA density functional[3] with the BJ-damped DFT-D3 dispersion correction[4] and the def2-TZVP basis set,[5] using the Conductor-like Screening Model (COSMO) continuum solvation model[6] for CH_2Cl_2 solvent (dielectric constant $\varepsilon = 8.93$ and solvent diameter $R_{solv} = 2.94$ Å). The density-fitting RI-J approach[5a, 7] is used to accelerate the geometry optimization and numerical harmonic frequency calculations[8] in solution. The optimized structures are characterized by frequency analysis to identify the nature of located stationary points (no imaginary frequency for true minima and only one imaginary frequency for transition state) and to provide thermal corrections (at 298.15 K and 1 atm) according to the modified ideal gas–rigid rotor–harmonic oscillator model.[9] This choice of dispersion-corrected meta-GGA functional makes the efficient exploration of all potential reaction paths possible.

The final solvation free energies in CH_2Cl_2 solution are computed with the COSMO-RS solvation model[10] (parameter file: BP_TZVP_C30_1601.ctd) using the COSMOtherm program package[[11] on the above TPSS-D3 optimized structures, and corrected by +1.89 kcal·mol^{-1} to account for higher reference solute concentration of 1 mol·L^{-1} usually used in solution. To check the effects of the chosen DFT functional on the reaction energies and barriers, single-point calculations at the meta-GGA TPSS-D3[3] and hybrid-meta-GGA PW6B95-D3[12] levels are performed using a larger def2-QZVP basis set.[5b, 13] The final reaction Gibbs free energies (ΔG) are determined from the electronic single-point energies plus TPSS-D3 thermal corrections and COSMO-RS solvation free energies. In our discussion, higher-level PW6B95-D3 Gibbs free energies (in kcal/mol, at 298.15 K and 1 mol/L concentration) will be used in our discussion unless specified otherwise. The applied DFT methods in combination with the large AO basis set provide usually accurate electronic energies leading to errors for chemical energies (including barriers) on the order of typically 1-2 kcal/mol. This has been tested thoroughly for the huge data base GMTKN55[14] which is the common standard in the field of DFT benchmarking. Gibbs energies in toluene are computed using the same TPSS-D3 optimized structures and thermal corrections, and COSMO-RS solvation free energies computed with the same parameter file for toluene instead of CH_2Cl_2.

To help experimental NMR assignment, nuclear magnetic shielding constants are computed using the GIAO (Gauge Including Atomic Orbital) method[15] at the TPSS/def2-QZVP level; final ^{31}P, ^{11}B NMR chemical shifts are computed using the known experimental ^{31}P and ^{11}B-NMR signal of compound **1** (o-Tol)$_3$PN(CO_2Et)N=C(OEt)OB(C_6F_5)$_3$ in this work as reliable referece, while ^{13}C and ^{1}H NMR chemical shifts are computed relative to Si(CH_3)$_4$.

References

[1] *TURBOMOLE V7.4*, **2019**, a development of University of Karlsruhe and Forschungszentrum Karlsruhe GmbH, 1989-2007, TURBOMOLE GmbH, since 2007; available from http://www.turbomole.com.
[2] a) P. Pracht, F. Bohle, S. Grimme, *Phys. Chem. Chem. Phys.* **2020**, *22*, 7169-7192; b) S. Grimme, *J. Chem. Theory Comput.* **2019**, *15*, 2847-2862.
[3] J. Tao, J. P. Perdew, V. N. Staroverov, G. E. Scuseria, *Phys. Rev. Lett.* **2003**, *91*, 146401.
[4] a) S. Grimme, J. Antony, S. Ehrlich, H. Krieg, *J. Chem. Phys.* **2010**, *132*, 154104-154119; b) S. Grimme, S. Ehrlich, L. Goerigk, *J. Comput. Chem.* **2011**, *32*, 1456-1465.
[5] a) F. Weigend, M. Häser, H. Patzelt, R. Ahlrichs, *Chem. Phys. Lett.* **1998**, *294*, 143-152; b) F. Weigend, R. Ahlrichs, *Phys. Chem. Chem. Phys.* **2005**, *7*, 3297-3305.
[6] A. Klamt, G. Schüürmann, *J. Chem. Soc., Perkin Trans. 2* **1993**, 799-805.
[7] a) K. Eichkorn, F. Weigend, O. Treutler, R. Ahlrichs, *Theor. Chem. Acc.* **1997**, *97*, 119-124; b) F. Weigend, *Phys. Chem. Chem. Phys.* **2006**, *8*, 1057-1065.
[8] P. Deglmann, K. May, F. Furche, R. Ahlrichs, *Chem. Phys. Lett.* **2004**, *384*, 103-107.
[9] S. Grimme, *Chem. Eur. J.* **2012**, *18*, 9955-9964.
[10] F. Eckert, A. Klamt, *AIChE J.* **2002**, *48*, 369-385.
[11] Eckert, F.; Klamt, A. *COSMOtherm, Version C3.0, Release 16.01; COSMOlogic GmbH & Co. KG, Leverkusen, Germany* **2015**.
[12] Y. Zhao, D. G. Truhlar, *J. Phys. Chem. A* **2005**, *109*, 5656-5667.
[13] F. Weigend, F. Furche, R. Ahlrichs, *J. Chem. Phys.* **2003**, *119*, 12753-12762.
[14] L. Goerigk, A. Hansen, C. Bauer, S. Ehrlich, A. Najibi, S. Grimme, *Phys. Chem. Chem. Phys.* **2017**, *19*, 32184-32215.
[15] G. Schreckenbach, T. Ziegler, *J. Phys. Chem.* **1995**, *99*, 606-611.

Table S1. TPSS-D3/def2-TZVP + COSMO computed imaginary frequency (ImF), zero-point energies (ZPE), gas-phase enthalpic (Hc) and Gibbs free-energy (Gc) corrections; the COSMO-RS computed solvation enthalpic (Hsol) and Gibbs free-energy (Gsol) corrections in CH_2Cl_2 solution; TPSS-D3/def2-QZVP and PW6B95-D3/def2-QZVP single-point energies (TPSS-D3 and PW6B95-D3); the total PW6B95-D3 free energies G_P; the relative electronic energies (ΔE_T and ΔE_P) and Gibbs energies (ΔG_T and ΔG_P) at the TPSS-D3 and PW6B95-D3 levels. (group Ph = C_6H_5; Mes = mesityl; To = *o*-$C_6H_5CH_3$; E = $CO_2CH_2CH_3$)

Reactions in CH_2Cl_2	Im cm^{-1}	ZPE kcal /mol	Hc kcal /mol	Gc kcal /mol	Hsol kcal /mol	Gsol kcal /mol	TPSS-D3 E_h	PW6B95-D3 E_h	G_P E_h	ΔE_T kcal /mol	ΔE_P kcal /mol	*ΔG_P kcal /mol*	ΔG_T kcal /mol
The Bf_3/PTo_3 pair is frustrated by 12.3 kcal/mol (borane $Bf_3 = B(C_6F_5)_3$)													
Bf_3 + PTo_3	0.0	315.02	347.13	253.03	-35.27	-26.89	-3364.48026	-3367.88906	-3367.52265	0.00	0.00	*0.00*	0.00
Bf_3PTo_3	0.0	316.92	348.85	271.41	-30.02	-24.45	-3364.48935	-3367.89961	-3367.50304	-5.70	-6.62	*12.30*	13.23
Addition of EN=NE (i.e., DEAD with E = $CO_2CH_2CH_3$) and PTo_3 is -11.3 kcal/mol exergonic and kinetically very facile													
PTo_3 + EN=NE	0.0	326.47	348.47	273.71	-37.03	-26.42	-1800.29359	-1802.09828	-1801.69817	0.00	0.00	*0.00*	0.00
**TSA**	46.3i	325.98	348.42	287.94	-37.30	-26.29	-1800.29962	-1802.09698	-1801.67701	-3.78	0.82	*13.28*	8.68
**Aa**	0.0	328.79	350.60	291.96	-42.65	-29.65	-1800.32853	-1802.13726	-1801.71624	-21.93	-24.46	*-11.33*	-8.80
Reversible EN=NE addition to Bf_3 is 0.5 kcal/mol endergonic over a moderate barrier of 18.8 kcal/mol													
EN=NE + Bf_3	0.0	200.45	227.76	142.86	-30.27	-22.39	-2855.05799	-2857.95724	-2857.75923	0.00	0.00	*0.00*	0.00
**TSB**	259.8i	199.64	227.48	156.32	-24.06	-18.99	-2855.05519	-2857.95111	-2857.72925	1.75	3.85	*18.82*	16.72
**Ba**	0.0	202.03	229.21	160.02	-24.75	-19.58	-2855.08701	-2857.98532	-2857.75849	-18.21	-17.62	*0.47*	-0.13
..EN=NE adduct of less Lewis acidic BPh_3 is even less stable													
BPh_3 + EN=NE	0.0	277.69	296.25	228.94	-35.44	-25.18	-1365.74007	-1367.18740	-1366.85665	0.00	0.00	*0.00*	0.00
**Bc**	0.0	278.74	297.41	244.64	-31.05	-23.08	-1365.75487	-1367.19549	-1366.83941	-9.29	-5.08	*10.82*	6.61
*..further Bf_3 addition to **Aa** is also exergonic by -9.3, -19.6, and -22.2 kcal/mol to form isomers **III**, **II**, and **I**, respectively.*													
**Aa** + Bf_3	0.0	423.29	463.81	353.05	-56.91	-41.08	-4009.95086	-4014.01127	-4013.50809	0.00	0.00	*0.00*	0.00
**1_I**	0.0	424.00	464.93	369.72	-40.54	-32.80	-4010.01683	-4014.08340	-4013.54348	-41.40	-45.26	*-22.20*	-18.34
**1_II**	0.0	424.14	465.05	369.60	-40.94	-33.14	-4010.01239	-4014.07846	-4013.53926	-38.61	-42.16	*-19.56*	-16.01
**1_IIa**	0.0	423.90	464.97	369.41	-42.99	-35.09	-4010.00829	-4014.07448	-4013.53870	-36.04	-39.66	*-19.21*	-15.58
**1_III**	0.0	424.73	465.14	371.72	-41.34	-33.30	-4009.99770	-4014.06523	-4013.52291	-29.39	-33.86	*-9.30*	-4.83
Addition of EN=NE to $PMes_3$ is -2.6 kcal/mol exergonic													
$PMes_3$ +EN=NE	0.0	428.95	456.87	371.59	-39.69	-28.54	-2036.33954	-2038.39246	-2037.83975	0.00	0.00	*0.00*	0.00
**Ab**	0.0	431.06	458.98	389.09	-42.18	-29.71	-2036.36470	-2038.41956	-2037.84384	-15.79	-17.00	*-2.57*	-1.35
*..further Bf_3 addition to **Ab** is also exergonic by -18.7 and -17.0 kcal/mol to form isomers **I** and **II**, respectively.*													

Bf_3 + **Ab**	0.0	525.56	572.18	450.18	-56.43	-41.13	-4245.98703	-4250.29357	-4249.63569	0.00	0.00	*0.00*	0.00
**2_I**	0.0	527.31	573.96	468.15	-45.18	-36.77	-4246.04203	-4250.35597	-4249.66552	-34.51	-39.16	*-18.72*	-14.07
**2_II**	2.1	526.95	573.64	467.24	-44.20	-35.81	-4246.03852	-4250.35338	-4249.66284	-32.31	-37.53	*-17.03*	-11.82
**2_III**	0.0	527.62	573.91	469.77	-46.40	-37.49	-4246.00252	-4250.31579	-4249.62389	-9.72	-13.94	*7.41*	11.62
Addition of EN=NE to less bulky PPh_3 is -16.6 kcal/mol exergonic thus is more stable													
EN=NE + PPh_3	0.0	275.04	294.27	225.25	-37.15	-26.27	-1682.25750	-1683.93771	-1683.61460	0.00	0.00	*0.00*	0.00
**Ac**	0.0	276.72	296.06	241.74	-48.60	-34.02	-1682.29288	-1683.97502	-1683.64097	-22.20	-23.41	*-16.55*	-15.34
..further Bf_3 addition is also exergonic by -17.0, -15.2, and -17.8 kcal/mol to form isomers of type III, II, and I, respectively.													
Bf_3 + **Ac**	0.0	371.22	409.27	302.83	-62.86	-45.44	-3891.91521	-3895.84903	-3895.43283	0.00	0.00	*0.00*	0.00
**3_I**	0.0	372.69	410.79	320.55	-42.89	-34.99	-3891.97872	-3895.91925	-3895.46117	-39.86	-44.06	*-17.78*	-13.58
**3_II**	0.0	372.19	410.47	319.78	-40.08	-32.36	-3891.97739	-3895.91815	-3895.45711	-39.02	-43.38	*-15.23*	-10.88
**3_III**	0.0	372.61	410.61	320.95	-40.78	-32.91	-3891.97838	-3895.92200	-3895.45998	-39.64	-45.79	*-17.04*	-10.88
..while further BPh_3 addition is also exergonic by -15.2, -17.0, and -17.8 kcal/mol to form isomers of type III, II, and I, respectively.													
BPh_3 + **Ac**	0.0	448.46	477.76	388.92	-68.02	-48.23	-2402.59730	-2405.07919	-2404.53025	0.00	0.00	*0.00*	0.00
**4_I**	0.0	449.29	478.63	405.41	-50.72	-38.83	-2402.63455	-2405.11786	-2404.53067	-23.38	-24.26	*-0.26*	0.62
**4_II**	0.0	449.68	478.96	406.01	-50.68	-38.81	-2402.64361	-2405.12756	-2404.53937	-29.06	-30.35	*-5.72*	-4.43
**4_III**	0.0	450.19	479.45	406.55	-49.68	-38.09	-2402.65066	-2405.13153	-2404.54134	-33.49	-32.84	*-6.96*	-7.61
*Addition of EN=NE to the bulky phosphine **jp** $PMes_2(C_6H_4CH=CH_2)$ is -5.0 kcal/mol exergonic*													
EN=NE + **jp**	0.0	398.65	424.95	341.95	-41.51	-29.63	-1995.76361	-1997.77618	-1997.27244	0.00	0.00	*0.00*	0.00
**Ad**	0.0	401.73	427.29	362.18	-46.79	-32.76	-1995.79429	-1997.80845	-1997.28048	-19.25	-20.25	*-5.04*	-4.04
*..while **jb** $BPh_2C_6H_4CH=CH_2$ addition to **Ad** is exergonic by -5.3 and -4.7 kcal/mol to form type I and II but 9.4 kcal/mol endergonic to III.*													
**jb** + **Ad**	0.0	593.95	630.92	527.28	-68.26	-48.45	-2793.55143	-2796.44918	-2795.68009	0.00	0.00	*0.00*	0.00
**5_I**	0.0	593.48	631.44	542.40	-60.12	-46.72	-2793.58277	-2796.48139	-2795.68847	-19.67	-20.22	*-5.26*	-4.71
**5_II**	0.0	593.55	631.42	542.68	-52.63	-40.61	-2793.59203	-2796.49063	-2795.68753	-25.48	-26.01	*-4.67*	-4.14
**5_III**	0.0	594.40	631.81	544.37	-59.71	-45.36	-2793.56711	-2796.46339	-2795.66515	-9.84	-8.92	*9.38*	8.45
*Similar BPh_3 addition to **Ad** is exergonic by -3.5 and -2.5 kcal/mol to form type I and II but 4.7 kcal/mol endergonic to III.*													
**Aa** + BPh_3	0.0	500.52	532.30	439.13	-62.07	-43.87	-2520.63295	-2523.24143	-2522.60551	0.00	0.00	*0.00*	0.00
**6_I**	0.0	501.08	533.07	455.43	-49.38	-38.18	-2520.67010	-2523.27900	-2522.61106	-23.32	-23.58	*-3.48*	-3.22
**6_II**	0.0	501.48	533.39	455.75	-49.13	-38.11	-2520.66955	-2523.27800	-2522.60943	-22.96	-22.94	*-2.46*	-2.48
**6_III**	0.0	501.51	533.28	456.13	-50.96	-38.86	-2520.66053	-2523.26598	-2522.59800	-17.31	-15.40	*4.71*	2.81

Table S2. TPSS-D3/def2-TZVP + COSMO optimized Cartesian coordinates (in Å) in CH_2Cl_2 solution. Each structure is labeled by a specific name (See **Table S1**), followed by the number of atoms, the total energy (in hartrees), and the detailed atomic coordinates (in double-column text list).

1_IIa : type IIa isomer of **1**
99
Energy = -4009.824111019

P	-3.3679421	0.4352653	-0.0251653
C	-4.6040855	-0.7686377	-0.5391718
C	-2.8308107	1.3506970	-1.4802449
C	-4.0651136	1.5706337	1.1913418
C	-5.8165591	-0.7989455	0.1683032
C	-4.4168692	-1.5831228	-1.6809538
C	-1.4517466	1.4534571	-1.7124384
C	-3.7617704	1.9034793	-2.3928599
C	-4.3528352	1.1468154	2.5107322
C	-4.2718013	2.9037921	0.8098618
C	-6.8415555	-1.6559390	-0.2168799
H	-5.9646708	-0.1339210	1.0120703
C	-5.4649891	-2.4352894	-2.0427933
C	-3.1681559	-1.5680737	-2.5215321
C	-0.9740463	2.1121066	-2.8394055
H	-0.7524919	0.9947188	-1.0253979
C	-3.2474147	2.5651887	-3.5144555
C	-5.2601620	1.7831931	-2.2533863
C	-4.8866489	2.0937564	3.3903522
C	-4.0404126	-0.2345371	3.0236532
C	-4.7972901	3.8259532	1.7097487
H	-4.0008713	3.2255731	-0.1899352
C	-6.6598422	-2.4817127	-1.3255692
H	-7.7713836	-1.6736347	0.3424413
H	-5.3336148	-3.0780154	-2.9092093
H	-3.2292860	-0.7896686	-3.2912496
H	-2.2720490	-1.3855726	-1.9283609
H	-3.0485675	-2.5283409	-3.0288598
C	-1.8763592	2.6724960	-3.7421350
H	0.0960339	2.1870655	-2.9999843
H	-3.9460066	3.0011763	-4.2236822
H	-5.7508704	2.5890990	-2.8049032
H	-5.6103203	0.8307004	-2.6678090
H	-5.5961660	1.8269918	-1.2144762
C	-5.1133507	3.4143510	3.0029150
H	-5.1179326	1.7843974	4.4062107
H	-4.5695540	-0.4190516	3.9614302
H	-4.3186999	-1.0227462	2.3173596
H	-2.9632859	-0.3205943	3.2086249
H	-4.9505902	4.8553071	1.4022078
H	-7.4494732	-3.1589201	-1.6374045
H	-1.5175089	3.1925450	-4.6254880
H	-5.5262420	4.1216450	3.7161575
C	-0.0777491	0.8268980	1.3408887
O	0.5712741	0.1048197	0.4842977
O	0.6186382	1.6926494	2.0785902
C	-1.7874791	-1.5846665	1.1164565
O	-1.0760353	-1.8891877	2.0467614

O	-2.4594588	-2.4220991	0.3132855
N	-2.0039773	-0.2460456	0.7204108
N	-1.3783907	0.7682385	1.5202274
C	-0.1255405	2.5996674	2.9556207
C	-0.7097641	3.7638915	2.1758628
H	0.6320180	2.9282910	3.6692220
H	-0.8989919	2.0233412	3.4673780
H	-1.4540098	3.4107674	1.4592835
H	-1.1998397	4.4517169	2.8736317
H	0.0753498	4.3097314	1.6442774
C	-2.3927308	-3.8429451	0.6627697
C	-3.4911933	-4.1969215	1.6476201
H	-2.5209640	-4.3491456	-0.2950302
H	-1.3969545	-4.0444306	1.0616711
H	-3.3450195	-3.6718046	2.5958418
H	-4.4732592	-3.9375592	1.2406713
H	-3.4696606	-5.2742697	1.8430686
B	2.0510421	-0.0868223	0.0863151
C	2.9563241	1.2066569	0.5150016
C	2.5142823	-1.4989658	0.7885919
C	1.9171657	-0.2595454	-1.5540066
C	2.5539105	2.4912510	0.1427445
C	4.1317454	1.1700046	1.2611665
C	3.1761133	-2.5361389	0.1322975
C	2.2080796	-1.7705770	2.1239278
C	2.6629569	0.4150107	-2.5207937
C	0.9664236	-1.1401779	-2.0822672
C	3.2219721	3.6538752	0.4960867
C	4.8429884	2.3100855	1.6378562
C	3.4641981	-3.7663792	0.7190725
C	2.4669085	-2.9841250	2.7503374
C	2.4552981	0.2848737	-3.8935543
C	0.7280161	-1.3071462	-3.4424373
C	4.3836367	3.5634488	1.2579958
C	3.0981586	-3.9979252	2.0386834
C	1.4734218	-0.5780064	-4.3614981
F	1.4371802	2.6498748	-0.6144288
F	4.6712428	-0.0071553	1.6705079
F	3.6050367	-2.3911748	-1.1505423
F	1.6557363	-0.8094861	2.9001086
F	3.6776876	1.2478553	-2.1732051
F	0.2276690	-1.9245828	-1.2590982
F	2.7636918	4.8662552	0.1138138
F	5.9751360	2.2058449	2.3669721
F	4.1008001	-4.7331679	0.0216062
F	2.1317108	-3.1837939	4.0430898
F	3.2026572	0.9862445	-4.7728534
F	-0.2065164	-2.1762855	-3.8833420
F	5.0544796	4.6755460	1.6128837
F	3.3659787	-5.1808053	2.6253958
F	1.2558680	-0.7172376	-5.6825133

1_III : type III isomer of **1**

99

Energy = -4009.813985725

P	-2.6555395	-0.4113566	0.2542010
C	-2.1901702	-2.1373329	0.0387069
C	-3.2308371	0.2355047	-1.3393591
C	-3.9251549	-0.4504220	1.5466649
C	-2.3485064	-2.7163442	-1.2350579
C	-1.9280875	-2.9574007	1.1617489
C	-2.2344130	0.1991375	-2.3314386
C	-4.4991090	0.7892160	-1.6315884
C	-5.0195066	-1.3417521	1.4309112
C	-3.7793081	0.3528619	2.6862417
C	-2.2363280	-4.0880008	-1.4122060
H	-2.5979137	-2.0968968	-2.0868720
C	-1.8036951	-4.3351801	0.9450625
C	-1.8167724	-2.4550301	2.5734428
C	-2.4781433	0.6507655	-3.6209959
H	-1.2477084	-0.1694151	-2.0749295
C	-4.7190210	1.2223050	-2.9477077
C	-5.6164002	1.0076206	-0.6429751
C	-5.9467765	-1.3651727	2.4790426
C	-5.2529929	-2.2627442	0.2586268
C	-4.7155749	0.3017426	3.7126427
H	-2.9047878	0.9788724	2.7967454
C	-1.9569924	-4.9021471	-0.3152955
H	-2.3661964	-4.5148308	-2.4008088
H	-1.5876796	-4.9715960	1.7990832
H	-1.0441228	-3.0185204	3.1031639
H	-1.5691315	-1.3967343	2.6420556
H	-2.7652124	-2.6116342	3.1022485
C	-3.7402816	1.1522650	-3.9349514
H	-1.6887774	0.6208149	-4.3650569
H	-5.6874399	1.6536332	-3.1868883
H	-6.2686518	1.8036734	-1.0107399
H	-6.2322439	0.1100585	-0.5243737
H	-5.2475375	1.2842767	0.3439325
C	-5.8078762	-0.5581823	3.6058250
H	-6.7992546	-2.0344503	2.3976812
H	-4.6997678	-3.2003168	0.3834743
H	-6.3160834	-2.5070147	0.1885460
H	-4.9388513	-1.8270895	-0.6933311
H	-4.5859383	0.9265130	4.5906326
H	-1.8644564	-5.9768896	-0.4405136
H	-3.9559307	1.5106537	-4.9369910
H	-6.5492391	-0.6047823	4.3980977
C	0.1405841	0.4569170	2.4436078
O	-0.7796156	0.5132429	3.2517112
O	1.4260645	0.6697137	2.7492572
C	-1.5894923	1.9661212	0.9144934
O	-0.8189833	2.7486316	1.4183562
O	-2.8529868	2.2200666	0.5427949
N	-1.2980919	0.5972504	0.6525102
N	-0.0097159	0.1638836	1.0812721
C	1.7501498	0.8976561	4.1555613
C	3.0486152	0.1825678	4.4722188
H	0.9163690	0.5389059	4.7600690
H	1.8411516	1.9811207	4.2768632
H	3.8464205	0.5013481	3.7966746
H	3.3443513	0.4217744	5.4992430
H	2.9272214	-0.9010618	4.3924932
C	-3.3373731	3.6008208	0.7041387
C	-3.1702846	4.3610087	-0.5958953
H	-4.3844846	3.4775457	0.9831860
H	-2.7815212	4.0506675	1.5275871
H	-3.6984706	3.8583344	-1.4107801
H	-3.5891023	5.3656331	-0.4749457
H	-2.1146300	4.4527705	-0.8607190
B	1.2798608	0.2377032	0.0881840
C	1.0196028	-0.4653380	-1.4030598
C	1.7230731	1.8314346	-0.0004948
C	2.5154501	-0.7379324	0.6186989
C	1.3047300	0.0907126	-2.6597420
C	0.7589919	-1.8449765	-1.4669545
C	0.9447070	2.7544290	-0.7024782
C	2.8049247	2.4162516	0.6625903
C	3.7351977	-0.6708545	-0.0630555
C	2.4698352	-1.7539657	1.5751169
C	1.2277226	-0.6128134	-3.8619734
C	0.7131721	-2.5938680	-2.6391737
C	1.1999038	4.1153590	-0.7800964
C	3.0955237	3.7802255	0.6291582
C	4.8351731	-1.4789929	0.1988100
C	3.5446378	-2.5892575	1.8724757
C	0.9194566	-1.9648908	-3.8601581
C	2.2906374	4.6424740	-0.1001715
C	4.7434330	-2.4523520	1.1862781
F	1.7280098	1.3663551	-2.8090891
F	0.6031856	-2.5544282	-0.3293559
F	-0.1733637	2.3377227	-1.3604189
F	3.6773030	1.6830274	1.3984978
F	3.9006791	0.2366439	-1.0622067
F	1.3441553	-2.0134458	2.2860940
F	1.4792508	0.0104749	-5.0323601
F	0.5060101	-3.9245645	-2.6062883
F	0.3891221	4.9353435	-1.4870883
F	4.1625295	4.2695814	1.3001186
F	5.9847869	-1.3301046	-0.4950901
F	3.4263062	-3.5409473	2.8257738
F	0.8595695	-2.6589311	-5.0092970
F	2.5561931	5.9624689	-0.1444472
F	5.7913154	-3.2522877	1.4641294

1_II : type II isomer of **1**

99

Energy = -4009.826675563

P	-3.3444828	0.4501939	0.0023852
C	-4.6112260	-0.7154760	-0.5188584
C	-2.7877101	1.3451183	-1.4562712

C	-4.0038021	1.6028538	1.2213592
C	-5.8457194	-0.6784649	0.1488950
C	-4.4313886	-1.5333453	-1.6585680
C	-1.4096510	1.3902277	-1.7097067
C	-3.7103442	1.9214122	-2.3629131
C	-4.3180326	1.1797367	2.5341624
C	-4.1563739	2.9458751	0.8483962
C	-6.9079104	-1.4645839	-0.2837957
H	-5.9813017	-0.0145049	0.9958436
C	-5.5180703	-2.3104379	-2.0706079
C	-3.1486569	-1.5954558	-2.4430991
C	-0.9248834	2.0151088	-2.8526340
H	-0.7187805	0.9156727	-1.0244869
C	-3.1883100	2.5460147	-3.5019138
C	-5.2102002	1.8679179	-2.1994939
C	-4.8219173	2.1390140	3.4181186
C	-4.0654586	-0.2179510	3.0364499
C	-4.6531134	3.8799963	1.7519569
H	-3.8676925	3.2636918	-0.1478216
C	-6.7401135	-2.2842126	-1.3993715
H	-7.8563183	-1.4306526	0.2427242
H	-5.3951612	-2.9540486	-2.9376714
H	-3.1400296	-0.8356544	-3.2337040
H	-2.2738481	-1.4399268	-1.8108929
H	-3.0501733	-2.5740254	-2.9189645
C	-1.8181425	2.5956347	-3.7513245
H	0.1441263	2.0492283	-3.0299707
H	-3.8804011	2.9991177	-4.2068064
H	-5.6700070	2.7159017	-2.7136341
H	-5.6117791	0.9486366	-2.6415016
H	-5.5272072	1.8908254	-1.1545478
C	-4.9940098	3.4702897	3.0396765
H	-5.0744726	1.8305365	4.4291898
H	-4.6656210	-0.4148940	3.9279350
H	-4.2980253	-0.9880397	2.2944510
H	-3.0065292	-0.3196456	3.3028834
H	-4.7652286	4.9168573	1.4520383
H	-7.5607307	-2.9030136	-1.7502532
H	-1.4520097	3.0881717	-4.6473692
H	-5.3853842	4.1872816	3.7553633
C	-0.0362842	0.7565811	1.3957124
O	0.6215755	0.0234354	0.5539905
O	0.6633071	1.6063154	2.1498191
C	-1.9141404	-1.6416862	1.0388919
O	-2.5521575	-2.4885419	0.4381894
O	-1.0719806	-1.8476946	2.0531758
N	-1.9933464	-0.2681805	0.7435407
N	-1.3373700	0.7082012	1.5690566
C	-0.0755530	2.5000725	3.0458503
C	-0.6458384	3.6894475	2.2948748
H	0.6827685	2.8026572	3.7700912
H	-0.8564147	1.9183970	3.5397268
H	-1.3823145	3.3614261	1.5588977
H	-1.1424802	4.3569183	3.0075773
H	0.1466166	4.2496157	1.7903567
C	-0.8233044	-3.2435275	2.4255987
C	-0.7235626	-3.3028865	3.9354028
H	-1.6367306	-3.8548574	2.0327940
H	0.1120706	-3.5272686	1.9350656
H	0.0517505	-2.6275346	4.3047660
H	-1.6786554	-3.0339576	4.3965962
H	-0.4680996	-4.3237730	4.2375359
B	2.0936920	-0.0701343	0.0988008
C	2.9278641	1.2970921	0.4448349
C	2.7269234	-1.4134207	0.8093874
C	1.8980008	-0.3137642	-1.5252338
C	2.4107292	2.5420705	0.0801141
C	4.1550356	1.3631833	1.1019957
C	3.5124812	-2.3547214	0.1421776
C	2.5224462	-1.6998814	2.1609235
C	2.5337695	0.3810107	-2.5537726
C	1.0052823	-1.2963328	-1.9678528
C	3.0171012	3.7573781	0.3597218
C	4.8084528	2.5593782	1.4017895
C	4.0237394	-3.5055470	0.7380755
C	3.0176039	-2.8316154	2.8014870
C	2.2791503	0.1671750	-3.9079935
C	0.7220530	-1.5470413	-3.3062632
C	4.2348711	3.7681971	1.0336372
C	3.7704684	-3.7519215	2.0812377
C	1.3588172	-0.7996242	-4.2904673
F	1.2351331	2.6048028	-0.5969764
F	4.8100142	0.2395457	1.4932060
F	3.8606432	-2.1768402	-1.1592762
F	1.8364140	-0.8335173	2.9458634
F	3.4791737	1.3193536	-2.2900291
F	0.3784721	-2.1036955	-1.0756182
F	2.4450222	4.9235875	-0.0115249
F	5.9957292	2.5516120	2.0446755
F	4.7711201	-4.3766862	0.0278830
F	2.7875874	-3.0412699	4.1160693
F	2.9181979	0.8914976	-4.8512760
F	-0.1492447	-2.5141164	-3.6628233
F	4.8481575	4.9323597	1.3161458
F	4.2574101	-4.8545593	2.6795350
F	1.0959869	-1.0182095	-5.5918229

1_I : type I isomer of **1**

99

Energy = -4009.831525276

P	-3.0162021	-0.3545189	0.1387551
C	-4.7114794	0.2507990	0.1498140
C	-3.0083562	-1.9107502	1.0462308
C	-2.4406936	-0.6124279	-1.5459706
C	-5.3275227	0.5082002	-1.0848160
C	-5.4535951	0.3097857	1.3519040
C	-2.1329089	-2.0452707	2.1322926
C	-3.9165111	-2.9469810	0.7206526
C	-2.2526028	0.4799193	-2.4246498
C	-2.1717968	-1.9227891	-1.9663315

C	-6.6743793	0.8480859	-1.1462356
H	-4.7552900	0.4195326	-2.0021240
C	-6.8057360	0.6526983	1.2586200
C	-4.8675246	0.0094452	2.7059470
C	-2.1176682	-3.2104409	2.8888486
H	-1.4812421	-1.2243894	2.3987688
C	-3.8694168	-4.1122337	1.4960578
C	-4.9572451	-2.8583454	-0.3690757
C	-1.8533899	0.1946923	-3.7333472
C	-2.3838190	1.9160708	-1.9908908
C	-1.7617062	-2.1743049	-3.2711187
H	-2.2690238	-2.7441543	-1.2650049
C	-7.4148095	0.9205132	0.0331790
H	-7.1388260	1.0478049	-2.1065222
H	-7.3895382	0.7139486	2.1733263
H	-4.9704363	-1.0561616	2.9430016
H	-3.8087475	0.2678713	2.7630859
H	-5.3982474	0.5767658	3.4749591
C	-2.9847167	-4.2526059	2.5626884
H	-1.4322353	-3.3026672	3.7256218
H	-4.5510933	-4.9227749	1.2520047
H	-5.1958689	-3.8593006	-0.7378471
H	-5.8804107	-2.4148984	0.0223945
H	-4.6384943	-2.2510053	-1.2182649
C	-1.6150692	-1.1106688	-4.1597137
H	-1.7063177	1.0215567	-4.4222989
H	-3.2511943	2.0926468	-1.3472719
H	-2.4668992	2.5710625	-2.8606372
H	-1.4893034	2.2076674	-1.4285417
H	-1.5568706	-3.1922145	-3.5866534
H	-8.4680548	1.1833326	0.0010930
H	-2.9784890	-5.1719818	3.1407152
H	-1.3013147	-1.2935818	-5.1831303
C	0.2433876	0.2745032	1.4937398
O	1.5040645	0.0024591	1.3617961
O	-0.1802147	0.4623040	2.7552319
C	-2.2177093	2.0447001	1.2114149
O	-3.3434050	2.4230205	1.4858347
O	-1.1085708	2.7931624	1.1911407
N	-1.9111578	0.7184608	0.8597805
N	-0.5675696	0.3595156	0.4692368
C	0.8225122	0.3859615	3.8241455
C	0.0745437	0.5570224	5.1286799
H	1.5560286	1.1777320	3.6547458
H	1.3245593	-0.5817048	3.7553665
H	-0.6635259	-0.2395133	5.2645194
H	0.7869931	0.5133463	5.9584985
H	-0.4388795	1.5221177	5.1635889
C	-1.2334629	4.1744836	1.6666752
C	-0.5413605	4.3025319	3.0095497
H	-0.7469318	4.7751251	0.8958985
H	-2.2931813	4.4278753	1.7151573
H	-1.0477618	3.6961612	3.7660809
H	0.5035360	3.9870111	2.9418018
H	-0.5678529	5.3493274	3.3295859

B	2.2842647	0.0617558	0.0500237
C	1.9453050	-1.1950705	-0.9505700
C	1.9912185	1.5648310	-0.5458390
C	3.8516103	-0.1708469	0.5177935
C	1.1359099	-2.2929550	-0.6589510
C	2.5995130	-1.2778971	-2.1814512
C	2.3013423	2.6700303	0.2508202
C	1.3561286	1.8869443	-1.7433853
C	4.9563637	0.5675837	0.0928091
C	4.1669478	-1.2390750	1.3631556
C	0.9438372	-3.3582680	-1.5376608
C	2.4331778	-2.3138399	-3.0913984
C	2.0291551	3.9874148	-0.0970016
C	1.0693503	3.1925738	-2.1391255
C	6.2657584	0.2967514	0.4863202
C	5.4569561	-1.5431817	1.7866855
C	1.5864269	-3.3682337	-2.7670804
C	1.4039031	4.2543398	-1.3094061
C	6.5208257	-0.7651845	1.3445750
F	0.4808890	-2.4024654	0.5208226
F	3.4592474	-0.2904599	-2.5446410
F	2.9087323	2.4917734	1.4543054
F	0.9491768	0.9253978	-2.6030716
F	4.8131674	1.6205717	-0.7512424
F	3.1902052	-2.0711802	1.8056600
F	0.1191515	-4.3786950	-1.2082371
F	3.0770617	-2.3100564	-4.2791444
F	2.3546718	5.0075740	0.7283072
F	0.4475053	3.4354096	-3.3146436
F	7.2900713	1.0576238	0.0437653
F	5.6884773	-2.5892137	2.6096411
F	1.3891975	-4.3807915	-3.6349772
F	1.1175955	5.5214416	-1.6669783
F	7.7789333	-1.0412059	1.7365560

2_III : type III isomer of **2**
117
Energy = -4245.804666230

P	2.3714494	-0.2599773	-0.0242369
C	1.9554824	-1.7736949	0.9061352
C	3.2104439	1.0379135	0.9581323
C	3.5186230	-0.8739542	-1.3285074
C	2.1858991	-1.9376032	2.3076472
C	1.5301262	-2.9083350	0.1501338
C	2.4596905	1.6937795	1.9699809
C	4.5525359	1.4606017	0.7205667
C	4.3859746	-1.9392164	-0.9540438
C	3.5694941	-0.3988298	-2.6665301
C	1.8417519	-3.1545287	2.9007268
C	2.8831840	-0.9846161	3.2550079
C	1.1998393	-4.0884390	0.8165777
C	1.4999551	-3.0169834	-1.3518937
C	3.0351957	2.7028759	2.7328134
C	1.0288771	1.3685025	2.2624062
C	5.0734951	2.4795529	1.5311666

C	5.5268105	0.9662366	-0.3267359
C	5.0944350	-2.6193515	-1.9480170
C	4.7206279	-2.3539068	0.4640966
C	4.2818991	-1.1341008	-3.6153360
C	3.0437127	0.9329475	-3.1320823
C	1.3092203	-4.2295216	2.1952812
H	2.0123200	-3.2595566	3.9691157
H	3.7156453	-0.4529419	2.7957514
H	2.2056341	-0.2370994	3.6740413
H	3.2760764	-1.5677519	4.0910362
H	0.8447403	-4.9235519	0.2174755
H	0.6941706	-3.6868508	-1.6534728
H	1.3762917	-2.0732781	-1.8699274
H	2.4436662	-3.4548495	-1.6972152
C	4.3519353	3.1155636	2.5374271
H	2.4284560	3.1760945	3.5016792
H	0.7844773	0.3284739	2.0577812
H	0.8003105	1.5752030	3.3113537
H	0.3667157	1.9776510	1.6483034
H	6.0987330	2.7910097	1.3480641
H	6.4408889	1.5586932	-0.2483316
H	5.7994385	-0.0815109	-0.1927893
H	5.1449777	1.0756196	-1.3419815
C	5.0115983	-2.2797778	-3.2952948
H	5.7432764	-3.4370665	-1.6412642
H	4.1243563	-3.2051893	0.8048653
H	5.7733583	-2.6517792	0.4895902
H	4.5894880	-1.5425535	1.1826288
H	4.2906832	-0.7684340	-4.6397211
H	1.9609524	1.0090719	-3.0794930
H	3.4811214	1.7406378	-2.5380920
H	3.3380689	1.0916571	-4.1721066
C	0.9163795	-5.5067004	2.8868224
C	4.9699793	4.1932907	3.3862819
C	5.7585084	-3.0518771	-4.3501648
H	1.6175340	-6.3137436	2.6417390
H	0.9058383	-5.3816614	3.9725775
H	-0.0776431	-5.8314472	2.5614527
H	5.2467981	3.7921522	4.3693477
H	5.8734971	4.5957227	2.9207601
H	4.2638523	5.0120225	3.5575417
H	6.7113329	-2.5598961	-4.5831351
H	5.9808905	-4.0678248	-4.0128815
H	5.1830184	-3.1053423	-5.2793336
O	-1.7207545	-0.8445913	-2.6263830
C	-0.6186677	-0.5780280	-2.1794071
O	0.5040286	-0.5874383	-2.9385980
N	-0.3945189	-0.2877285	-0.8347251
C	0.2941743	-0.8790755	-4.3532995
N	0.8294564	0.4487221	-0.5973649
C	-0.1924140	0.3456511	-5.1085964
H	-0.4137666	-1.7066711	-4.4290421
H	1.2793455	-1.2048274	-4.6934803
C	0.8449340	1.7212563	-1.2530074
H	0.5195185	1.1708529	-5.0197851
H	-0.3015028	0.0919450	-6.1688536
H	-1.1601170	0.6729607	-4.7225844
O	0.0409100	2.0478158	-2.0973392
O	1.8545804	2.4713658	-0.8023880
C	2.0363751	3.8016547	-1.3977437
C	1.6343179	4.8617992	-0.3919756
H	3.1013772	3.8497449	-1.6340245
H	1.4459365	3.8442947	-2.3133658
H	2.1968656	4.7406957	0.5382136
H	1.8596775	5.8488765	-0.8094382
H	0.5659317	4.8093593	-0.1735073
C	-1.7205283	0.1103743	1.6210503
B	-1.8407657	-0.0140571	-0.0342328
C	-2.2697626	1.0975715	2.4495319
C	-1.2717621	-1.0099005	2.3377074
C	-2.5492247	1.2980714	-0.7627829
C	-2.8592081	-1.3316361	-0.0990635
C	-2.2722507	1.0334952	3.8439844
F	-2.8924790	2.1944900	1.9590054
C	-1.2673638	-1.1252836	3.7232859
F	-0.8414520	-2.0962292	1.6658816
C	-2.0780655	2.5942330	-0.5519298
C	-3.5991198	1.2460528	-1.6881549
C	-4.1456334	-1.1766467	0.4322331
C	-2.5793295	-2.6435777	-0.4799219
C	-1.7566751	-0.0775506	4.4935124
F	-2.7962084	2.0448663	4.5698934
F	-0.8206883	-2.2456399	4.3285487
C	-2.5635916	3.7381803	-1.1674230
F	-1.0383131	2.8059632	0.3033080
C	-4.1185336	2.3653936	-2.3396365
F	-4.2226776	0.0900072	-2.0128134
C	-5.0880703	-2.1920938	0.5435059
F	-4.5550160	0.0430596	0.8756254
C	-3.4870150	-3.6966439	-0.3826878
F	-1.3659844	-2.9952294	-0.9585366
F	-1.7574404	-0.1537306	5.8369525
C	-3.5998606	3.6256915	-2.0840354
F	-2.0246922	4.9512533	-0.9046808
F	-5.1359135	2.2311978	-3.2213182
C	-4.7571924	-3.4763866	0.1304474
F	-6.3172760	-1.9431286	1.0495845
F	-3.1299387	-4.9430999	-0.7703043
F	-4.0907325	4.7151400	-2.7074474
F	-5.6467172	-4.4849792	0.2308975

2_II : type II isomer of **2**
117
Energy = -4245.836404452

P	-3.3851287	0.6954835	-0.1530367
C	-4.6048994	-0.5952400	-0.5725699
C	-3.1941719	1.5500002	-1.7581801
C	-3.8342944	1.8207897	1.2001802
C	-5.8812535	-0.7464705	0.0339503
C	-4.2676154	-1.3987450	-1.7015110

C	-1.9551648	1.7823627	-2.4039057
C	-4.3995149	1.8792354	-2.4321142
C	-4.0037277	1.2387648	2.4876545
C	-3.9468922	3.2275296	1.0496194
C	-6.7382318	-1.7364445	-0.4626361
C	-6.4778059	0.0990197	1.1371596
C	-5.1738473	-2.3565912	-2.1482176
C	-2.9833151	-1.2985698	-2.4913990
C	-1.9549794	2.2568357	-3.7156276
C	-0.6243816	1.5670592	-1.7542492
C	-4.3352570	2.3530514	-3.7446825
C	-5.7779947	1.8279401	-1.8155663
C	-4.3662110	2.0473360	3.5592062
C	-3.7517140	-0.2153293	2.7947960
C	-4.3179621	3.9843621	2.1687002
C	-3.6411597	4.0293187	-0.1951459
C	-6.4104110	-2.5603354	-1.5355303
H	-7.7151761	-1.8398677	0.0038903
H	-6.1703130	-0.2469798	2.1276231
H	-6.2154662	1.1540587	1.0665364
H	-7.5661395	0.0153670	1.0875825
H	-4.8920474	-2.9681635	-3.0027122
H	-3.1333123	-0.6779273	-3.3822555
H	-2.1504077	-0.8875529	-1.9239263
H	-2.6878887	-2.2982956	-2.8185116
C	-3.1293615	2.5280791	-4.4197544
H	-0.9930524	2.4182940	-4.1969252
H	-0.5287491	2.1748821	-0.8515573
H	-0.4728867	0.5306384	-1.4493091
H	0.1784599	1.8435472	-2.4395908
H	-5.2683077	2.6072842	-4.2430256
H	-6.3782681	2.6485317	-2.2194793
H	-6.2902994	0.8899473	-2.0496214
H	-5.7594020	1.9381560	-0.7291554
C	-4.5490262	3.4254692	3.4222769
H	-4.5013066	1.5817518	4.5330806
H	-4.3096925	-0.5095867	3.6873769
H	-4.0303600	-0.8948579	1.9841402
H	-2.6853909	-0.3549486	2.9981721
H	-4.4056149	5.0612963	2.0469210
H	-2.6781179	3.7604848	-0.6328946
H	-4.3960499	3.9065214	-0.9754532
H	-3.6105096	5.0879782	0.0726648
C	-7.3521610	-3.6313008	-2.0180751
C	-3.0880752	3.0218130	-5.8416951
C	-4.9596788	4.2744191	4.5958081
H	-7.0298464	-4.6140835	-1.6515345
H	-8.3696973	-3.4562267	-1.6580542
H	-7.3664993	-3.6806829	-3.1115451
H	-2.5480967	2.3154023	-6.4823209
H	-4.0958325	3.1531870	-6.2440085
H	-2.5622537	3.9815944	-5.9043730
H	-5.9790243	4.0245525	4.9139478
H	-4.3018877	4.0990465	5.4542356
H	-4.9310310	5.3377894	4.3445043

C	0.0711248	0.8511998	1.2575574
O	0.7854308	0.0297394	0.5567249
O	0.7329836	1.6902293	2.0663668
N	-1.2379730	0.9206342	1.2634403
C	-0.0404630	2.6563701	2.8433531
N	-1.9244326	-0.0309365	0.4398599
C	-0.3919076	3.8779471	2.0137723
H	0.6314546	2.9078885	3.6664316
H	-0.9335601	2.1562747	3.2236792
C	-1.8332163	-1.4154481	0.7364621
H	-1.0492096	3.5997817	1.1880584
H	-0.9189952	4.6030132	2.6433050
H	0.5088902	4.3524726	1.6142306
O	-2.4162357	-2.2817938	0.1144581
O	-1.0431656	-1.6193509	1.7975318
C	-0.8278451	-3.0159931	2.1832960
C	-0.9119128	-3.0942257	3.6937825
H	-1.5827166	-3.6286058	1.6894858
H	0.1616778	-3.2849494	1.8054437
H	-0.2136318	-2.3981132	4.1646844
H	-1.9244301	-2.8641238	4.0386909
H	-0.6599482	-4.1100033	4.0156651
B	2.2920540	-0.2306011	0.3263170
C	3.2349127	1.0337054	0.7882479
C	2.6486751	-1.6049196	1.1641623
C	2.3075643	-0.4778985	-1.3075331
C	2.9275968	2.3326420	0.3761195
C	4.3754001	0.9539924	1.5871452
C	3.2561657	-2.7429743	0.6330253
C	2.3779438	-1.6925403	2.5320890
C	3.1272855	0.1587077	-2.2377622
C	1.3715755	-1.3489632	-1.8768242
C	3.6315265	3.4666141	0.7537020
F	1.8833957	2.5385134	-0.4644379
C	5.1210211	2.0627965	1.9898175
F	4.8559244	-0.2397501	2.0238806
C	3.5450664	-3.8879208	1.3748393
F	3.6417647	-2.7925228	-0.6684193
C	2.6659036	-2.8029502	3.3173294
F	1.8381996	-0.6330342	3.1822707
C	2.9990258	0.0061731	-3.6185758
F	4.1355842	0.9796762	-1.8449198
C	1.2065847	-1.5341792	-3.2442149
F	0.5657607	-2.0984132	-1.0835973
C	4.7439951	3.3329337	1.5784048
F	3.2560323	4.6926664	0.3265205
F	6.2108626	1.9098101	2.7728868
C	3.2461041	-3.9220513	2.7303035
F	4.1258316	-4.9581451	0.7919969
F	2.3992690	-2.8065401	4.6418960
C	2.0238063	-0.8391521	-4.1296171
F	3.8175369	0.6696074	-4.4634720
F	0.2739209	-2.3850042	-3.7235666
F	5.4486108	4.4153425	1.9580402
F	3.5240747	-5.0134595	3.4670068

F	1.8826491	-0.9984791	-5.4589855

2_I : type I isomer of **2**
117
Energy = -4245.840962875

P	3.0156393	-0.1861601	-0.1649034
C	2.4816129	-1.7430514	0.6239906
C	3.7330690	1.0505901	0.9595367
C	4.1658850	-0.7700646	-1.4687133
C	2.7180404	-2.1039187	1.9804424
C	1.9161314	-2.7054681	-0.2599148
C	2.8859313	1.5201810	2.0005195
C	5.0237402	1.6185409	0.7948032
C	5.1989659	-1.6313620	-1.0090102
C	4.0323218	-0.5357768	-2.8655620
C	2.3430873	-3.3835520	2.4013435
C	3.3746829	-1.2684721	3.0566965
C	1.5535622	-3.9571379	0.2330075
C	1.7000683	-2.5105374	-1.7403226
C	3.3833521	2.4505718	2.9105380
C	1.4445366	1.1123952	2.1622395
C	5.4643340	2.5441390	1.7469503
C	5.9551118	1.3816936	-0.3699684
C	6.0459034	-2.2468443	-1.9326077
C	5.4956229	-1.9183532	0.4435220
C	4.9105936	-1.1882207	-3.7357266
C	3.0256449	0.3680869	-3.5316197
C	1.7473336	-4.3210001	1.5624403
H	2.5327227	-3.6494700	3.4384649
H	4.1676823	-0.6178632	2.6939467
H	2.6423925	-0.6352706	3.5668536
H	3.8014972	-1.9402508	3.8054392
H	1.1017778	-4.6660604	-0.4573175
H	0.7716784	-3.0074673	-2.0356112
H	1.6265918	-1.4724032	-2.0509058
H	2.5222708	-2.9694504	-2.3016403
C	4.6793594	2.9601785	2.8217577
H	2.7271615	2.7874398	3.7100570
H	1.2192937	0.0965103	1.8339391
H	1.1453357	1.2187319	3.2085885
H	0.8013972	1.7648064	1.5661702
H	6.4559337	2.9723338	1.6204701
H	6.7326125	2.1494208	-0.3577467
H	6.4508035	0.4086089	-0.3263436
H	5.4289734	1.4353893	-1.3247722
C	5.9180894	-2.0509150	-3.3045084
H	6.8337876	-2.8940601	-1.5535614
H	4.9534942	-2.8018465	0.7936479
H	6.5655319	-2.1113357	0.5610151
H	5.2374582	-1.0867912	1.1024526
H	4.7894697	-1.0081653	-4.8014995
H	2.0078843	0.2219102	-3.1708116
H	3.2854922	1.4179394	-3.3764287
H	3.0349629	0.1767680	-4.6071353
C	1.3102798	-5.6664321	2.0744773
C	5.1935234	3.9548673	3.8284720
C	6.8375796	-2.7272010	-4.2869368
H	1.5352877	-6.4576398	1.3522262
H	1.7941037	-5.9062762	3.0252789
H	0.2256745	-5.6691087	2.2380090
H	5.1695211	3.5308742	4.8388446
H	6.2204304	4.2526325	3.6019485
H	4.5660439	4.8535848	3.8413119
H	7.4514565	-1.9875845	-4.8145349
H	7.5055361	-3.4305913	-3.7829844
H	6.2648289	-3.2721045	-5.0454516
O	-1.7515626	-0.5431368	-1.1517355
C	-0.5215228	-0.1749087	-1.3549407
O	-0.1651773	-0.0268294	-2.6434823
N	0.3237602	-0.0230809	-0.3728709
C	-1.2373307	0.0240223	-3.6384656
N	1.5795489	0.5400862	-0.7782434
C	-0.5820662	0.3146742	-4.9721059
H	-1.9332527	0.8129605	-3.3432109
H	-1.7620117	-0.9338469	-3.6314512
C	1.5632826	1.8789387	-1.2023993
H	0.1252289	-0.4754130	-5.2414535
H	-1.3532106	0.3656658	-5.7475649
H	-0.0514061	1.2708938	-4.9447900
O	0.5616869	2.5091769	-1.4564894
O	2.8310978	2.3374627	-1.3009572
C	2.9666480	3.7724891	-1.5781818
C	2.7398432	4.5903068	-0.3204907
H	3.9883141	3.8624933	-1.9497913
H	2.2575164	4.0275073	-2.3682694
H	3.4264278	4.2795600	0.4716749
H	2.9195577	5.6471700	-0.5452796
H	1.7105217	4.4805676	0.0304690
C	-2.2060566	-0.3571853	1.4633332
B	-2.7244160	-0.0764400	-0.0749290
C	-2.4523137	0.4475144	2.5774148
C	-1.5872952	-1.5763585	1.7639657
C	-2.9949990	1.4997178	-0.4856598
C	-4.0531676	-1.0422570	-0.2363652
C	-2.0470009	0.1229207	3.8708521
F	-3.1317971	1.6165646	2.4715439
C	-1.1947015	-1.9535658	3.0440346
F	-1.3780596	-2.4879297	0.7870431
C	-2.1981099	2.5636278	-0.0533213
C	-3.9189113	1.8530667	-1.4740539
C	-5.2176346	-0.7276345	0.4673614
C	-4.1328471	-2.2319193	-0.9656267
C	-1.4074170	-1.0851980	4.1084167
F	-2.2868685	0.9663316	4.9010091
F	-0.6401408	-3.1615940	3.2794166
C	-2.3156975	3.8679124	-0.5248939
F	-1.2324065	2.3764673	0.8718994
C	-4.0751413	3.1435075	-1.9731324
F	-4.7274571	0.9170879	-2.0402515
C	-6.3748086	-1.4968246	0.4639718

F	-5.2629383	0.4119231	1.2057835
C	-5.2728819	-3.0366937	-0.9959123
F	-3.0978374	-2.6919652	-1.7096145
F	-1.0223715	-1.4256915	5.3552667
C	-3.2645334	4.1647962	-1.4935998
F	-1.5105925	4.8454537	-0.0581882
F	-4.9958149	3.4107773	-2.9264757
C	-6.4038985	-2.6716936	-0.2786786
F	-7.4652008	-1.1170121	1.1655551
F	-5.2863133	-4.1778817	-1.7213965
F	-3.3924297	5.4197265	-1.9674019
F	-7.5093502	-3.4420973	-0.3008727

3_III : type III isomer of **3**
90
Energy = -3891.801574726

P	-2.5099857	-0.4520423	-0.0295427
C	-2.0798699	-1.9853337	0.7720106
C	-2.8342526	-0.7749272	-1.7701901
C	-4.0187452	0.0808490	0.8092075
C	-1.8934549	-2.0005028	2.1634016
C	-2.0918323	-3.1796435	0.0380069
C	-1.7441612	-0.8310420	-2.6504431
C	-4.1357324	-0.9727912	-2.2480357
C	-5.0157360	-0.9006860	0.9508192
C	-4.2251960	1.3628674	1.3312008
C	-1.6849916	-3.2144554	2.8067106
H	-1.9120195	-1.0689130	2.7200516
C	-1.8973233	-4.3906179	0.6978428
H	-2.2498817	-3.1602908	-1.0350451
C	-1.9576472	-1.1060860	-3.9980789
H	-0.7439523	-0.6293779	-2.2847099
C	-4.3393583	-1.2475340	-3.5992625
H	-4.9861212	-0.9006966	-1.5789325
C	-6.2226231	-0.5843801	1.5697700
H	-4.8444588	-1.9127642	0.5958667
C	-5.4304901	1.6663727	1.9598405
H	-3.4424325	2.1081341	1.2966675
C	-1.6887443	-4.4070352	2.0770092
H	-1.5236676	-3.2330940	3.8799279
H	-1.9034680	-5.3175177	0.1335181
C	-3.2532693	-1.3194406	-4.4734004
H	-1.1130994	-1.1418270	-4.6793453
H	-5.3489520	-1.3982669	-3.9685926
C	-6.4331629	0.7014502	2.0700968
H	-6.9895942	-1.3459600	1.6694439
H	-5.5810573	2.6588850	2.3728920
H	-1.5329778	-5.3527636	2.5876261
H	-3.4172119	-1.5297797	-5.5258767
H	-7.3709281	0.9464610	2.5596169
N	-1.2187801	0.6830921	0.0297684
C	-1.5986424	2.0244114	-0.2876967
N	-0.0936094	0.5506118	0.8808210
O	-1.0945219	3.0143462	0.1842141
O	-2.6144723	1.9637212	-1.1605462
C	-0.3631969	1.1334326	2.1177235
C	-3.2613191	3.2284988	-1.5448942
O	-1.4844841	1.2102115	2.6091384
O	0.7570883	1.5961423	2.6890718
C	-2.7386598	3.6759472	-2.8946217
H	-4.3236534	2.9809023	-1.5708618
H	-3.0636998	3.9574348	-0.7572020
C	0.6078430	2.1823499	4.0191919
H	-2.9047810	2.9020247	-3.6496106
H	-3.2738774	4.5812823	-3.1998184
H	-1.6709228	3.9012123	-2.8438187
C	0.6642659	1.1299720	5.1119507
H	-0.3344007	2.7333807	4.0383103
H	1.4473557	2.8765781	4.0826048
H	0.5975133	1.6277055	6.0857406
H	-0.1686891	0.4285344	5.0209113
H	1.6042631	0.5729777	5.0728079
B	1.3890266	0.4592485	0.2487387
C	1.4196467	-0.6090570	-1.0252599
C	1.8342264	2.0034361	-0.1497656
C	2.4637614	-0.2626287	1.2813876
C	1.9463055	-0.4038578	-2.3076103
C	1.0462325	-1.9414836	-0.7925848
C	1.2733797	2.6604802	-1.2466750
C	2.6887172	2.8137210	0.6025653
C	3.8091103	-0.2914214	0.9002194
C	2.1981491	-0.9883561	2.4436657
C	1.9598234	-1.3783851	-3.3066511
F	2.5290830	0.7620837	-2.6678597
C	1.0525907	-2.9478700	-1.7533035
F	0.6931764	-2.3297941	0.4514402
C	1.5328600	3.9759219	-1.6033084
F	0.3745524	2.0126189	-2.0421513
C	2.9736597	4.1432879	0.2926843
F	3.3193889	2.3508941	1.7107849
C	4.8208402	-0.9217508	1.6143860
F	4.1967427	0.3323835	-0.2440743
C	3.1782744	-1.6345211	3.1953899
F	0.9377065	-1.1363342	2.9173511
C	1.4917692	-2.6572113	-3.0388650
F	2.4439365	-1.0914413	-4.5330738
F	0.6630634	-4.2026976	-1.4488877
C	2.3953637	4.7340724	-0.8214998
F	0.9411436	4.5329763	-2.6839346
F	3.8123987	4.8642044	1.0701106
C	4.5038486	-1.6011703	2.7846575
F	6.1010089	-0.8824351	1.1847665
F	2.8441485	-2.3094048	4.3182003
F	1.4962843	-3.6038806	-3.9931603
F	2.6577507	6.0176118	-1.1347134
F	5.4614143	-2.2246087	3.4983189

3_II : type II isomer of **3**
90
Energy = -3891.800516039

P	-3.1695074	0.3844055	0.0392027
C	-4.3640652	-0.8471949	-0.4737558
C	-2.4734909	1.1544048	-1.4271555
C	-3.9899230	1.6651388	0.9893085
C	-5.6653246	-0.7936258	0.0430772
C	-4.0171635	-1.8263604	-1.4211547
C	-1.4124287	2.0651584	-1.2916489
C	-3.0529696	0.9408264	-2.6882941
C	-4.2162129	1.4764759	2.3612572
C	-4.4483498	2.8223613	0.3466840
C	-6.6152203	-1.7230466	-0.3812637
H	-5.9370693	-0.0313682	0.7657841
C	-4.9737775	-2.7394175	-1.8450524
H	-3.0041116	-1.8804491	-1.8038502
C	-0.9185930	2.7249662	-2.4115010
H	-0.9851976	2.2596804	-0.3172134
C	-2.5549639	1.6125733	-3.8030311
H	-3.8914386	0.2636788	-2.8012761
C	-4.9044755	2.4466776	3.0821746
H	-3.8338282	0.5911798	2.8576671
C	-5.1372511	3.7891140	1.0781329
H	-4.2645925	2.9699533	-0.7128279
C	-6.2717887	-2.6902490	-1.3247588
H	-7.6219497	-1.6839447	0.0226463
H	-4.7074170	-3.4969668	-2.5753626
C	-1.4860725	2.4981056	-3.6672419
H	-0.0907194	3.4168750	-2.3021853
H	-3.0039929	1.4401902	-4.7758554
C	-5.3645925	3.6014557	2.4420108
H	-5.0751347	2.3079291	4.1452348
H	-5.4903833	4.6879815	0.5827581
H	-7.0145073	-3.4087787	-1.6583578
H	-1.0977861	3.0166361	-4.5386221
H	-5.8962874	4.3584734	3.0107371
N	-1.9972075	-0.2455928	1.0747529
C	-1.8604840	-1.6005159	1.4234221
N	-1.3215035	0.7766950	1.8227159
O	-2.3908251	-2.5148731	0.8190931
O	-1.0935541	-1.7145689	2.5128281
C	-0.0310452	0.8166885	1.5536635
C	-0.8620905	-3.0834064	2.9810404
O	0.5482453	0.0939852	0.6549909
O	0.7267002	1.6544263	2.2631235
C	-0.4631062	-2.9900424	4.4381209
H	-1.7781945	-3.6572922	2.8320623
H	-0.0719663	-3.5104003	2.3563617
C	0.0652926	2.5102000	3.2536268
H	0.4221395	-2.3623978	4.5631474
H	-1.2807446	-2.5756808	5.0352930
H	-0.2334248	-3.9932441	4.8113129
C	-0.6137709	3.7027870	2.6032398
H	0.8907115	2.8180381	3.8977721
H	-0.6415766	1.8949018	3.8138954
H	-1.4532911	3.3796454	1.9832349
H	-0.9980931	4.3634811	3.3880932
H	0.0951659	4.2697675	1.9925631
B	1.9510966	-0.0367099	0.0224647
C	2.9598459	1.1787090	0.4697450
C	2.4832552	-1.5163266	0.5034811
C	1.5845301	-0.0110362	-1.5879869
C	2.5733962	2.5103318	0.3055512
C	4.2293515	1.0268795	1.0270683
C	2.9757106	-2.5145424	-0.3358130
C	2.4668358	-1.8614695	1.8569617
C	2.2251240	0.7449210	-2.5688871
C	0.5182683	-0.7791551	-2.0701198
C	3.3303088	3.6058447	0.6924256
F	1.3822355	2.7932918	-0.2827285
C	5.0328895	2.0951242	1.4281958
F	4.7831618	-0.1997113	1.2080748
C	3.3889149	-3.7687715	0.1117136
F	3.1089519	-2.3059981	-1.6714737
C	2.8762692	-3.0943414	2.3515118
F	2.0775518	-0.9468885	2.7798753
C	1.8217496	0.7904667	-3.9025504
F	3.3204413	1.4937465	-2.2757157
C	0.0836537	-0.7687590	-3.3900310
F	-0.1431234	-1.6329980	-1.2498882
C	4.5798525	3.3968858	1.2678419
F	2.8732586	4.8649106	0.5154685
F	6.2505862	1.8734122	1.9677363
C	3.3359260	-4.0650413	1.4671265
F	3.8482832	-4.6941630	-0.7571365
F	2.8518578	-3.3579932	3.6762058
C	0.7357149	0.0331067	-4.3184910
F	2.4706663	1.5720391	-4.7929384
F	-0.9597367	-1.5333407	-3.7811394
F	5.3390292	4.4398461	1.6508744
F	3.7349673	-5.2673477	1.9216400
F	0.3206025	0.0744639	-5.5983313

3_I : type I isomer of **3**
90
Energy = -3891.804959701

P	-3.3611088	0.6201254	-0.2273438
C	-4.8394865	0.1876124	0.6944461
C	-3.7443365	1.2088585	-1.8715936
C	-2.5559451	1.9045836	0.7277451
C	-4.7497873	-0.7484425	1.7345352
C	-6.0303256	0.8939751	0.4798022
C	-2.8239724	2.0541170	-2.5151953
C	-4.8496693	0.7031035	-2.5743301
C	-1.7620642	1.5627317	1.8332714
C	-2.9131263	3.2420159	0.4986779
C	-5.8596073	-0.9959459	2.5362016
H	-3.8200935	-1.2812528	1.9092520
C	-7.1378318	0.6375131	1.2873086
H	-6.0948790	1.6412127	-0.3045537
C	-3.0244366	2.4028359	-3.8472222
H	-1.9547806	2.4221385	-1.9811027

C	-5.0426237	1.0633760	-3.9052057
H	-5.5431179	0.0272710	-2.0868370
C	-1.3104717	2.5633716	2.6871438
H	-1.4735218	0.5339040	1.9998751
C	-2.4532637	4.2350652	1.3614462
H	-3.5461356	3.5087967	-0.3413921
C	-7.0549921	-0.3079616	2.3103623
H	-5.7937158	-1.7267859	3.3361782
H	-8.0627575	1.1792003	1.1161069
C	-4.1329654	1.9116626	-4.5408695
H	-2.3149074	3.0566922	-4.3446794
H	-5.9004033	0.6781504	-4.4475640
C	-1.6534765	3.8972977	2.4535429
H	-0.6799432	2.3002413	3.5305383
H	-2.7185382	5.2708657	1.1758608
H	-7.9195217	-0.5059565	2.9366047
H	-4.2862270	2.1874592	-5.5798919
H	-1.2916042	4.6746016	3.1198637
N	-2.3202841	-0.7110481	-0.2523723
C	-2.7161997	-1.9952199	-0.6767819
N	-0.9168928	-0.4552217	-0.1357867
O	-1.9884643	-2.9608986	-0.6932607
O	-4.0122181	-1.9557006	-1.0531150
C	-0.3025787	-0.5262356	-1.2938724
C	-4.6267737	-3.2499306	-1.3774277
O	0.9884955	-0.4343557	-1.3847146
O	-0.9770859	-0.6845142	-2.4385852
C	-5.1422862	-3.9343751	-0.1261827
H	-5.4315035	-2.9852991	-2.0646172
H	-3.8757498	-3.8493524	-1.8940747
C	-0.2167607	-0.8391117	-3.6827726
H	-5.8753189	-3.3075435	0.3887856
H	-5.6256895	-4.8758466	-0.4078602
H	-4.3197532	-4.1623419	0.5578266
C	0.2493516	-2.2720251	-3.8602660
H	0.6146674	-0.1325094	-3.6692065
H	-0.9381526	-0.5461492	-4.4476575
H	0.9587166	-2.5486501	-3.0772705
H	-0.6005582	-2.9601210	-3.8301374
H	0.7440647	-2.3701058	-4.8329885
B	1.9031881	-0.2725791	-0.1693308
C	1.5430324	-1.5357086	0.8160621
C	3.4303777	-0.3305596	-0.7946543
C	1.7717388	1.2378630	0.4701921
C	0.9392732	-1.4785956	2.0698253
C	1.6845966	-2.8303661	0.3123209
C	3.7555135	0.4218003	-1.9275283
C	4.5078354	-1.0253847	-0.2424355
C	2.5295539	1.5739035	1.5942636
C	1.0912132	2.3154957	-0.0984073
C	0.5018925	-2.5989173	2.7702818
F	0.6891624	-0.2900991	2.6767608
C	1.2644325	-3.9790881	0.9718809
F	2.2492228	-3.0243117	-0.9098270
C	5.0237921	0.4629531	-2.4987757
F	2.8157775	1.1943003	-2.5307629
C	5.7954438	-1.0079292	-0.7756330
F	4.3564946	-1.7797833	0.8744656
C	2.5825835	2.8440977	2.1547121
F	3.2859165	0.6197053	2.1953089
C	1.1345101	3.6118479	0.4114859
F	0.3355556	2.1728459	-1.2186359
C	0.6639508	-3.8633693	2.2191249
F	-0.1039488	-2.4665200	3.9730938
F	1.4317048	-5.1998875	0.4181849
C	6.0579121	-0.2612475	-1.9171720
F	5.2643725	1.2080346	-3.5998391
F	6.7914372	-1.7112217	-0.1947470
C	1.8744739	3.8800254	1.5542053
F	3.3185869	3.0877859	3.2609927
F	0.4571223	4.6106295	-0.1949702
F	0.2422203	-4.9586311	2.8818720
F	7.2950953	-0.2330664	-2.4475799
F	1.9072672	5.1229598	2.0734592

4_III : type III isomer of **4**
90
Energy = -2402.546970362

P	1.8770054	-0.2004428	0.0863059
C	1.6858310	-1.9786176	0.2213747
C	2.2256811	0.4882674	1.7110110
C	3.2493580	0.1469111	-1.0121458
C	2.6015211	-2.8010149	-0.4556208
C	0.6645966	-2.5525263	0.9948172
C	2.0460687	-0.2787569	2.8718447
C	2.6480268	1.8268954	1.8183449
C	4.5114777	0.4739708	-0.5000361
C	3.0462226	0.0303461	-2.3971156
C	2.4880610	-4.1851374	-0.3628709
H	3.3906855	-2.3623860	-1.0554086
C	0.5558489	-3.9380901	1.0712910
H	-0.0593593	-1.9299721	1.5066854
C	2.2669001	0.2929323	4.1233938
H	1.7358449	-1.3143409	2.8042233
C	2.8728394	2.3850397	3.0712466
H	2.7794001	2.4218213	0.9239414
C	5.5683082	0.7071088	-1.3791281
H	4.6689521	0.5479645	0.5707087
C	4.1086125	0.2702577	-3.2633744
H	2.0655729	-0.2536935	-2.7707585
C	1.4636736	-4.7549070	0.3959544
H	3.1959432	-4.8168940	-0.8905375
H	-0.2480495	-4.3708153	1.6574445
C	2.6758309	1.6220926	4.2258708
H	2.1200013	-0.3052961	5.0170645
H	3.1978764	3.4180570	3.1483774
C	5.3659723	0.6130632	-2.7569630
H	6.5467582	0.9649215	-0.9860068
H	3.9568570	0.1877144	-4.3351122
H	1.3737183	-5.8354248	0.4596377

H	2.8450840	2.0642602	5.2030656
H	6.1900952	0.8024145	-3.4383682
N	0.4591642	0.4412451	-0.6194316
C	0.4495612	1.6183003	-1.3927427
N	-0.7235827	-0.3177105	-0.5895010
O	-0.3754202	1.8719382	-2.2399263
O	1.4885395	2.3947946	-1.0388963
C	-0.6913166	-1.3145071	-1.5392091
C	1.6736139	3.6052438	-1.8450027
O	0.1661057	-1.4447301	-2.4149789
O	-1.7087718	-2.1765198	-1.3596399
C	2.9704211	4.2423849	-1.3957737
H	1.6971947	3.3094306	-2.8966239
H	0.8066343	4.2474703	-1.6718651
C	-1.9269312	-3.1352923	-2.4294375
H	3.8119707	3.5578169	-1.5389843
H	3.1506174	5.1430532	-1.9906448
H	2.9222475	4.5343113	-0.3420871
C	-3.2557865	-3.8052388	-2.1417370
H	-1.0929079	-3.8441263	-2.4362213
H	-1.9375676	-2.5969874	-3.3814347
H	-3.4809698	-4.5273925	-2.9335569
H	-3.2245798	-4.3384264	-1.1861281
H	-4.0550167	-3.0591769	-2.1026588
B	-2.0306707	0.2118604	0.2890594
C	-1.8370742	1.8148667	0.5805619
C	-3.3511938	-0.0052548	-0.6385020
C	-2.0862972	-0.6849874	1.6643175
C	-2.7859289	2.7553514	0.1336872
C	-0.7494158	2.3526256	1.2970557
C	-3.3340294	0.2474273	-2.0234476
C	-4.5931035	-0.3607269	-0.0883930
C	-1.6160207	-0.2448631	2.9143193
C	-2.5638782	-2.0137393	1.6255756
C	-2.6531530	4.1266261	0.3638831
H	-3.6496976	2.4045089	-0.4240201
C	-0.5932049	3.7203831	1.5289963
H	0.0192332	1.6892458	1.6754799
C	-4.4714485	0.1111220	-2.8188969
H	-2.3996674	0.5592039	-2.4849423
C	-5.7457467	-0.4937534	-0.8696848
H	-4.6628613	-0.5493807	0.9808170
C	-1.5689688	-1.0782203	4.0376774
H	-1.2690604	0.7779799	3.0239114
C	-2.5375828	-2.8534255	2.7389672
H	-2.9448469	-2.3993045	0.6863068
C	-1.5480142	4.6221855	1.0586463
H	-3.4115212	4.8121887	-0.0090731
H	0.2771558	4.0785808	2.0747166
C	-5.6891182	-0.2686496	-2.2456378
H	-4.4121947	0.3023232	-3.8888901
H	-6.6871183	-0.7789100	-0.4035882
C	-2.0234768	-2.3939744	3.9550300
H	-1.1783134	-0.6950408	4.9782074
H	-2.9105837	-3.8727804	2.6581297
H	-1.4351883	5.6897172	1.2322137
H	-6.5787323	-0.3806637	-2.8611718
H	-1.9879847	-3.0477553	4.8231629

4_II : type II isomer of **4**
90
Energy = -2402.539354555

P	-3.4258414	-0.4125702	0.0719178
C	-5.1665965	-0.3428663	0.5020578
C	-2.8915109	-2.1071149	0.2957905
C	-3.1441495	0.1302770	-1.6157013
C	-5.6344146	0.5303485	1.4911436
C	-6.0533229	-1.2218047	-0.1432224
C	-1.8094884	-2.6101036	-0.4377054
C	-3.4991396	-2.8890432	1.2918107
C	-4.2003471	0.2756145	-2.5260495
C	-1.8258498	0.4085874	-2.0062458
C	-6.9886503	0.5352096	1.8211791
H	-4.9470407	1.1928938	2.0040047
C	-7.4056148	-1.2041167	0.1878471
H	-5.6870181	-1.9250259	-0.8855933
C	-1.3195618	-3.8826822	-0.1555325
H	-1.3334977	-2.0115862	-1.2054665
C	-3.0142930	-4.1664604	1.5529669
H	-4.3317670	-2.4951731	1.8649790
C	-3.9264677	0.6822008	-3.8303227
H	-5.2250259	0.0929394	-2.2205524
C	-1.5648164	0.8196090	-3.3101458
H	-1.0195286	0.3401010	-1.2840961
C	-7.8731662	-0.3244291	1.1674792
H	-7.3505075	1.2085197	2.5918950
H	-8.0904499	-1.8819294	-0.3120076
C	-1.9200476	-4.6591552	0.8359439
H	-0.4610875	-4.2522034	-0.7057369
H	-3.4826632	-4.7724190	2.3224621
C	-2.6125131	0.9512482	-4.2233853
H	-4.7425396	0.7998109	-4.5367069
H	-0.5438377	1.0559498	-3.5939679
H	-8.9272722	-0.3169391	1.4280505
H	-1.5366150	-5.6521886	1.0517708
H	-2.4101369	1.2761140	-5.2398310
N	-2.4720929	0.4807456	1.1515708
C	-2.4173633	1.8726508	1.1424507
N	-1.6932471	-0.2553224	2.0997888
O	-1.6545804	2.5406015	1.8083306
O	-3.3729729	2.3272480	0.2975614
C	-0.4525815	-0.4047498	1.6639697
C	-3.3867531	3.7589514	-0.0193387
O	0.0226781	0.1195652	0.5853746
O	0.3787545	-1.1577361	2.3836802
C	-3.1199923	3.9382495	-1.5008696
H	-4.3823829	4.1060084	0.2660356
H	-2.6355193	4.2435405	0.6062393
C	-0.1568420	-1.8280759	3.5619584
H	-3.1660564	5.0048501	-1.7454978

H	-2.1296507	3.5602242	-1.7665321
H	-3.8674512	3.4110367	-2.0999160
C	0.9459638	-2.7263662	4.0831382
H	-1.0470008	-2.3875774	3.2626230
H	-0.4504407	-1.0644342	4.2881003
H	0.6012613	-3.2379992	4.9877414
H	1.2156075	-3.4791420	3.3364375
H	1.8371604	-2.1417332	4.3302985
B	1.5070227	0.0317586	0.0333390
C	2.5465526	0.5115460	1.1797664
C	1.7345041	-1.4822597	-0.5211668
C	1.5289440	1.1704324	-1.1313102
C	2.1614721	1.3659689	2.2283256
C	3.9099566	0.1782810	1.1098262
C	1.3132031	-1.8661813	-1.8079357
C	2.3180479	-2.4910585	0.2661225
C	2.3638814	1.0757269	-2.2585422
C	0.7841080	2.3579519	-1.0025427
C	3.0757949	1.8382287	3.1710231
H	1.1192972	1.6693179	2.3133776
C	4.8382422	0.6469230	2.0428343
H	4.2546065	-0.4698488	0.3057721
C	1.4765691	-3.1662458	-2.2935567
H	0.8532112	-1.1224156	-2.4557113
C	2.4882142	-3.7964404	-0.2006392
H	2.6458361	-2.2421435	1.2715365
C	2.4348388	2.0885557	-3.2193229
H	2.9713159	0.1829582	-2.3931672
C	0.8454031	3.3784244	-1.9527716
H	0.1407569	2.4812729	-0.1348825
C	4.4235708	1.4778582	3.0858682
H	2.7388388	2.4897680	3.9753143
H	5.8853522	0.3617991	1.9585428
C	2.0738995	-4.1404753	-1.4897846
H	1.1403164	-3.4198258	-3.2968888
H	2.9460931	-4.5477128	0.4397040
C	1.6681978	3.2469004	-3.0743846
H	3.0871269	1.9731106	-4.0827167
H	0.2527024	4.2814555	-1.8206484
H	5.1394429	1.8419990	3.8191606
H	2.2085842	-5.1538708	-1.8606373
H	1.7151872	4.0366157	-3.8203026

4_I : type I isomer of **4**
90
Energy = -2402.529693387

P	-2.3747150	-0.4604897	0.1264139
C	-3.9741775	-0.3049285	-0.6688506
C	-2.5259658	-0.4560137	1.9094535
C	-1.6770271	-2.0154169	-0.4255497
C	-4.0192146	0.0716676	-2.0195262
C	-5.1439667	-0.6872019	-0.0011253
C	-1.6622136	-1.2513695	2.6784880
C	-3.3834877	0.4661921	2.5332889
C	-0.7408517	-2.1015759	-1.4664958
C	-2.2384772	-3.1802073	0.1240921
C	-5.2385314	0.0895148	-2.6881727
H	-3.1073960	0.3530553	-2.5381715
C	-6.3617184	-0.6687260	-0.6812498
H	-5.1088009	-0.9983241	1.0378209
C	-1.6689673	-1.1348529	4.0661734
H	-0.9908458	-1.9491393	2.1896643
C	-3.3892029	0.5661326	3.9214723
H	-4.0195773	1.1074227	1.9328475
C	-0.3589653	-3.3557702	-1.9331258
H	-0.2860656	-1.2016735	-1.8608440
C	-1.8430784	-4.4275707	-0.3506968
H	-2.9777178	-3.1155210	0.9175941
C	-6.4105599	-0.2774339	-2.0194722
H	-5.2754941	0.3881650	-3.7312426
H	-7.2697781	-0.9604005	-0.1626760
C	-2.5344852	-0.2319924	4.6870593
H	-1.0011065	-1.7492950	4.6620766
H	-4.0550208	1.2731218	4.4066266
C	-0.9013294	-4.5146795	-1.3772815
H	0.3871300	-3.4265391	-2.7178356
H	-2.2671320	-5.3271841	0.0844801
H	-7.3605053	-0.2623692	-2.5452914
H	-2.5404395	-0.1462380	5.7695813
H	-0.5831734	-5.4883938	-1.7374988
N	-1.3622396	0.7782774	-0.4017327
C	-1.8425778	2.0932818	-0.4570442
N	0.0380276	0.4701393	-0.4127923
O	-2.9844364	2.3925122	-0.1330075
O	-0.9086001	2.9198055	-0.9225282
C	0.6755956	0.9758268	0.6203413
C	-1.2672544	4.3390672	-0.9289477
O	1.9455188	0.8398843	0.7835775
O	0.0160237	1.6860602	1.5604275
C	-0.0177879	5.0973853	-1.3218732
H	-2.0838969	4.4806460	-1.6422140
H	-1.6223212	4.6030270	0.0710772
C	0.7943627	2.2006003	2.6814981
H	0.3301738	4.7848180	-2.3100666
H	-0.2406644	6.1688602	-1.3482350
H	0.7846993	4.9231219	-0.5992547
C	-0.1597758	3.0076194	3.5370650
H	1.6145683	2.8056408	2.2869181
H	1.2169200	1.3512797	3.2263199
H	-0.5780630	3.8448377	2.9697982
H	-0.9813882	2.3816678	3.8962823
H	0.3785973	3.4088783	4.4017995
B	2.8683721	0.0278921	-0.2056326
C	2.6411672	0.6101139	-1.7055077
C	4.3702136	0.3414495	0.3320848
C	2.5625452	-1.5530184	-0.0059782
C	2.2153071	-0.1521690	-2.8035304
C	2.8539221	1.9805778	-1.9389238
C	4.6417559	0.6236800	1.6826423
C	5.4774335	0.2745817	-0.5316745

C	3.2072446	-2.5201573	-0.7996239
C	1.7157399	-2.0446731	1.0016932
C	1.9995699	0.4145601	-4.0620832
H	2.0299671	-1.2146780	-2.6663530
C	2.6324483	2.5664900	-3.1852259
H	3.1874754	2.6059037	-1.1119690
C	5.9426637	0.8302819	2.1487656
H	3.8124764	0.6869593	2.3838131
C	6.7847523	0.4680974	-0.0793739
H	5.3076072	0.0770245	-1.5887391
C	2.9968071	-3.8890427	-0.6235833
H	3.8997323	-2.1932897	-1.5730119
C	1.4974347	-3.4098785	1.1949863
H	1.2141206	-1.3386843	1.6590962
C	2.2028396	1.7820680	-4.2588286
H	1.6630350	-0.2084297	-4.8890411
H	2.7955049	3.6336647	-3.3238718
C	7.0249923	0.7495996	1.2681503
H	6.1153356	1.0530928	3.2002573
H	7.6170913	0.4086693	-0.7781716
C	2.1337619	-4.3436701	0.3761336
H	3.5057901	-4.6036898	-1.2674193
H	0.8226394	-3.7496213	1.9787279
H	2.0283226	2.2308082	-5.2342263
H	8.0396921	0.9076160	1.6260399
H	1.9571607	-5.4077430	0.5114511

5_III : type III isomer of **5**
116
Energy = -2793.441727947

P	2.2636592	-0.0392609	-0.1172177
C	1.7492408	-1.4692264	0.8345220
C	3.1780060	1.1175998	0.9438368
C	3.3413329	-0.7656345	-1.4151634
C	1.9877592	-1.5736129	2.2079948
C	1.2184045	-2.5676433	0.1366840
C	2.4893464	1.7026358	2.0454094
C	4.5614041	1.4104808	0.7573406
C	4.0999490	-1.9228159	-1.0885762
C	3.4417126	-0.2312237	-2.7242164
C	1.6766565	-2.7499651	2.8768546
H	2.4377746	-0.7487358	2.7494358
C	0.8921813	-3.7302379	0.8151435
H	1.0887064	-2.5092491	-0.9380201
C	3.1955862	2.4697250	2.9694839
C	1.0076259	1.5966325	2.2450176
C	5.2042673	2.1974924	1.7209165
C	5.4434626	1.0013715	-0.4017862
C	4.7474918	-2.6128340	-2.1196763
C	4.4136599	-2.4111373	0.3114851
C	4.1190054	-0.9522190	-3.7050868
C	2.9282316	1.1266002	-3.1139304
C	1.0982066	-3.8394494	2.2037500
H	1.8665019	-2.8245441	3.9438299
H	0.4832346	-4.5680199	0.2597823
C	4.5610404	2.7198459	2.8404833
H	2.6463042	2.8951825	3.8061058
H	0.6032763	0.6106666	2.0262300
H	0.7412748	1.8440338	3.2746554
H	0.4920635	2.2982137	1.5823787
H	6.2597627	2.4128616	1.5737442
H	6.4037589	1.5115849	-0.2986671
H	5.6388945	-0.0722613	-0.4303525
H	5.0179651	1.2808407	-1.3665293
C	4.7345476	-2.1782929	-3.4420887
H	5.3130553	-3.5047822	-1.8583527
H	3.7230029	-3.1863227	0.6556968
H	5.4192400	-2.8418608	0.3030146
H	4.4032286	-1.6029353	1.0457030
H	4.1753305	-0.5284515	-4.7054138
H	1.8500653	1.1219648	-3.2778808
H	3.1589593	1.8708380	-2.3473339
H	3.4076497	1.4471075	-4.0420577
C	0.7315297	-5.0358802	2.9671018
C	5.3091247	3.5246692	3.8688382
C	5.4297272	-2.9503595	-4.5320803
C	-0.0365804	-6.0435623	2.5297146
H	1.1078034	-5.0657914	3.9889563
H	5.5460036	2.9004655	4.7397528
H	6.2502038	3.9077947	3.4651689
H	4.7068044	4.3658209	4.2260495
H	6.3428324	-2.4326231	-4.8504657
H	5.7070442	-3.9515544	-4.1923361
H	4.7875246	-3.0447063	-5.4142185
H	-0.2695598	-6.8867596	3.1720833
H	-0.4667867	-6.0570087	1.5318339
N	0.7486175	0.6274834	-0.6780990
N	-0.4237094	-0.1792633	-0.8787113
C	0.7062094	1.8885540	-1.3341554
C	-0.5418427	-0.6913398	-2.1619124
O	-0.0242626	2.1513965	-2.2592557
O	1.5993922	2.7137928	-0.7589084
O	-1.5939859	-0.9704057	-2.7098042
O	0.6738318	-0.9645857	-2.7282142
C	1.7897353	4.0315348	-1.3748435
C	0.6111202	-1.6203959	-4.0295520
C	1.0700725	5.0833928	-0.5552110
H	2.8720585	4.1774908	-1.3681960
H	1.4221563	3.9833062	-2.4009447
C	0.3968977	-0.6284971	-5.1602186
H	-0.1909155	-2.3605591	-3.9960269
H	1.5773860	-2.1219656	-4.1093311
H	1.4122534	5.0663328	0.4839560
H	1.2811641	6.0727770	-0.9746148
H	-0.0089627	4.9153427	-0.5810754
H	1.2296902	0.0759271	-5.2345535
H	0.3241827	-1.1739402	-6.1078317
H	-0.5298726	-0.0703507	-5.0076197
B	-1.8412160	0.1306034	-0.0672500
C	-1.7037988	0.1191484	1.5679776

C -2.4663727 1.5105308 -0.6686361
C -2.8232678 -1.1622345 -0.2454491
C -2.3179797 1.0824021 2.3896914
C -1.1823436 -1.0048448 2.2376768
C -2.1186664 2.7856283 -0.1861104
C -3.3533266 1.4905574 -1.7606823
C -4.2064422 -1.0192923 -0.0464145
C -2.3620406 -2.4877444 -0.3804792
C -2.3495915 0.9716050 3.7827465
H -2.8012884 1.9405630 1.9345193
C -1.2040131 -1.1316577 3.6246224
H -0.7721930 -1.8191501 1.6504252
C -2.6326306 3.9635847 -0.7316823
H -1.4119814 2.8675654 0.6377542
C -3.8722091 2.6570663 -2.3249658
H -3.6323173 0.5294169 -2.1816983
C -5.0678486 -2.1137907 0.0007085
H -4.6188753 -0.0220882 0.0901819
C -3.2038838 -3.5883018 -0.3251457
H -1.3008514 -2.6611659 -0.5296833
C -1.7782870 -0.1327286 4.4142249
H -2.8286374 1.7496053 4.3743297
H -0.7831728 -2.0187125 4.0915886
C -3.5197517 3.9055811 -1.8072521
H -2.3457600 4.9280058 -0.3168022
H -4.5536599 2.5941926 -3.1714618
C -4.5897987 -3.4283118 -0.1346698
H -6.1352042 -1.9569669 0.1545453
H -2.7849901 -4.5867516 -0.4345040
H -1.7992454 -0.2246992 5.4976447
H -3.9273563 4.8180790 -2.2364734
C -5.5326210 -4.5461051 -0.0662992
C -5.2585655 -5.8597498 -0.1464856
H -6.5743569 -4.2492372 0.0685800
H -6.0497376 -6.6006329 -0.0799232
H -4.2472343 -6.2368741 -0.2777851

5_II : type II isomer of **5**
116
Energy = -2793.459838552
P -3.3782443 0.5568471 0.1628395
C -4.7456232 -0.4511499 -0.4768517
C -2.7113628 1.2758852 -1.3459653
C -3.8353947 1.7526168 1.4437944
C -6.1101531 -0.2296606 -0.1474352
C -4.4061385 -1.4085550 -1.4750789
C -1.3389967 1.2716380 -1.6199978
C -3.6132347 1.7103819 -2.3297866
C -4.2080558 1.2046174 2.6976124
C -3.7773720 3.1599750 1.2759074
C -7.0765397 -1.0316028 -0.7656248
C -6.6494556 0.8369996 0.7789362
C -5.4190373 -2.1751795 -2.0459751
C -3.0148290 -1.6420686 -2.0149065
C -0.8729201 1.6978440 -2.8545715
H -0.6393743 0.8725486 -0.8962134
C -3.1362731 2.1622354 -3.5526582
H -4.6837657 1.6813455 -2.1488552
C -4.5404213 2.0650385 3.7424521
C -4.2430497 -0.2752308 2.9892548
C -4.1269952 3.9683498 2.3609304
C -3.3447450 3.8822299 0.0219792
C -6.7606400 -2.0211145 -1.6946316
H -8.1197278 -0.8567571 -0.5128346
H -6.4728530 0.6004140 1.8314526
H -6.2101821 1.8186654 0.5912655
H -7.7296882 0.9115417 0.6329275
H -5.1432104 -2.9144067 -2.7951353
H -2.8977881 -1.1217640 -2.9731327
H -2.2166632 -1.3005695 -1.3601190
H -2.8690587 -2.7109311 -2.1904505
C -1.7586537 2.1563548 -3.8450645
H 0.1932837 1.6416429 -3.0502041
H -3.8394037 2.4998091 -4.3095520
C -4.5102498 3.4516891 3.5991988
H -4.8326461 1.6312897 4.6963159
H -4.9350763 -0.4743880 3.8115808
H -4.5491263 -0.8856791 2.1338471
H -3.2463734 -0.6086212 3.3010097
H -4.0833845 5.0468564 2.2271235
H -2.3486305 3.5739515 -0.3054258
H -4.0278317 3.7093826 -0.8139096
H -3.3229576 4.9561731 0.2211257
C -7.8288142 -2.8923488 -2.3006020
C -1.3115429 2.5952495 -5.1710057
C -4.8525005 4.3653581 4.7465418
H -7.8820678 -3.8514644 -1.7698939
H -8.8122532 -2.4188816 -2.2331372
H -7.6114670 -3.1117133 -3.3504916
C -0.0625253 2.5125214 -5.6517806
H -2.0928417 3.0119637 -5.8061810
H -3.9509388 4.8677019 5.1184206
H -5.5518044 5.1472404 4.4318843
H -5.2972695 3.8094807 5.5761067
H 0.1732678 2.8624884 -6.6519777
H 0.7542011 2.0850707 -5.0767592
N -2.1146748 -0.3271712 0.9112189
C -2.1544302 -1.6951535 1.2132044
N -1.3496850 0.5492540 1.7619995
O -2.9649546 -2.4801568 0.7493858
O -1.1732780 -2.0065809 2.0730352
C -0.0557277 0.5163872 1.5104195
C -1.0832703 -3.4201407 2.4412893
O 0.5067062 -0.1119487 0.5373848
O 0.7530931 1.1743234 2.3502746
C -1.9667064 -3.7183590 3.6386404
H -1.3571572 -4.0204473 1.5717451
H -0.0239962 -3.5550489 2.6653746
C 0.1500882 1.9850386 3.4043951
H -1.7023332 -3.0772991 4.4851292

H	-3.0206202	-3.5663075	3.3894577
H	-1.8296410	-4.7629326	3.9386850
C	-0.2966885	3.3329023	2.8665698
H	0.9590242	2.0895337	4.1302759
H	-0.6802756	1.4255703	3.8411088
H	-1.1127152	3.2052084	2.1526920
H	-0.6578456	3.9526276	3.6951620
H	0.5370728	3.8444696	2.3773846
B	2.0007537	-0.0417695	0.0213248
C	2.6097492	1.4477721	0.2481826
C	2.8371643	-1.2023485	0.7777397
C	1.8236099	-0.3781085	-1.5632544
C	1.9696010	2.5721471	-0.3043856
C	3.7764338	1.6937976	0.9874664
C	4.0267117	-1.7058408	0.2234384
C	2.4515068	-1.7494308	2.0169213
C	2.5916976	0.2477785	-2.5597124
C	0.8779164	-1.3236054	-2.0005220
C	2.4443812	3.8690719	-0.1125501
H	1.0665928	2.4312167	-0.8941278
C	4.2732916	2.9862718	1.1822982
H	4.3038838	0.8521089	1.4320621
C	4.7882814	-2.6813208	0.8638872
H	4.3628380	-1.3250846	-0.7397141
C	3.1969302	-2.7308921	2.6630127
H	1.5389788	-1.3965157	2.4889974
C	2.4213814	-0.0397780	-3.9181412
H	3.3278135	0.9933459	-2.2661701
C	0.6841391	-1.6094427	-3.3522260
H	0.2628423	-1.8288833	-1.2591325
C	3.6045624	4.0838440	0.6370628
H	1.9132948	4.7140743	-0.5465088
H	5.1799828	3.1383004	1.7649013
C	4.3897333	-3.2203471	2.1002988
H	5.7056654	-3.0456217	0.4032433
H	2.8535628	-3.1192734	3.6197409
C	1.4567371	-0.9644224	-4.3221165
H	3.0323848	0.4679674	-4.6619414
H	-0.0731618	-2.3301634	-3.6536756
H	3.9826242	5.0919036	0.7903822
C	5.2198345	-4.2515679	2.7313311
H	1.3054337	-1.1768165	-5.3775370
C	4.9954283	-4.8847266	3.8942200
H	6.1166191	-4.5153937	2.1685391
H	5.6874814	-5.6358086	4.2630469
H	4.1254404	-4.6788366	4.5129025

5_I : type I isomer of **5**

116

Energy = -2793.456065086

P	2.8240969	0.1922286	-0.2745410
C	2.0770267	-1.3135242	0.3930389
C	3.7700758	1.0204639	1.0290052
C	3.7867031	-0.4467865	-1.6817384
C	2.1518007	-1.6573482	1.7501886
C	1.5322091	-2.2373263	-0.5113662
C	3.0527269	1.4010029	2.1976349
C	5.1531232	1.3162549	0.9196517
C	4.6347600	-1.5512414	-1.3868073
C	3.6621675	0.0033733	-3.0248526
C	1.6569069	-2.8725063	2.1964073
H	2.6231010	-0.9859057	2.4581135
C	1.0107730	-3.4388580	-0.0538830
H	1.5225356	-2.0124107	-1.5708169
C	3.7677798	1.9202912	3.2783863
C	1.5465980	1.3923523	2.3183751
C	5.8061249	1.8491893	2.0345166
C	5.9738287	1.1763130	-0.3400966
C	5.2958300	-2.1992810	-2.4308956
C	4.9129344	-2.0853574	0.0020374
C	4.3431440	-0.7013982	-4.0221936
C	2.8890050	1.2118282	-3.4906217
C	1.0514805	-3.7804536	1.3099315
H	1.7443520	-3.1166801	3.2501419
H	0.5693671	-4.1347462	-0.7623028
C	5.1473470	2.1266672	3.2331800
H	3.2137022	2.1934323	4.1738823
H	1.0203188	0.6555991	1.7104245
H	1.2530102	1.2423568	3.3599641
H	1.1580548	2.3699536	2.0057129
H	6.8670175	2.0729970	1.9485242
H	6.8695017	1.7957365	-0.2464841
H	6.3012858	0.1476312	-0.5171524
H	5.4194976	1.4992301	-1.2232410
C	5.1536789	-1.8057954	-3.7598299
H	5.9447032	-3.0376154	-2.1870410
H	4.2278012	-2.9009639	0.2546281
H	5.9310019	-2.4838718	0.0319520
H	4.8265371	-1.3280570	0.7843787
H	4.2307974	-0.3592796	-5.0484626
H	1.8641289	1.2136471	-3.1235109
H	3.3763213	2.1320051	-3.1578700
H	2.8649691	1.2183661	-4.5826717
C	0.4728398	-5.0562335	1.7403492
C	5.8889620	2.6735425	4.4244193
C	5.8662057	-2.5274474	-4.8733258
C	0.3620356	-5.4939540	3.0024295
H	0.0913287	-5.6812649	0.9338425
H	5.8476598	1.9660830	5.2610248
H	6.9387699	2.8612672	4.1850279
H	5.4366028	3.6095358	4.7704392
H	6.6164019	-1.8766025	-5.3380187
H	6.3715439	-3.4235566	-4.5040882
H	5.1625829	-2.8220806	-5.6596368
H	-0.0936619	-6.4546744	3.2198551
H	0.7094868	-4.9166925	3.8550267
N	1.5064035	1.1924357	-0.7287831
N	0.1845700	0.7651605	-0.3761223
C	1.6869686	2.5732099	-0.8450822
C	-0.4003892	0.0367679	-1.2964924

O	0.8019224	3.3993415	-0.8669984
O	3.0167238	2.8298716	-0.9499770
O	-1.5667682	-0.4802474	-1.1411614
O	0.2250224	-0.2543955	-2.4647903
C	3.4080559	4.2405100	-0.8881650
C	-0.5242626	-1.0650008	-3.4240911
C	3.4698722	4.7222138	0.5495152
H	4.3866904	4.2568189	-1.3700199
H	2.6917956	4.8112761	-1.4820291
C	0.3532416	-1.2257657	-4.6485255
H	-1.4638095	-0.5545228	-3.6472759
H	-0.7562195	-2.0263419	-2.9569621
H	4.1651017	4.1146776	1.1351519
H	3.8140603	5.7619069	0.5644798
H	2.4805543	4.6803652	1.0130385
H	1.3045638	-1.7017475	-4.3913999
H	-0.1636817	-1.8562703	-5.3790888
H	0.5614734	-0.2577119	-5.1127150
B	-2.7107969	0.0481065	-0.1774515
C	-2.2159628	0.0873199	1.3655565
C	-3.1545649	1.4741640	-0.8133737
C	-3.8501120	-1.0993045	-0.2726667
C	-2.6746355	1.0589370	2.2699730
C	-1.4156380	-0.9393804	1.8981969
C	-2.4389517	2.6538165	-0.5374738
C	-4.1892312	1.5730861	-1.7611511
C	-5.1431336	-0.8664492	0.2270807
C	-3.5979916	-2.3961921	-0.7602667
C	-2.3438048	1.0252404	3.6281176
H	-3.3028528	1.8670770	1.9007098
C	-1.0835501	-0.9905594	3.2505861
H	-1.0468773	-1.7185398	1.2349217
C	-2.7354228	3.8624388	-1.1704554
H	-1.6077178	2.6112307	0.1585131
C	-4.5049188	2.7777171	-2.3947498
H	-4.7610727	0.6807032	-2.0086232
C	-6.1237486	-1.8546472	0.2305139
H	-5.3861117	0.1174014	0.6251228
C	-4.5661631	-3.3953124	-0.7570830
H	-2.6085163	-2.6253743	-1.1478295
C	-1.5437018	-0.0034449	4.1275081
H	-2.7119079	1.8012442	4.2971241
H	-0.4631075	-1.8007706	3.6257013
C	-3.7761386	3.9328028	-2.0993513
H	-2.1437907	4.7474914	-0.9464160
H	-5.3151295	2.8168407	-3.1208161
C	-5.8593780	-3.1456413	-0.2638665
H	-7.1168686	-1.6357291	0.6214609
H	-4.3171293	-4.3827428	-1.1402840
H	-1.2832305	-0.0378297	5.1830473
H	-4.0130504	4.8734631	-2.5921042
C	-6.9254671	-4.1514180	-0.2396958
C	-6.8703755	-5.4175229	-0.6857521
H	-7.8643746	-3.8023744	0.1937298
H	-7.7335441	-6.0725318	-0.6133280
H	-5.9738687	-5.8402139	-1.1327304

6_III : type III isomer of **6**

99

Energy = -2520.547560291

P	-2.6517494	-0.4080123	0.2571952
C	-2.0292884	-2.0948083	0.1130925
C	-3.3256068	0.1254128	-1.3404678
C	-3.8907799	-0.4804285	1.5772281
C	-2.1496336	-2.7426647	-1.1292236
C	-1.6142272	-2.8252357	1.2523264
C	-2.3694574	0.1290532	-2.3726093
C	-4.6394883	0.5954835	-1.5877810
C	-4.8942648	-1.4795800	1.5545656
C	-3.8118989	0.4235503	2.6454666
C	-1.8269721	-4.0853181	-1.2700425
H	-2.5066281	-2.1943035	-1.9911994
C	-1.2495080	-4.1650696	1.0674576
C	-1.6133688	-2.2887802	2.6572472
C	-2.6978859	0.5459913	-3.6572815
H	-1.3501072	-0.1795810	-2.1633560
C	-4.9384792	0.9961514	-2.8973791
C	-5.7281016	0.7612226	-0.5576532
C	-5.7984557	-1.5128602	2.6223342
C	-5.0600312	-2.4966011	0.4516948
C	-4.7249709	0.3633829	3.6926728
H	-3.0032641	1.1399050	2.6839846
C	-1.3505616	-4.7952089	-0.1690027
H	-1.9325684	-4.5659441	-2.2372340
H	-0.8980896	-4.7250633	1.9300124
H	-0.7960855	-2.7382914	3.2291465
H	-1.5213302	-1.2054931	2.7179094
H	-2.5521678	-2.5655358	3.1541080
C	-3.9973430	0.9705002	-3.9239755
H	-1.9358838	0.5410494	-4.4290775
H	-5.9411305	1.3626302	-3.1017560
H	-6.5221594	1.3858354	-0.9742783
H	-6.1761620	-0.1956744	-0.2745241
H	-5.3576106	1.2282809	0.3563991
C	-5.7248757	-0.6081052	3.6794026
H	-6.5807470	-2.2675412	2.6127092
H	-4.3911767	-3.3514287	0.6003014
H	-6.0874505	-2.8698717	0.4463258
H	-4.8409625	-2.0840123	-0.5377462
H	-4.6488589	1.0684857	4.5146544
H	-1.0718755	-5.8402325	-0.2686183
H	-4.2759677	1.3007183	-4.9206368
H	-6.4459557	-0.6649146	4.4897985
C	0.0635169	0.5943471	2.4002711
O	-0.8625251	0.7898786	3.1894287
O	1.3525272	0.6082363	2.7692016
C	-1.6817241	2.0612245	0.7528677
O	-0.9352165	2.9065344	1.1810712
O	-2.9585402	2.2558458	0.3634883
N	-1.3551394	0.6911071	0.5890629

N	-0.0663151	0.3012991	1.0493005
C	1.6034207	0.6918947	4.1979619
C	3.0979992	0.5203476	4.3847069
H	1.0321795	-0.0967354	4.6967418
H	1.2462421	1.6610100	4.5591969
H	3.6455856	1.3296732	3.8931035
H	3.3360456	0.5372036	5.4535384
H	3.4288124	-0.4324596	3.9608399
C	-3.4089863	3.6509845	0.2908131
C	-2.9735139	4.2750005	-1.0214292
H	-4.4936856	3.5746052	0.3731010
H	-3.0051309	4.1784833	1.1568614
H	-3.3467870	3.6927485	-1.8690141
H	-3.3779675	5.2905039	-1.0884073
H	-1.8830053	4.3324465	-1.0777787
B	1.2649528	0.3109981	0.0494999
C	0.9222347	-0.2370973	-1.4622944
C	1.8714546	1.8292216	0.0484077
C	2.3054128	-0.8202121	0.6006159
C	1.0966384	0.5232890	-2.6351807
C	0.6369051	-1.6023379	-1.6642468
C	1.2433050	2.8848096	-0.6410886
C	3.0234948	2.1813447	0.7736831
C	3.6332406	-0.8354015	0.1372080
C	1.9249901	-1.9018712	1.4123582
C	0.9388469	-0.0168622	-3.9149161
C	0.4755987	-2.1580725	-2.9332809
C	1.7313540	4.1916079	-0.6308105
C	3.5281381	3.4841604	0.8014580
C	4.5358450	-1.8407100	0.4904861
C	2.8087177	-2.9210966	1.7680599
C	0.6136397	-1.3636826	-4.0733595
C	2.8871194	4.5002472	0.0899231
C	4.1297374	-2.8926096	1.3155167
H	1.3796411	1.5671330	-2.5570262
H	0.5644020	-2.2542688	-0.7990508
H	0.3265441	2.6884799	-1.1913724
H	3.5342355	1.4116155	1.3443530
H	3.9690144	-0.0402201	-0.5257667
H	0.9047679	-1.9454838	1.7808477
H	1.0761551	0.6175611	-4.7883677
H	0.2497465	-3.2166215	-3.0345483
H	1.2071775	4.9717163	-1.1799615
H	4.4219776	3.7072911	1.3815842
H	5.5579571	-1.8068630	0.1171853
H	2.4675107	-3.7384114	2.4013011
H	0.4894249	-1.7920612	-5.0650247
H	3.2761086	5.5160242	0.1027762
H	4.8278986	-3.6790354	1.5932367

6_II : type II isomer of **6**

99

Energy = -2520.554321497

P	-3.3001870	0.4605043	0.0847134
C	-4.6313611	-0.6222645	-0.4639610
C	-2.6812386	1.3190975	-1.3705910
C	-3.8992682	1.6356238	1.3135726
C	-5.8629858	-0.5289662	0.2042977
C	-4.4990723	-1.4288108	-1.6191479
C	-1.3143819	1.2008521	-1.6594772
C	-3.5583884	1.9745668	-2.2680996
C	-4.2587553	1.2133683	2.6154045
C	-3.9490266	2.9921940	0.9637082
C	-6.9679640	-1.2475386	-0.2383564
H	-5.9619716	0.1286659	1.0611268
C	-5.6285700	-2.1380538	-2.0403723
C	-3.2296177	-1.5489197	-2.4204404
C	-0.7927369	1.7351880	-2.8321422
H	-0.6651300	0.6517406	-0.9870717
C	-2.9975896	2.5239650	-3.4271142
C	-5.0543366	2.0628611	-2.0829522
C	-4.7106182	2.1904546	3.5079320
C	-4.1007889	-0.2040737	3.1019604
C	-4.3936312	3.9435597	1.8766285
H	-3.6197194	3.3059204	-0.0212501
C	-6.8465112	-2.0575655	-1.3666738
H	-7.9126445	-1.1686164	0.2901489
H	-5.5425787	-2.7720698	-2.9189877
H	-3.1935598	-0.7831585	-3.2045117
H	-2.3369872	-1.4421519	-1.8043092
H	-3.1916969	-2.5274883	-2.9064299
C	-1.6380772	2.4062252	-3.7141941
H	0.2621836	1.6055601	-3.0514713
H	-3.6523597	3.0408716	-4.1241974
H	-5.4369959	2.9707594	-2.5571040
H	-5.5470312	1.2047363	-2.5554789
H	-5.3573235	2.0733529	-1.0339476
C	-4.7850650	3.5366308	3.1506663
H	-4.9983842	1.8837350	4.5101584
H	-4.7159257	-0.3705680	3.9895588
H	-4.3782806	-0.9497469	2.3511379
H	-3.0506935	-0.3748427	3.3682743
H	-4.4266200	4.9909210	1.5943835
H	-7.6998806	-2.6249812	-1.7263555
H	-1.2453417	2.8301263	-4.6339495
H	-5.1378805	4.2667822	3.8731658
C	-0.0185880	0.5709937	1.5003131
O	0.5710804	-0.0943560	0.5718320
O	0.7522000	1.2958944	2.3192986
C	-1.9494699	-1.7237599	1.0476115
O	-2.5934088	-2.5299092	0.3950620
O	-1.0910945	-2.0135472	2.0307269
N	-2.0175743	-0.3377899	0.8520452
N	-1.3194891	0.5648936	1.7270301
C	0.0951670	2.1597807	3.2977318
C	-0.4050443	3.4399862	2.6518094
H	0.8862726	2.3573957	4.0235608
H	-0.7148261	1.5979441	3.7685499
H	-1.1946088	3.2205101	1.9297929
H	-0.8170030	4.0979619	3.4252861

H	0.4144343	3.9579046	2.1455871
C	-0.8918291	-3.4401657	2.2967736
C	-1.9000880	-3.9329004	3.3178221
H	-0.9706683	-3.9787716	1.3506964
H	0.1331321	-3.4889555	2.6666151
H	-1.8285167	-3.3545717	4.2439348
H	-2.9177853	-3.8548608	2.9249526
H	-1.6973103	-4.9841634	3.5487107
B	2.0666484	-0.0573222	0.0595923
C	2.6881439	1.4337355	0.2190117
C	2.8889133	-1.1919759	0.8725970
C	1.8842461	-0.4644377	-1.5081710
C	2.0189319	2.5494427	-0.3168869
C	3.9081447	1.6868353	0.8635921
C	4.0411370	-1.7751952	0.3145616
C	2.5265936	-1.6279526	2.1586286
C	2.6394588	0.1228637	-2.5367759
C	0.9455483	-1.4391799	-1.8961990
C	2.5209061	3.8449025	-0.2012700
C	4.4324825	2.9784396	0.9805723
C	4.7981948	-2.7292693	0.9980659
C	3.2658128	-2.5913993	2.8506095
C	2.4653175	-0.2288125	-3.8797245
C	0.7494169	-1.7906918	-3.2317780
C	3.7374831	4.0671136	0.4512603
C	4.4106446	-3.1463911	2.2743462
C	1.5109521	-1.1835313	-4.2346337
H	1.0696541	2.4025435	-0.8275192
H	4.4583082	0.8515767	1.2927300
H	4.3496582	-1.4751036	-0.6858697
H	1.6432141	-1.2082161	2.6325263
H	3.3689453	0.8889450	-2.2812462
H	0.3395817	-1.9180774	-1.1298089
H	1.9669941	4.6836530	-0.6187609
H	5.3815905	3.1361862	1.4895086
H	5.6851409	-3.1557149	0.5334759
H	2.9513107	-2.9079644	3.8435539
H	3.0649997	0.2521225	-4.6500149
H	0.0002525	-2.5347152	-3.4952305
H	4.1371973	5.0743178	0.5435708
H	4.9901583	-3.8949779	2.8093970
H	1.3600929	-1.4502229	-5.2779173

6_I : type I isomer of **6**
99
Energy = -2520.555701439

P	-2.8966815	-0.2132098	0.1027756
C	-4.6291052	0.2784737	0.1480803
C	-2.8057351	-1.8652842	0.8189054
C	-2.2699279	-0.2169117	-1.5851634
C	-5.2627559	0.5838305	-1.0663487
C	-5.3703909	0.2169255	1.3508103
C	-2.0012354	-2.0562481	1.9509748
C	-3.5824490	-2.9292076	0.3020619
C	-2.2086953	0.9761461	-2.3415028
C	-1.8152361	-1.4283436	-2.1244703
C	-6.6261218	0.8559580	-1.1050763
H	-4.6877745	0.5919105	-1.9862510
C	-6.7383887	0.4969509	1.2815998
C	-4.7627967	-0.1483723	2.6789556
C	-1.9139523	-3.3050003	2.5550192
H	-1.4629415	-1.2163723	2.3716746
C	-3.4591114	-4.1794998	0.9214207
C	-4.5717587	-2.7833167	-0.8276277
C	-1.7805375	0.8770302	-3.6691577
C	-2.4808714	2.3378462	-1.7606981
C	-1.3611845	-1.4895122	-3.4366352
H	-1.7932827	-2.3192579	-1.5082605
C	-7.3650236	0.8138973	0.0766494
H	-7.1037934	1.0938793	-2.0501413
H	-7.3209768	0.4678314	2.1987460
H	-4.7536779	-1.2367681	2.8118308
H	-3.7359092	0.2103887	2.7720955
H	-5.3527552	0.2837060	3.4913520
C	-2.6339837	-4.3758870	2.0263244
H	-1.2839033	-3.4400145	3.4287371
H	-4.0351097	-5.0114929	0.5250229
H	-4.7779402	-3.7600229	-1.2725348
H	-5.5180126	-2.3766045	-0.4520740
H	-4.2210545	-2.1154561	-1.6176226
C	-1.3686819	-0.3355962	-4.2185914
H	-1.7502463	1.7803306	-4.2723973
H	-3.3032306	2.3466886	-1.0396211
H	-2.7159603	3.0517357	-2.5542447
H	-1.5750181	2.6887029	-1.2506890
H	-0.9969467	-2.4295542	-3.8351350
H	-8.4303109	1.0251138	0.0624926
H	-2.5635869	-5.3604955	2.4792202
H	-1.0317248	-0.3724270	-5.2505748
C	0.2778707	0.3233390	1.6419449
O	1.5259671	0.0366546	1.5018921
O	-0.1622613	0.4958610	2.9095291
C	-2.2063450	2.1105835	1.3917657
O	-3.3479850	2.4865917	1.6083815
O	-1.0975701	2.8502881	1.4749638
N	-1.8824488	0.7989792	1.0078179
N	-0.5364451	0.4444614	0.6206186
C	0.8292839	0.4039174	3.9779410
C	0.0803422	0.5897023	5.2814053
H	1.5816028	1.1803611	3.8153195
H	1.3180427	-0.5720731	3.9193016
H	-0.6720109	-0.1941364	5.4140305
H	0.7869283	0.5358680	6.1158582
H	-0.4188411	1.5626026	5.3111595
C	-1.2709845	4.2259371	1.9389919
C	-1.1743350	4.2908468	3.4520847
H	-0.4536555	4.7601435	1.4521433
H	-2.2306894	4.5962044	1.5729051
H	-1.9860267	3.7243985	3.9174905
H	-0.2163947	3.8895208	3.7952189

H	-1.2500285	5.3346760	3.7751008
B	2.2314823	-0.0758078	0.0957154
C	1.7265015	-1.4271073	-0.6456156
C	1.9562439	1.3194624	-0.6891813
C	3.8038900	-0.2478355	0.4705902
C	0.9237958	-2.3885135	-0.0101498
C	2.1477634	-1.7369790	-1.9521858
C	2.3691953	2.5226168	-0.0881381
C	1.2958196	1.4271267	-1.9212186
C	4.8099303	0.1446875	-0.4296884
C	4.2307427	-0.8507772	1.6668711
C	0.5395038	-3.5760513	-0.6370868
C	1.7736863	-2.9182771	-2.5949145
C	2.1226660	3.7675424	-0.6673107
C	1.0619261	2.6644158	-2.5269831
C	6.1657735	-0.0582340	-0.1626952
C	5.5834491	-1.0516472	1.9536042
C	0.9600720	-3.8473500	-1.9406343
C	1.4680547	3.8438313	-1.8998418
C	6.5613111	-0.6584450	1.0357860
H	0.5815193	-2.2071557	1.0044487
H	2.7914702	-1.0361113	-2.4805171
H	2.8873472	2.4769867	0.8687884
H	0.9281676	0.5253074	-2.4033269
H	4.5216728	0.6326125	-1.3592470
H	3.4839864	-1.1660788	2.3923448
H	-0.0957663	-4.2849225	-0.1091969
H	2.1138708	-3.1151850	-3.6097874
H	2.4431435	4.6783612	-0.1646067
H	0.5436618	2.7111306	-3.4823956
H	6.9163573	0.2590826	-0.8842205
H	5.8777744	-1.5163637	2.8930303
H	0.6593052	-4.7657585	-2.4393522
H	1.2760181	4.8088602	-2.3633748
H	7.6155010	-0.8137096	1.2531141

Aa : P-N adduct of EN=NE and PTo_3
65
Energy = -1800.252112857

P	-0.5456839	-0.1220532	-0.0404502
C	-1.9955806	0.9272813	-0.3008988
C	-1.0931892	-1.3822380	1.1408028
C	0.0056050	-0.8957454	-1.5693423
C	-2.5379478	1.0083429	-1.5919791
C	-2.6448432	1.5532646	0.7867899
C	-0.4042785	-1.5111914	2.3572839
C	-2.2541191	-2.1521420	0.8902132
C	0.4077303	-0.1314811	-2.6862839
C	0.1200284	-2.2967545	-1.5800765
C	-3.7031433	1.7287665	-1.8308486
H	-2.0548071	0.4882248	-2.4114898
C	-3.8127394	2.2748137	0.5183325
C	-2.1633019	1.4603039	2.2115965
C	-0.8500698	-2.3977206	3.3314395
H	0.4981395	-0.9322555	2.5105780
C	-2.6672327	-3.0486102	1.8850154
C	-3.0865090	-2.0505459	-0.3643011
C	0.8646363	-0.8278433	-3.8133447
C	0.4119284	1.3752842	-2.7231298
C	0.5937486	-2.9581420	-2.7051480
H	-0.1496087	-2.8602970	-0.6928555
C	-4.3398290	2.3701268	-0.7687521
H	-4.1088521	1.7841922	-2.8361363
H	-4.3154006	2.7737782	1.3430954
H	-2.6331888	0.6081203	2.7174054
H	-1.0829671	1.3280355	2.2823940
H	-2.4348651	2.3667286	2.7590492
C	-1.9848993	-3.1732107	3.0924264
H	-0.3062647	-2.4879480	4.2669148
H	-3.5494276	-3.6558703	1.6988093
H	-3.6726273	-2.9639195	-0.4954252
H	-3.7842305	-1.2082120	-0.2984378
H	-2.4802376	-1.9022052	-1.2613128
C	0.9570932	-2.2160003	-3.8315578
H	1.1727309	-0.2565238	-4.6855526
H	0.0337148	1.8322551	-1.8071041
H	-0.1875459	1.7400125	-3.5652415
H	1.4407124	1.7196015	-2.8546758
H	0.6837485	-4.0398618	-2.7019239
H	-5.2483177	2.9405713	-0.9392116
H	-2.3372528	-3.8763790	3.8418208
H	1.3262074	-2.7200199	-4.7203809
C	2.7327552	-0.2112731	-0.1342558
O	2.8404761	0.6125119	-1.0522189
O	3.6523360	-1.2290291	0.0350903
C	0.9481233	2.0426966	0.7598228
O	0.1322082	2.8729395	0.3793778
O	2.1041727	2.3128789	1.3858826
N	0.7718261	0.6698463	0.6356928
N	1.8007043	-0.3144756	0.8459445
C	4.6680309	-1.2967541	-0.9899633
C	5.5426401	-2.4958395	-0.6740635
H	5.2442477	-0.3648160	-0.9938378
H	4.1810498	-1.3971802	-1.9663829
H	4.9504382	-3.4166532	-0.6697464
H	6.3273648	-2.5908599	-1.4325346
H	6.0199288	-2.3842316	0.3050060
C	2.3905899	3.7346433	1.5749381
C	1.7288630	4.2567364	2.8381715
H	3.4791195	3.7700169	1.6415783
H	2.0551876	4.2744771	0.6869676
H	0.6399118	4.2037108	2.7537010
H	2.0500213	3.6788849	3.7101806
H	2.0148317	5.3031342	2.9915920

Ab : P-N adduct of EN=NE and $PMes_3$
83
Energy = -2036.270173101

P	-0.5424769	-0.2255854	-0.0432571
C	-2.0491630	0.7911475	-0.3629897

C	-1.1286861	-1.3527531	1.2821047
C	0.1621611	-1.0256447	-1.5183026
C	-2.7812137	0.8157432	-1.5829895
C	-2.5420375	1.5448339	0.7395538
C	-0.6380536	-1.3896122	2.6191684
C	-2.2542556	-2.1525588	0.9400567
C	0.4956143	-0.1717876	-2.6023851
C	0.5431994	-2.3925800	-1.5545090
C	-3.9097786	1.6405430	-1.6736807
C	-3.6676544	2.3511137	0.5803283
C	-1.9549142	1.5177737	2.1260885
C	-1.2973558	-2.2151626	3.5407138
C	-2.8598355	-2.9540279	1.9073460
C	-2.8506714	-2.2252998	-0.4440728
C	1.0809954	-0.7281798	-3.7405711
C	0.3356032	1.3292766	-2.5917467
C	1.1294237	-2.8867323	-2.7217674
C	-4.3627932	2.4342265	-0.6249856
H	-4.4575821	1.6418381	-2.6140169
H	-4.0079881	2.9294960	1.4367677
H	-2.4446184	0.7452796	2.7303422
H	-0.8879182	1.3150992	2.1363291
H	-2.1151512	2.4831803	2.6122969
C	-2.4016865	-3.0010724	3.2222526
H	-0.9164342	-2.2329554	4.5593922
H	-3.7107781	-3.5635760	1.6102767
H	-3.3489051	-3.1892488	-0.5769923
H	-3.5948054	-1.4372947	-0.5965299
H	-2.0993626	-2.1317409	-1.2309360
C	1.3852013	-2.0846328	-3.8354499
H	1.3248074	-0.0643477	-4.5675563
H	-0.3871475	1.7028265	-1.8662686
H	0.0489296	1.6849118	-3.5859431
H	1.3037960	1.7640316	-2.3261989
H	1.4272150	-3.9328543	-2.7411432
C	2.7822272	-0.1783112	-0.3230724
O	2.9707688	0.7391280	-1.1306777
O	3.6285097	-1.2728269	-0.2652253
C	0.9613840	2.0176667	0.6705932
O	0.1349246	2.8555737	0.3347377
O	2.1265458	2.2765921	1.2912476
N	0.7996074	0.6511719	0.4998242
N	1.8337951	-0.3362302	0.6295233
C	4.6187549	-1.3221632	-1.3173619
C	5.2618590	-2.6951587	-1.2600216
H	5.3533937	-0.5230900	-1.1641799
H	4.1217926	-1.1484856	-2.2770377
H	4.5134500	-3.4767972	-1.4263639
H	6.0293328	-2.7767833	-2.0374825
H	5.7358434	-2.8666230	-0.2880609
C	2.4129012	3.6927556	1.5046958
C	1.7760449	4.1839092	2.7933977
H	3.5025804	3.7325002	1.5491466
H	2.0566478	4.2523761	0.6372140
H	0.6858586	4.1266564	2.7300622
H	2.1173761	3.5874724	3.6451413
H	2.0602225	5.2279709	2.9653750
C	0.5388697	-0.6304154	3.1772345
H	1.4252644	-0.7606505	2.5503591
H	0.3506583	0.4469398	3.2332571
H	0.7351671	-0.9803914	4.1937449
C	-3.0528243	-3.8914912	4.2483916
H	-4.1299108	-3.9740809	4.0734701
H	-2.6326949	-4.9041925	4.1984470
H	-2.8871957	-3.5138414	5.2615061
C	0.4683857	-3.3323530	-0.3789749
H	0.9245424	-2.8636474	0.4978452
H	-0.5545702	-3.6126874	-0.1151501
H	1.0165952	-4.2470916	-0.6185325
C	1.9949302	-2.6614755	-5.0868202
H	1.2166820	-2.8796275	-5.8293922
H	2.6958230	-1.9570139	-5.5457178
H	2.5224642	-3.5959908	-4.8748206
C	-2.5224633	0.0163516	-2.8428099
H	-2.0684933	0.6511256	-3.6108484
H	-1.8774639	-0.8445705	-2.7082199
H	-3.4830938	-0.3306761	-3.2352052
C	-5.5509768	3.3458147	-0.7851222
H	-5.2241654	4.3463895	-1.0960706
H	-6.2382694	2.9702873	-1.5488768
H	-6.0949962	3.4561890	0.1578212

Ac : P-N adduct of EN=NE and PPh_3
56
Energy = -1682.233970485

P	-2.6282754	0.0257195	0.5121184
C	-1.6801988	-1.4990762	0.5515679
C	-3.4444367	0.1352141	-1.0959254
C	-3.8646568	-0.0503662	1.8141787
C	-1.1130193	-2.0039262	-0.6282005
C	-1.5454044	-2.2041768	1.7564288
C	-2.6914836	0.5809996	-2.1938342
C	-4.7908939	-0.2069416	-1.2651091
C	-4.6647602	-1.2002777	1.9159847
C	-4.0336711	1.0074436	2.7141030
C	-0.3999721	-3.1987674	-0.5961591
H	-1.2294332	-1.4720624	-1.5661744
C	-0.8400622	-3.4049932	1.7752085
H	-1.9706099	-1.7985729	2.6667466
C	-3.2835072	0.6707205	-3.4509209
H	-1.6551143	0.8770218	-2.0595021
C	-5.3765575	-0.1156553	-2.5270583
H	-5.3883022	-0.5195785	-0.4156173
C	-5.6454260	-1.2756788	2.9034355
H	-4.5128438	-2.0377857	1.2413278
C	-5.0162823	0.9235503	3.6971280
H	-3.3820042	1.8700315	2.6626353
C	-0.2653436	-3.9009249	0.6040682
H	0.0432522	-3.5848359	-1.5088947
H	-0.7360391	-3.9519271	2.7072047

C	-4.6254934	0.3206518	-3.6195261
H	-2.6991870	1.0226757	-4.2955775
H	-6.4229877	-0.3766784	-2.6528208
C	-5.8251585	-0.2117406	3.7893638
H	-6.2614232	-2.1661609	2.9825294
H	-5.1435003	1.7417714	4.3994414
H	0.2834112	-4.8380078	0.6241293
H	-5.0870090	0.3973458	-4.5995171
H	-6.5882070	-0.2722622	4.5598875
N	-1.5303661	1.3076655	0.6011888
C	-1.9275135	2.6264177	0.4098325
N	-0.1564489	1.0486542	0.8955048
O	-1.1943525	3.5953225	0.4223292
O	-3.2646349	2.6548923	0.1882290
C	-0.0528014	0.6957186	2.1892892
C	-3.8396367	3.9455561	-0.1881743
O	-0.9557871	0.5046515	3.0293801
O	1.2827437	0.5320619	2.5068084
C	-5.2865991	3.6878441	-0.5522680
H	-3.7373859	4.6220907	0.6646908
H	-3.2622720	4.3429782	-1.0268194
C	1.5613968	0.1043384	3.8621170
H	-5.8304739	3.2573808	0.2939723
H	-5.7626585	4.6346513	-0.8260292
H	-5.3572529	3.0019793	-1.4013523
C	1.5230787	-1.4104150	3.9955116
H	0.8432965	0.5765199	4.5374480
H	2.5631594	0.4926335	4.0660647
H	2.1999015	-1.8803845	3.2745137
H	1.8351495	-1.7008719	5.0057156
H	0.5108462	-1.7825721	3.8218324

Ad : EN=NE adduct of $PMes_2(C_6H_4CH=CH_2)$
78
Energy = -1995.706505172

P	-0.5648372	-0.2754645	-0.0418784
C	-1.9448267	0.8589496	-0.3876401
C	-1.1661335	-1.3733544	1.2843674
C	0.0035529	-1.3505984	-1.3776129
C	-2.4816662	1.0734992	-1.6819310
C	-2.5331642	1.5076720	0.7303653
C	-0.5185288	-1.5557452	2.5345078
C	-2.3813986	-2.0620566	1.0129590
C	0.8245423	-0.7957533	-2.3757176
C	-0.2574118	-2.7237409	-1.3930100
C	-3.5890767	1.9162802	-1.8148837
C	-3.6330026	2.3409337	0.5313603
C	-2.0412859	1.3731238	2.1503975
C	-1.0793538	-2.4592263	3.4447424
C	-2.8843868	-2.9517709	1.9641366
C	-3.2199290	-1.8770365	-0.2343206
C	1.3370690	-1.5931114	-3.3817694
C	0.2618128	-3.5202403	-2.4098158
C	-4.1829230	2.5617418	-0.7310655
H	-3.9994027	2.0660595	-2.8110365
H	-4.0647668	2.8378607	1.3976531
H	-2.5019356	0.5152869	2.6515252
H	-0.9590830	1.2493815	2.2137914
H	-2.3029524	2.2738853	2.7114754
C	-2.2458326	-3.1766083	3.1837706
H	-0.5781094	-2.5932795	4.4007843
H	-3.8080159	-3.4799473	1.7366978
H	-3.8275018	-2.7709825	-0.3989249
H	-3.8958119	-1.0228521	-0.1189152
H	-2.6318863	-1.7065808	-1.1380923
C	1.0648017	-2.9753009	-3.4243817
H	1.9696525	-1.1403096	-4.1381015
H	0.0549111	-4.5872123	-2.4108124
C	2.9355381	0.2195484	-0.4307709
O	2.9315223	1.2319421	-1.1543016
O	4.0435875	-0.6074622	-0.3500967
C	0.8956051	1.9662434	0.6059013
O	0.1084809	2.8021720	0.1951187
O	1.9291886	2.2086916	1.4271973
N	0.8268013	0.5920351	0.3080002
N	1.9827280	-0.2613303	0.3903660
C	5.1501089	-0.2508516	-1.2114645
C	4.9624565	-0.7798570	-2.6267286
H	6.0204773	-0.7087499	-0.7330697
H	5.2644524	0.8365627	-1.2127786
H	4.1155833	-0.2845903	-3.1074826
H	5.8647874	-0.5843436	-3.2182353
H	4.7812961	-1.8597066	-2.6160777
C	2.1712033	3.6179634	1.7229025
C	1.3179900	4.0800631	2.8917919
H	3.2360651	3.6530442	1.9591068
H	1.9721954	4.1990643	0.8198446
H	0.2561474	4.0333207	2.6356396
H	1.5025213	3.4595868	3.7743229
H	1.5712936	5.1169024	3.1389768
C	0.7092482	-0.8136976	2.9909286
H	1.5278884	-0.8804977	2.2652709
H	0.5029373	0.2553077	3.1223325
H	1.0297708	-1.2079034	3.9586692
C	-2.7921765	-4.1686518	4.1773601
H	-3.8857877	-4.1954325	4.1492681
H	-2.4321614	-5.1789769	3.9447198
H	-2.4699901	-3.9278236	5.1944443
C	-1.9636130	0.4548324	-2.9562583
H	-0.9697851	0.8328202	-3.2118109
H	-1.8890454	-0.6342243	-2.8968135
H	-2.6393151	0.7041749	-3.7777125
C	-5.3544854	3.4894024	-0.9205421
H	-5.0072802	4.5253891	-1.0243935
H	-5.9176110	3.2365512	-1.8234478
H	-6.0298427	3.4546164	-0.0602799
H	1.0887814	0.2564653	-2.3335326
H	-0.8435617	-3.1808309	-0.6035745
C	1.6015550	-3.8635733	-4.4597917
C	2.4302503	-3.5190209	-5.4565030

H	1.2843299	-4.9030163	-4.3798755
H	2.7760467	-4.2575794	-6.1728926
H	2.7918199	-2.5033207	-5.5921517

Ba : B-N adduct of EN=NE and $B(C_6F_5)_3$

56

Energy = -2854.953650346

C	-0.3397123	-1.6685348	2.4381402
O	-0.6403106	-1.7756851	3.5966597
O	0.0068335	-2.5490210	1.5473214
C	-1.5609249	0.4957889	3.6486227
O	-2.7415041	0.2405050	3.5637800
O	-0.8446370	0.9188367	4.6660145
N	-0.8028337	0.6659394	2.4199564
N	-0.3188940	-0.2814139	1.7875985
C	0.1428391	-3.9732808	1.9723636
C	1.5843029	-4.3862163	1.7796602
H	-0.5412621	-4.5057430	1.3109219
H	-0.1966267	-4.0427802	3.0054391
H	2.2465512	-3.8330544	2.4504547
H	1.6684373	-5.4523855	2.0128500
H	1.8993849	-4.2316609	0.7445604
C	-1.5611856	1.0506073	5.9629070
C	-2.2143430	2.4131096	6.0608959
H	-2.2786906	0.2315658	6.0277424
H	-0.7650901	0.9123125	6.6933774
H	-1.4736286	3.2093937	5.9466705
H	-2.9955888	2.5329554	5.3054411
H	-2.6747311	2.5081024	7.0500479
B	0.1942557	-0.0440378	0.2420170
C	1.7148105	-0.5948023	0.0504976
C	-0.9982170	-0.8476672	-0.5399630
C	0.2624284	1.5610464	-0.0393781
C	2.6055992	-0.9445354	1.0611670
C	2.2655186	-0.5915319	-1.2332177
C	-2.3123119	-0.3646015	-0.4765124
C	-0.8591696	-2.0586802	-1.2260306
C	-0.2540844	2.2004524	-1.1689592
C	1.0072461	2.3920083	0.8036057
C	3.9284495	-1.3142032	0.8324995
C	3.5786116	-0.9493979	-1.5126847
C	-3.4028015	-0.9909995	-1.0702217
C	-1.9217004	-2.7156796	-1.8385998
C	-0.0873519	3.5600857	-1.4243713
C	1.1911110	3.7531561	0.5941131
C	4.4207411	-1.3192075	-0.4672511
C	-3.2044001	-2.1792371	-1.7635908
C	0.6357464	4.3458945	-0.5352471
F	2.2095198	-0.9424978	2.3656237
F	1.4929079	-0.2253125	-2.2836685
F	-2.5713322	0.7880591	0.1829588
F	0.3396603	-2.6753622	-1.3251075
F	-0.9521295	1.5140802	-2.1041301
F	1.6101309	1.8669131	1.9006570
F	4.7306797	-1.6610822	1.8570095
F	4.0430861	-0.9405968	-2.7757494
F	-4.6353036	-0.4609994	-0.9818552
F	-1.7225866	-3.8722570	-2.4965516
F	-0.6165245	4.1181390	-2.5300369
F	1.9084032	4.4958266	1.4589536
F	5.6935320	-1.6693401	-0.7113270
F	-4.2339981	-2.8037017	-2.3507818
F	0.8046056	5.6572800	-0.7674114

Bc : B-N adduct of EN=NE and BPh_3

56

Energy = -1365.691640335

C	-0.0397466	-1.4918417	2.4142128
O	0.3066642	-1.4395542	3.5683451
O	-0.1951282	-2.5086443	1.6141949
C	-1.7411660	0.2797920	3.4780043
O	-2.5954262	-0.5819869	3.4823765
O	-1.4040608	1.1225029	4.4349182
N	-1.0485617	0.6553928	2.2625109
N	-0.3619208	-0.2052757	1.6727259
C	0.2112415	-3.8392927	2.1368024
C	1.6891559	-4.0564282	1.8898804
H	-0.4174178	-4.5211757	1.5656560
H	-0.0545261	-3.8736552	3.1942858
H	2.2944607	-3.3563317	2.4699825
H	1.9439406	-5.0764002	2.1959266
H	1.9293611	-3.9352971	0.8304611
C	-2.1010186	0.9288853	5.7260134
C	-1.5288672	1.9416823	6.6902816
H	-3.1678265	1.0734766	5.5416052
H	-1.9240269	-0.1021060	6.0405170
H	-0.4554785	1.7863733	6.8298896
H	-1.7001987	2.9614022	6.3345643
H	-2.0253954	1.8236176	7.6585402
B	0.1702538	0.0219841	0.1580059
C	1.6549424	-0.5990626	0.0610336
C	-1.0411447	-0.7572351	-0.6332362
C	0.2239797	1.6083067	-0.1555812
C	2.5443130	-0.5444479	1.1508770
C	2.1722886	-1.0981803	-1.1474374
C	-2.3058277	-0.1467250	-0.6829680
C	-0.8987469	-2.0034210	-1.2639493
C	-0.1849190	2.1147540	-1.3992065
C	0.8043159	2.5212265	0.7432992
C	3.8580396	-1.0078632	1.0619634
C	3.4862810	-1.5552447	-1.2532100
C	-3.3674820	-0.7166810	-1.3845903
C	-1.9520712	-2.5802454	-1.9748700
C	-0.0349247	3.4640734	-1.7311981
C	0.9482993	3.8725593	0.4302194
C	4.3329348	-1.5285690	-0.1417130
C	-3.1896971	-1.9362945	-2.0426236
C	0.5274141	4.3521211	-0.8133877
H	2.2177386	-0.1176793	2.0978238
H	1.5405787	-1.1168697	-2.0317549

H -2.4582410 0.8007976 -0.1700532
H 0.0459620 -2.5329799 -1.1996475
H -0.6326094 1.4405578 -2.1254595
H 1.1548939 2.1768789 1.7138329
H 4.5099953 -0.9580204 1.9305798
H 3.8517817 -1.9329282 -2.2050963
H -4.3316086 -0.2156517 -1.4135332
H -1.8092383 -3.5377495 -2.4696258
H -0.3611213 3.8216122 -2.7050759
H 1.3941023 4.5518984 1.1529317
H 5.3531045 -1.8948868 -0.2186794
H -4.0130732 -2.3887977 -2.5888600
H 0.6389451 5.4041783 -1.0626228

Bf_3PTo_3 : B-P adduct of $B(C_6F_5)_3$ and PTo_3
77
Energy = -3364.333558916
P -0.6417551 1.4177386 0.3539639
C -1.0492537 1.6050358 2.1317084
C -2.4023886 1.4588339 -0.2434102
C 0.3018748 2.7622179 -0.4350231
C -0.8181395 0.5093695 2.9742193
C -1.7770945 2.7182316 2.6121976
C -3.1842066 0.5029160 0.4387306
C -3.0445901 2.3286287 -1.1585381
C 1.0190433 3.8169356 0.1665948
C 0.3701030 2.5787133 -1.8315732
C -1.2541846 0.5118078 4.2957670
H -0.3015758 -0.3626500 2.5944016
C -2.1812335 2.7092798 3.9519093
C -2.1758734 3.8871158 1.7518035
C -4.5405368 0.3363633 0.1968881
H -2.7192187 -0.1177105 1.1957960
C -4.4170948 2.1286537 -1.3910504
C -2.4340386 3.5047894 -1.8826536
C 1.7328314 4.6809939 -0.6794009
C 1.1149212 4.0833816 1.6480632
C 1.0927931 3.4415218 -2.6422363
H -0.1621759 1.7469758 -2.2835008
C -1.9285402 1.6258241 4.7914043
H -1.0633404 -0.3491256 4.9289761
H -2.7186554 3.5729768 4.3355800
H -2.2085034 4.8046240 2.3455297
H -1.4945633 4.0436755 0.9136644
H -3.1753160 3.7203821 1.3335997
C -5.1622879 1.1470265 -0.7496019
H -5.0985438 -0.4157852 0.7457085
H -4.9110128 2.7973344 -2.0913621
H -1.8994581 3.1950528 -2.7854975
H -3.2356041 4.1811122 -2.1908697
H -1.7313527 4.0638023 -1.2644762
C 1.7758986 4.5085941 -2.0581410
H 2.2836997 5.5010859 -0.2252805
H 0.6695081 5.0546352 1.8906979
H 0.6309916 3.3219237 2.2531565
H 2.1689734 4.1266190 1.9408686
H 1.1239257 3.2797057 -3.7150373
H -2.2646699 1.6506060 5.8238339
H -6.2212214 1.0391250 -0.9646592
H 2.3489952 5.1961489 -2.6731034
B 0.3604208 -0.4594874 -0.0971674
C 1.4541267 -0.9255187 1.0393073
C -0.9322168 -1.4320095 -0.2668232
C 1.3169111 -0.3402820 -1.4378064
C 2.1238986 -0.1277005 1.9661129
C 1.9447015 -2.2360347 0.9636903
C -1.7665576 -1.3047808 -1.3837421
C -1.3771580 -2.3753830 0.6636745
C 1.2727972 -1.1915684 -2.5532841
C 2.4240249 0.5230541 -1.4548437
C 3.1466267 -0.5849003 2.7943067
C 2.9609193 -2.7380669 1.7668866
C -2.9054340 -2.0646990 -1.6061931
C -2.5200119 -3.1532794 0.4913580
C 2.1588047 -1.1158477 -3.6268610
C 3.3288284 0.6337490 -2.5040092
C 3.5687490 -1.9043211 2.7003381
C -3.2902029 -3.0029480 -0.6554430
C 3.1882789 -0.1845374 -3.6166153
F 1.8063962 1.1812430 2.1074404
F 1.4318480 -3.0936765 0.0476541
F -1.4867567 -0.3654310 -2.3228289
F -0.7118781 -2.5808516 1.8293530
F 0.3759541 -2.2011147 -2.6496068
F 2.6885051 1.3105318 -0.3885561
F 3.7350775 0.2475329 3.6762618
F 3.3673898 -4.0170906 1.6460325
F -3.6593026 -1.8750938 -2.7053004
F -2.8861556 -4.0488356 1.4293959
F 2.0356497 -1.9608815 -4.6700266
F 4.3367976 1.5257682 -2.4474321
F 4.5544373 -2.3656820 3.4882076
F -4.4009898 -3.7371867 -0.8319683
F 4.0462492 -0.0981248 -4.6462181

Bf_3 : Lewis acidic borane $B(C_6F_5)_3$
34
Energy = -2209.516666700
B -0.0001185 0.0008677 0.0001180
C -0.0000251 1.5635825 -0.0000613
C 1.3524825 -0.7826789 -0.0032034
C -1.3526558 -0.7827436 0.0034840
C -0.9400024 2.3142300 0.7232154
C 0.9401326 2.3137930 -0.7235636
C 2.4660378 -0.3610241 0.7387457
C 1.5356018 -1.9560748 -0.7502415
C -1.5351825 -1.9569578 0.7494483
C -2.4667569 -0.3604755 -0.7373566
C -0.9483292 3.7025844 0.7446520
C 0.9486362 3.7021468 -0.7455869

C	3.6713166	-1.0501895	0.7558010
C	2.7336840	-2.6576053	-0.7793713
C	-2.7331386	-2.6587074	0.7785483
C	-3.6720062	-1.0497002	-0.7542100
C	0.0001913	4.4002103	-0.0006298
C	3.8065341	-2.2031696	-0.0150905
C	-3.8065721	-2.2035164	0.0155415
F	-1.8719039	1.6896872	1.4731158
F	1.8720534	1.6888280	-1.4730603
F	2.3843906	0.7427128	1.5111550
F	0.5324522	-2.4323071	-1.5172833
F	-0.5315345	-2.4339255	1.5153371
F	-2.3857257	0.7438532	-1.5089154
F	-1.8541446	4.3776839	1.4697240
F	1.8545142	4.3768166	-1.4709787
F	4.7029319	-0.6216728	1.5001915
F	2.8704323	-3.7619429	-1.5302561
F	-2.8692568	-3.7638736	1.5282956
F	-4.7042734	-0.6203397	-1.4971861
F	0.0002614	5.7362863	-0.0009399
F	4.9633891	-2.8718628	-0.0210318
F	-4.9633464	-2.8723049	0.0215666

BPh_3 : less Lewis acidic borane $B(C_6H_5)_3$
34
Energy = -720.2700720885

B	-0.0000050	-0.0017058	-0.0000418
C	-0.0000061	1.5618777	-0.0000352
C	1.3541607	-0.7833313	-0.0013426
C	-1.3541507	-0.7833596	0.0012717
C	-1.0203499	2.2977646	0.6406186
C	1.0203453	2.2977523	-0.6406924
C	2.5027955	-0.2664262	0.6362244
C	1.4802572	-2.0358777	-0.6403492
C	-1.4801711	-2.0359612	0.6401853
C	-2.5028542	-0.2664142	-0.6361431
C	-1.0157364	3.6915848	0.6560781
C	1.0157396	3.6915721	-0.6561675
C	3.7077670	-0.9670219	0.6503836
C	2.6897706	-2.7285652	-0.6571691
C	-2.6896979	-2.7286195	0.6571493
C	-3.7078443	-0.9669781	-0.6501580
C	0.0000025	4.3920787	-0.0000498
C	3.8055237	-2.1978702	-0.0041656
C	-3.8055279	-2.1978704	0.0043237
H	-1.8186838	1.7631588	1.1492217
H	1.8186790	1.7631350	-1.1492837
H	2.4397869	0.6930387	1.1434435
H	0.6172215	-2.4608641	-1.1466769
H	-0.6170521	-2.4610388	1.1462907
H	-2.4398799	0.6930453	-1.1433768
H	-1.8038707	4.2334856	1.1722567
H	1.8038788	4.2334639	-1.1723483
H	4.5719919	-0.5545292	1.1643894
H	2.7641400	-3.6828779	-1.1719437
H	-2.7640162	-3.6829443	1.1719060
H	-4.5721483	-0.5544174	-1.1639764
H	0.0000084	5.4788922	-0.0000595
H	4.7468987	-2.7409831	-0.0054036
H	-4.7469034	-2.7409830	0.0056135

EN=NE : diethyl azodicarboxylate (DEAD)
22
Energy = -645.4097373578

C	-1.8331565	0.5720303	-0.4688338
O	-2.4401080	1.6071516	-0.3036265
O	-2.2963665	-0.6653420	-0.5362067
C	1.7217409	0.4985972	-0.0269512
O	2.2759441	1.5458440	0.2240967
O	2.2469837	-0.6503459	-0.4215445
N	0.3021510	0.3324445	0.2209018
N	-0.4128667	0.5986230	-0.7637121
C	-3.7633149	-0.8214532	-0.3841348
C	-4.1278893	-0.9404128	1.0817715
H	-3.9739270	-1.7332182	-0.9427018
H	-4.2376460	0.0356396	-0.8651756
H	-3.8895498	-0.0201832	1.6221039
H	-5.2055029	-1.1189197	1.1623128
H	-3.6021641	-1.7790578	1.5471135
C	3.7256975	-0.6875178	-0.5373807
C	4.3480778	-1.0212218	0.8030600
H	3.8934238	-1.4652659	-1.2820223
H	4.0522257	0.2795518	-0.9225148
H	4.1446813	-0.2372371	1.5377781
H	3.9722569	-1.9764646	1.1805235
H	5.4330664	-1.1009109	0.6761242

jb : borane $BPh_2(C_6H_4CH{=}CH_2)$
38
Energy = -797.7191730153

B	-0.0006219	0.0068746	-0.0072887
C	0.0086883	1.5708200	-0.0040557
C	1.3498941	-0.7817341	-0.0007784
C	-1.3570589	-0.7666191	-0.0154541
C	-1.0077501	2.3121049	0.6365773
C	1.0355744	2.3015043	-0.6401604
C	2.4948460	-0.2738592	0.6505441
C	1.4768686	-2.0306260	-0.6467515
C	-1.4952752	-2.0258637	0.6051788
C	-2.5104946	-0.2384790	-0.6415750
C	-0.9938471	3.7058514	0.6555400
C	1.0404738	3.6954080	-0.6520576
C	3.6963920	-0.9801630	0.6723158
C	2.6832864	-2.7289615	-0.6557753
C	-2.7089414	-2.7050973	0.6153595
C	-3.7165362	-0.9243194	-0.6620313
C	0.0279828	4.4012306	0.0034005
C	3.7948470	-2.2078682	0.0119232
C	-3.8436814	-2.1740868	-0.0246778
H	-1.8105906	1.7820472	1.1429877

H	1.8319529	1.7628715	-1.1476542
H	2.4315995	0.6831949	1.1623434
H	0.6174118	-2.4483613	-1.1651414
H	-0.6377772	-2.4686470	1.1053770
H	-2.4462060	0.7261970	-1.1385611
H	-1.7794616	4.2516472	1.1715035
H	1.8337487	4.2331795	-1.1647266
H	4.5576742	-0.5742282	1.1964394
H	2.7584543	-3.6800190	-1.1764771
H	-2.7892558	-3.6660298	1.1190704
H	-4.5708545	-0.4859305	-1.1704505
H	0.0354171	5.4880304	0.0063035
H	4.7334127	-2.7558269	0.0174536
C	-5.0949394	-2.9389677	0.0019048
C	-6.2596400	-2.5971267	-0.5697873
H	-5.0419665	-3.8799133	0.5497931
H	-7.1296120	-3.2412726	-0.4874693
H	-6.3866599	-1.6764573	-1.1332433

jp : phosphine $PMes_2(C_6H_4CH{=}CH_2)$
56
Energy = -1350.264117604

P	2.2301830	-0.5004601	-0.1888830
C	1.9751625	-1.7837973	1.0877216
C	3.1111972	0.8245146	0.7390422
C	3.4199825	-1.0627609	-1.4788182
C	2.5419117	-1.7295035	2.3680547
C	1.0324962	-2.7970735	0.8231483
C	2.3088297	1.9149011	1.1601604
C	4.4967058	0.8402541	1.0185600
C	4.0244439	-2.3362267	-1.6149933
C	3.6447422	-0.0888317	-2.4912071
C	2.2125718	-2.6802414	3.3310662
H	3.2455358	-0.9391262	2.6117711
C	0.7159574	-3.7519878	1.7772250
H	0.5464487	-2.8318334	-0.1492846
C	2.9007765	2.9932000	1.8205287
C	0.8161993	1.9459470	0.9253581
C	5.0473754	1.9468833	1.6774102
C	5.4240815	-0.2936932	0.6590736
C	4.8346487	-2.5885209	-2.7296861
C	3.8747353	-3.4617563	-0.6202074
C	4.4703360	-0.3889109	-3.5765674
C	3.0290805	1.2887524	-2.4303993
C	1.3052613	-3.7177180	3.0566425
H	2.6694119	-2.6258184	4.3169096
H	-0.0077137	-4.5243942	1.5326934
C	4.2736143	3.0345955	2.0829649
H	2.2710547	3.8235623	2.1356595
H	0.3161604	1.0993505	1.4098144
H	0.3900459	2.8699187	1.3268755
H	0.5785212	1.8835763	-0.1423084
H	6.1158851	1.9479750	1.8869219
H	6.3411456	-0.2284646	1.2518424
H	4.9587420	-1.2649217	0.8468927
H	5.6987404	-0.2701954	-0.4009282
C	5.0817783	-1.6361994	-3.7183341
H	5.2977203	-3.5703192	-2.8150634
H	2.8675328	-3.8905028	-0.6364827
H	4.5846233	-4.2586322	-0.8587582
H	4.0560788	-3.1331297	0.4065180
H	4.6302416	0.3770434	-4.3339026
H	1.9658839	1.2367335	-2.1626178
H	3.5142473	1.9124355	-1.6707282
H	3.1245996	1.7900663	-3.3975764
C	1.0046917	-4.6963475	4.1066859
C	4.8897175	4.2028275	2.8120782
C	5.9897962	-1.9340468	-4.8857292
C	0.1728611	-5.7456189	4.0191608
H	1.5297359	-4.5280048	5.0472009
H	4.6843779	4.1404792	3.8883806
H	5.9753363	4.2231387	2.6796511
H	4.4763064	5.1527764	2.4572256
H	6.9658732	-1.4497596	-4.7542332
H	6.1613544	-3.0096329	-4.9875709
H	5.5657209	-1.5588329	-5.8233493
H	0.0254889	-6.4103823	4.8646934
H	-0.3837012	-5.9776230	3.1148238

$PMes_3$: bulky phosphine P(Mesityl)$_3$
61
Energy = -1390.833957518

P	-0.8757657	-0.1676693	0.0113012
C	-2.3765421	0.7194048	-0.5447845
C	-1.3240913	-1.4536801	1.2336640
C	-0.0397476	-0.9369146	-1.4233917
C	-3.0017227	0.6189906	-1.8121413
C	-2.8556137	1.6837666	0.3809685
C	-0.2356208	-1.8994456	2.0296349
C	-2.6255562	-1.9292763	1.5276122
C	0.7172982	-0.0257838	-2.2084344
C	0.0196552	-2.3172613	-1.7317906
C	-4.0483380	1.4925962	-2.1257438
C	-3.9169692	2.5203117	0.0269331
C	-2.2718894	1.8067996	1.7671467
C	-0.4628282	-2.7869440	3.0840828
C	-2.8038280	-2.8013712	2.6070374
C	-3.8442852	-1.5917691	0.7044454
C	1.5062547	-0.5006401	-3.2576590
C	0.6670711	1.4619415	-1.9599384
C	0.8417300	-2.7454780	-2.7809396
C	-4.5203414	2.4551935	-1.2303811
H	-4.5253947	1.3967792	-3.1001097
H	-4.2772244	3.2437376	0.7565961
H	-2.4830237	0.9148584	2.3702043
H	-1.1797958	1.9013820	1.7256194
H	-2.6902418	2.6752815	2.2835483
C	-1.7431598	-3.2462051	3.3992856
H	0.3867312	-3.1214972	3.6773026
H	-3.8103108	-3.1584234	2.8205982

H	-4.6588484	-2.2781395	0.9531634
H	-4.1941900	-0.5688905	0.8777783
H	-3.6368864	-1.6725710	-0.3665777
C	1.5923729	-1.8622964	-3.5585052
H	2.0729376	0.2158897	-3.8502412
H	0.8626262	1.6907704	-0.9051283
H	-0.3255720	1.8706351	-2.1870167
H	1.4006923	1.9797160	-2.5841509
H	0.8778810	-3.8103243	-3.0063376
C	1.1813066	-1.4640072	1.7466999
H	1.5385127	-1.8668355	0.7906456
H	1.2480611	-0.3719631	1.6672914
H	1.8539007	-1.8109251	2.5361368
C	-1.9693688	-4.2184401	4.5306554
H	-2.9067410	-4.0062060	5.0549927
H	-2.0323104	-5.2472963	4.1530233
H	-1.1487311	-4.1808513	5.2533563
C	-0.8011269	-3.3686327	-1.0262631
H	-0.4197087	-3.5926679	-0.0247459
H	-1.8386307	-3.0460183	-0.8997123
H	-0.7940552	-4.2941845	-1.6092151
C	2.4353787	-2.3559320	-4.7082530
H	1.8730398	-2.3110834	-5.6500677
H	3.3323013	-1.7410959	-4.8340913
H	2.7423440	-3.3952466	-4.5566722
C	-2.6436811	-0.4260292	-2.8401421
H	-1.6977694	-0.2056988	-3.3456678
H	-2.5305456	-1.4134565	-2.3832911
H	-3.4304993	-0.4820814	-3.5980538
C	-5.6358955	3.3952423	-1.6142565
H	-5.2537871	4.2242026	-2.2240109
H	-6.4005680	2.8818136	-2.2067013
H	-6.1119502	3.8264338	-0.7285360

PPh$_3$: less bulky phosphine P(C_6H_5)$_3$
34
Energy = -1036.779119811

P	0.0007363	0.0002828	0.9573902
C	-0.3110052	1.6169187	0.1397839
C	-1.2434663	-1.0774890	0.1392222
C	1.5559384	-0.5385398	0.1390849
C	-1.1350317	1.7788524	-0.9824046
C	0.3183740	2.7466989	0.6878202
C	-0.9713343	-1.8728726	-0.9822884
C	-2.5371900	-1.0955524	0.6857961
C	2.2192468	-1.6484380	0.6876358
C	2.1073938	0.0924781	-0.9843730
C	-1.3191463	3.0414256	-1.5490500
H	-1.6319254	0.9154023	-1.4146295
C	0.1447766	4.0053490	0.1138922
H	0.9493986	2.6365365	1.5669704
C	-1.9728454	-2.6629365	-1.5497164
H	0.0252631	-1.8724562	-1.4135656
C	-3.5406712	-1.8744010	0.1110972
H	-2.7576526	-0.4930267	1.5641966
C	3.3950184	-2.1291873	0.1129309
H	1.8087263	-2.1385660	1.5676989
C	3.2919199	-0.3809817	-1.5518203
H	1.6081285	0.9541701	-1.4174621
C	-0.6769730	4.1557792	-1.0062943
H	-1.9617472	3.1530102	-2.4182148
H	0.6420177	4.8693854	0.5459529
C	-3.2596411	-2.6622696	-1.0083476
H	-1.7478699	-3.2758755	-2.4183852
H	-4.5380954	-1.8743985	0.5419380
C	3.9356434	-1.4942254	-1.0084232
H	3.8941724	-2.9920673	0.5450964
H	3.7091848	0.1182022	-2.4222303
H	-0.8194875	5.1371538	-1.4498225
H	-4.0383037	-3.2761629	-1.4521255
H	4.8564469	-1.8621987	-1.4520860

PTo$_3$: bulky phosphine P(*o*-C6H_4CH_3)$_3$
43
Energy = -1154.805466726

P	-1.4424738	-1.0994628	-0.1877858
C	-1.3698371	-1.6113284	1.5750357
C	-2.7579772	-2.1868495	-0.8674776
C	-2.2785497	0.5338524	-0.0973881
C	-2.2448273	-2.5676490	2.1095563
C	-0.3524563	-1.0661023	2.3933505
C	-4.0556468	-1.7185545	-1.1182359
C	-2.4225097	-3.5191587	-1.2055895
C	-2.3809475	1.3045124	-1.2795378
C	-2.7535862	1.0613209	1.1117735
C	-2.1255044	-2.9933167	3.4318865
H	-3.0280572	-2.9815407	1.4809939
C	-0.2511285	-1.5016533	3.7196723
C	0.5915326	-0.0132329	1.8724091
C	-5.0187966	-2.5429095	-1.6985909
H	-4.3118474	-0.6965286	-0.8546461
C	-3.4041907	-4.3339594	-1.7813887
C	-1.0493457	-4.0752249	-0.9295681
C	-2.9553149	2.5786599	-1.2035385
C	-1.9168169	0.7660067	-2.6083437
C	-3.3188182	2.3345959	1.1691390
H	-2.6791655	0.4657110	2.0170485
C	-1.1232837	-2.4578552	4.2410645
H	-2.8134294	-3.7360585	3.8264251
H	0.5310258	-1.0847200	4.3503475
H	0.9467248	-0.2676681	0.8665135
H	0.0883516	0.9587376	1.7934991
H	1.4528283	0.1030651	2.5357597
C	-4.6914205	-3.8573372	-2.0319179
H	-6.0181685	-2.1600290	-1.8862854
H	-3.1491472	-5.3591965	-2.0404981
H	-0.2707217	-3.3567261	-1.2127697
H	-0.9176938	-4.2787877	0.1406792
H	-0.8882453	-5.0089438	-1.4750947
C	-3.4199753	3.0973304	0.0055563

H	-3.0325305	3.1741939	-2.1105757
H	-0.9441289	0.2686962	-2.5121791
H	-2.6189560	0.0156217	-2.9932945
H	-1.8362200	1.5666489	-3.3485440
H	-3.6791944	2.7255421	2.1166759
H	-1.0182663	-2.7826814	5.2726077
H	-5.4324157	-4.5094186	-2.4861838
H	-3.8566317	4.0918137	0.0370897

TSA : transition state for P-N addition
65
Energy = -1800.219878668

P	-0.6280516	-0.3063172	0.0361618
C	-2.3820937	-0.3918447	-0.4331586
C	-0.3833139	-1.5358041	1.3543339
C	0.3551344	-0.8127168	-1.3917742
C	-2.7551614	-0.6633092	-1.7605864
C	-3.3763827	-0.1216363	0.5380055
C	0.3335237	-1.0815155	2.4711117
C	-0.9014268	-2.8517685	1.3256835
C	0.4689218	0.0552778	-2.5030162
C	1.1408875	-1.9782580	-1.3328922
C	-4.0943257	-0.6826268	-2.1357324
H	-1.9891097	-0.8869417	-2.4958223
C	-4.7165091	-0.1538730	0.1356729
C	-3.0339218	0.1649536	1.9768417
C	0.5623058	-1.9148379	3.5619545
H	0.7142176	-0.0673380	2.4468626
C	-0.6551467	-3.6717737	2.4348163
C	-1.7046947	-3.3994868	0.1737198
C	1.3397496	-0.3065090	-3.5404342
C	-0.3022404	1.3469992	-2.6208980
C	2.0033074	-2.3105166	-2.3686428
H	1.0789888	-2.6166416	-0.4572058
C	-5.0812252	-0.4271146	-1.1821077
H	-4.3645696	-0.9037171	-3.1643097
H	-5.4866282	0.0487158	0.8763692
H	-2.7760787	-0.7565029	2.5117056
H	-2.1768374	0.8428019	2.0340875
H	-3.8841780	0.6274327	2.4859488
C	0.0637102	-3.2176053	3.5406428
H	1.1225628	-1.5513858	4.4185396
H	-1.0353879	-4.6905999	2.4236585
H	-1.7149411	-4.4923624	0.2064723
H	-2.7416776	-3.0475513	0.2184334
H	-1.3014986	-3.0815938	-0.7917590
C	2.1010706	-1.4682777	-3.4806989
H	1.4278708	0.3519769	-4.4013301
H	-0.9830933	1.5062829	-1.7807688
H	-0.9026062	1.3568067	-3.5372611
H	0.3939262	2.1896536	-2.6583789
H	2.5976922	-3.2174336	-2.3105064
H	-6.1306640	-0.4384691	-1.4629049
H	0.2346586	-3.8836411	4.3819967
H	2.7759978	-1.7158265	-4.2951168
C	2.8847395	0.7360168	-0.1342749
O	3.9005765	0.7921152	-0.8153943
O	2.6729927	-0.1633497	0.8494971
C	-0.0425549	2.6724405	0.4776052
O	-1.1732336	2.6273790	0.9302272
O	0.5473885	3.7879432	0.0069111
N	0.8894287	1.5840510	0.5435172
N	1.8129766	1.6176343	-0.3718569
C	3.7228576	-1.1526610	1.0621432
C	4.7721325	-0.6292570	2.0271499
H	4.1531103	-1.4099177	0.0916306
H	3.1886671	-2.0120965	1.4726500
H	4.3108820	-0.3343683	2.9747847
H	5.5060283	-1.4172949	2.2289187
H	5.2951555	0.2316914	1.6018922
C	-0.2705877	5.0030984	0.0603932
C	-0.1807166	5.6566187	1.4278387
H	0.1604877	5.6325238	-0.7200645
H	-1.2974985	4.7366537	-0.1977264
H	-0.6184394	5.0115172	2.1942136
H	0.8609709	5.8681501	1.6874027
H	-0.7329627	6.6026213	1.4124664

TSB : transition state for B-N addition
56
Energy = -2854.922182760

C	-0.3540700	-1.5589845	2.4623384
O	-0.7194972	-1.5988388	3.6005851
O	-0.0015728	-2.4735730	1.6073555
C	-1.2168497	1.7659042	3.0950042
O	-2.4081633	1.9237600	2.8899898
O	-0.3878847	2.4408049	3.8780986
N	-0.5618493	0.7597447	2.4593818
N	-0.2253141	-0.1685831	1.7525977
C	0.0566779	-3.8916177	2.0670551
C	1.4742125	-4.3824005	1.8794708
H	-0.6580937	-4.4063005	1.4238431
H	-0.2803628	-3.9159850	3.1030961
H	2.1668233	-3.8433826	2.5309219
H	1.5082996	-5.4448499	2.1409983
H	1.7906704	-4.2711285	0.8390563
C	-0.9599241	3.6533128	4.5178234
C	-0.8455849	4.8376609	3.5807022
H	-1.9907507	3.4313280	4.7960897
H	-0.3472914	3.7644354	5.4116874
H	0.1964308	5.0131805	3.3007066
H	-1.4444144	4.6851488	2.6779457
H	-1.2211049	5.7294253	4.0935424
B	0.2478063	-0.0079573	0.1743802
C	1.7599241	-0.5852944	-0.0130298
C	-0.9501341	-0.8402569	-0.5618614
C	0.3219142	1.5886695	-0.1521217
C	2.6507639	-0.9511821	0.9918236
C	2.2955941	-0.6076207	-1.3029805
C	-2.2637541	-0.3605367	-0.4980069

C	-0.8128924	-2.0652383	-1.2235278
C	-0.2527608	2.2194319	-1.2591161
C	1.1222267	2.4244598	0.6350673
C	3.9603970	-1.3588056	0.7502253
C	3.5954052	-1.0020948	-1.5941877
C	-3.3563542	-0.9963639	-1.0762695
C	-1.8781375	-2.7350828	-1.8178323
C	-0.0928776	3.5753779	-1.5382124
C	1.3015649	3.7824683	0.4007169
C	4.4379063	-1.3875171	-0.5547498
C	-3.1601455	-2.1971203	-1.7490975
C	0.6813865	4.3674834	-0.6982809
F	2.2739451	-0.9193749	2.3001392
F	1.5182701	-0.2347379	-2.3481646
F	-2.5182302	0.7974151	0.1541410
F	0.3842749	-2.6863251	-1.3120821
F	-0.9958025	1.5259871	-2.1512517
F	1.7931636	1.9060738	1.6959110
F	4.7656996	-1.7164917	1.7684882
F	4.0451890	-1.0178815	-2.8624712
F	-4.5891592	-0.4663695	-0.9909451
F	-1.6813239	-3.9062059	-2.4507817
F	-0.6763085	4.1237560	-2.6198254
F	2.0737095	4.5287624	1.2130821
F	5.6979969	-1.7738278	-0.8095617
F	-4.1927604	-2.8342047	-2.3174991
F	0.8418294	5.6750523	-0.9516905
